# Supplementary material for: Age at Natural Menopause and Blood Pressure Traits: Mendelian Randomization Study
Source: J Clin Med. 2021 Sep 22;10(19):4299. doi: 10.3390/jcm10194299 (PMC8509463; doi:10.3390/jcm10194299)
Supplement: Supplementary file 1 [file jcm-10-04299-s001.zip › jcm-1350523-supplementary.pdf]

Supplementary Materials

List Online tables

Table S1. Age of menopause SNPs and alleles used for the genetic risk score ... 6
Table S2. Observational association between age of natural menopause and blood pressure traits Log transformed ... 8
Table S3. Characteristics of participants without imputation and missing values by study ... 9
Table S4. Allele harmonization from GWAS of age of natural menopause and UK BIOBANK. ... 11
Table S5. Observational association between age of natural menopause and blood pressure traits ... 15
Table S6. P values for interaction between age of natural menopause and Body Mass Index ... 19
Table S7. Comparison of estimates Rotterdam study imputed vs without imputation ... 20
Table S8. Sensitivity analysis observational estimates of the association between categories of anm and bp traits ... 21
Table S9. Association Genetic Risk Score of age of natural menopause (ANM) AND OBSERVED ANM ... 22
Table S10. Association of weighted Genetic Risk Score with blood pressure traits by study ... 23
Table S11. Association of the Genetic Risk Score with confounders by study ... 25
Table S12. Association of each SNP included in the Genetic Risk Score with confounders by study ... 26
Table S13. Association of each SNP included in the Genetic Risk Score with blood pressure traits by study. ... 30
Table S14. Causal Estimates Derived from Instrumental Variable Analysis Genetic Risk Score with SNPs related and non-related to DNA damage-response (DDR) genes. ... 32
Table S15. Outlier SNPs identified in the MR-PRESSO ... 33
Table S16. Causal estimates of ANM in Systolic and diastolic blood pressure from SNPs related and non-related to DDR genes using methods implemented in MR-Base with data from GWAS of ANM and UK Biobank ... 33
Table S17. Identification of pleiotropic SNPs ... 34
Table S18. Two-sample Mendelian Randomization excluding SNPs associated with BMI and menarche ... 35
Table S19. Two-sample Mendelian Randomization including all SNPs ... 36

List Online Figures

Figure S1. Flow chart of Study sample 37
Figure S2. non-linear spline models 38
Figure S3. Sensitivity analysis observational association ANM and Blood pressure traits 39
Figure S4. Association of Genetic Risk Score of ANM after the "leave-one-out" analyses. 43
Figure S5. Causal Estimates Derived from Instrumental Variable Analysis for a year Increase in ANM and Risk of blood pressure traits using Individual Participant Data and excluding the SNP rs1054875 48
Figure S6. Funnel plot of causal association between ANM and BP. 50
Figure S7. Mendelian randomization diagnostic plots 51
Figure S8. Leave-one-out plot to assess if a single variant is driving the association between ANM and BP 52
Figure S9. Scatter plots, leave-one-out, funnel plots SNPs related to DDR genes 54
Figure S10. Scatter plots, leave-one-out, funnel plots SNPs non-related to DDR genes 57

Expanded Materials & Methods

Study population

CoLaus is a population-based study in Caucasians 35-75 years old living in the city of Lausanne, Switzerland. At baseline (2003-2006), 6,189 participants were included. The design of the study has been described elsewhere(1). In this study, postmenopausal women from the baseline were included. The Institutional Ethics Committee of the University of Lausanne - Switzerland, approved CoLaus; written informed consent was obtained from all participants.

The Rotterdam Study (RS) is a population-based cohort study of individuals 45 years and over living in the Ommoord district of Rotterdam, the Netherlands. The rationale and design of RS is described elsewhere(2). In brief, at baseline (1990-1993), 7,983 participants aged 55 years and over were included (RS-I). In 2000, an additional 3011 participants 55+ years were enrolled (RS-II). A second extension of the cohort was initiated in 2006, in which 3,932 participants aged 45 years or older were included (RS-III). The present study includes data from postmenopausal women from the third visit of the first cohort of RS (RS-I-3), the first visits of the second (RS-II-1) and first visit of the third cohort (RS-III-1). The RS has been approved by the medical ethics committee according to the Population Study Act Rotterdam Study, executed by the Ministry of Health, Welfare and Sports of the Netherlands. A written informed consent was obtained from all participants.

Assessment of BP traits

In CoLaus, BP was measured thrice on the left arm with an appropriately sized cuff, after at least 10-minutes rest in the seated position using an Omron® HEM-907 automated oscillometric sphygmomanometer. In RS, BP was measured twice on the right arm (cuff size of 32×17), after a resting period of 5-minutes rest in the seated position using a random-zero sphygmomanometer. In the UK Biobank BP was measured twice in a seated position after 2-minutes of rest using an Omron HEM-7015IT digital BP monitor and an appropriate cuff (3). SBP was recorded at the appearance of sounds (first-phase Korotkoff) and DBP at the disappearance of sounds (fifth-phase Korotkoff).

In all cohorts, SBP and DBP were calculated as the average of two measurements, in CoLaus only the last two measurement were used. Also in CoLaus and RS, hypertension was defined as a SBP ≥140 mmHg, and/or DBP ≥90 mmHg, and/or the use of antihypertensive medication. Medication use information was based on the home interview in RS and self-filled questionnaires in CoLaus. Antihypertensive medication use was defined as diuretics, β-blockers, angiotensin-converting enzyme inhibitors, and calcium channel blockers. In the RS, a physician also ascertained the indication for which the medication had been prescribed.

Assessment of covariates

Sociodemographic data, socio-economic, and lifestyle factors were recorded during the interview in both CoLaus and RS, and data from both studies were harmonized. Education was defined as low (primary education), intermediate (secondary general or vocational education), or high (higher vocational education or university). Alcohol intake was assessed in grams of ethanol per day, and participants were classified as drinkers vs. non-drinkers. Similarly, smoking status was dichotomized into current/ever smokers vs. never smokers. History of cardiovascular disease was defined as a history of coronary heart disease (myocardial infarction, revascularization, coronary artery bypass graft surgery or percutaneous coronary intervention) and was verified from the medical records of the general practitioner. Diabetes mellitus was defined as the use of blood glucose–lowering medications or fasting glucose ≥7.0 mmol/L. Estimated glomerular filtration rate (eGFR) was calculated using the simplified Modification of Diet in Renal Disease equation. Physical height (m) and body weight (kg) were measured at baseline with the participants standing without

shoes and heavy outer garments. Body mass index (BMI) was calculated as weight divided by height squared (kg/m^2). All biochemical parameters were assessed in fasting serum.

Genotyping

In CoLaus, nuclear DNA was extracted from whole blood for whole genome scan analysis and genotyping was performed using the Affimetrix 500 K SNP chip, as recommended by the manufacturer. Subjects were excluded from the analysis in case of inconsistency between sex and genetic data, a genotype call rate <90%, or inconsistencies of genotyping results in duplicate samples. Quality control for SNPs was performed using the following criteria: monomorphic (or with minor allele frequency (MAF) <1%), call rates <90%, deviation from the Hardy-Weinberg equilibrium ($p < 1 \times 10^{-6}$). Phased haplotypes were generated using SHAPEIT2(4). Imputation was performed using minimac3(5) and the Haplotype Reference Consortium (HRC version r1.1)(6) hosted on the Michigan Imputation Server(5).

In the RS, genotyping was conducted, in self-reported white participants, using the Illumina 550K array. Participants were excluded if they had excess autosomal heterozygosity, a mismatch between the reported and phenotypic sex, or recognized as being an outlier with identical-by-state clustering analysis. Moreover, SNPs with allele frequency ≤1%, Hardy–Weinberg equilibrium $p<10^{-5}$, or SNP call rate ≤90% were excluded. Imputation was done with reference to HapMap release 22 CEU (Utah residents of northern and western European ancestry) using the maximum likelihood method implemented in Markov Chain based haplotyper (version 1.0.15).

Genetic risk score

For each SNP included in the genetic risk score of the ANM (GRS-ANM), the number of corresponding coded alleles was multiplied by the absolute value of the beta-coefficient for ANM reported in the reference GWAS(7), and then these products were summed up. Successively, to produce an average measure, the GRS-ANM was divided by the number of effectively genotyped SNPs, then the product was multiplied by 100 to rescale the scores to a range between 0 and 100(8).

Statistical Analyses

Cross-sectional analyses

In the model 4 we calculated the variance inflation factor (VIF) to evaluate multicollinearity among the covariates included; none of them presented a value greater than 10 (9). We conducted the diagnostics of the final linear and logistic regression models by evaluating the residuals and we identified three observations with large leverage in the CoLaus data; these observations were excluded from the final model investigating the association between ANM and DBP. Interaction of ANM and BMI were evaluated in model 4 by including a cross-product interaction term. Furthermore, we fitted non-linear (spline) models, using the "mkspline" package with restricted cubic spline construction (4 knots) in Stata, to explore nonlinear relationship between ANM and SBP, DBP and hypertension. In CoLaus, ANM, eGFR and BMI were nonlinear in the logit scale, which is the link function where the probabilities of the levels of hypertension status are transform to a continuous scale to be modeled as a linear association between the exposures and the outcome. Therefore, fractional polynomial were explored; nevertheless, none of them improved the model.

ANM, SBP and DBP did not follow a normal distribution; thus, a log transformation was tested (Table S2). However, results did not change and residuals followed the assumptions; as a result, the analysis were reported with the untransformed variables. To adjust for potential biases associated with missing data from the covariates, we used multiple imputation procedure (n=10 imputations) in the RS's datasets; most of the variables had less than 7% of missing values, nevertheless alcohol consumption had up to 64% of missing values. Only two participants with missing data on BMI and

educational level were present in CoLaus study; thus, no multiple imputation was applied for analyses in CoLaus (Table S3).

One sample Mendelian randomization

Association of the GRS-ANM and BP/ hypertension

The first model adjusted for age and eGFR. In the second model, we additionally adjusted for antihypertensive medication, BMI, drinking status, diabetes, history of cardiovascular diseases, total cholesterol, smoking status, statin use, hormone therapy and education level; when hypertension was the outcome, we did not adjust for antihypertensive medications.

Mendelian randomization relies on three assumptions, first, the GRS-ANM is associated with the ANM, second, there are no unmeasured confounders of the association between GRS-ANM and BP traits, and third, the GRS-ANM affect the outcome only through their effect on the ANM(10). In order to explore the first assumption, we calculated the partial "F" statistics and partial "R^2" for the association between GRS-ANM and ANM. Regarding the second and third assumption, we evaluated the association of each SNP with BP traits, and confounders. The cut-off for significance was established using the Bonferroni correction. To further investigate if a single SNP was influencing the associations, we performed a leave-one-out analysis by creating GRSs that excluded one SNP at a time(11).

Two sample Mendelian Randomization

IVM assumes that all variants are valid instruments, and any pleiotropy is balanced. The MR-Egger was implemented under the Instrument Strength Independent of Direct Effect (InSIDE) assumption, the intercept of a weighted regression will be other than zero in the existence of directional pleiotropy, and the slope of that regression will be a consistent estimate of the causal effect of X on Y. The weighted median method is robust to outliers (up to 50% of invalid instrumental variables), while MR-PRESSO addresses the between variants heterogeneity and horizontal pleiotropy(11).

Author contributions

TM conceived the study. ZMRD, EA, PMV, TV and TM designed the study. TM supervised the study. ZMRV, EA, HA, LZR, JN, MAI, FD, OHF, RP, PDM, TV and TM participated in data acquisition, collection, analysis or interpretation. ZMRD, EA, HA, FD and RP performed the statistical analyses. ZMRD, EA, PMV, TV and TM drafted the manuscript; HA, LZR, JN, MAI, FD, OHF, and RP critically revised the manuscript for intellectual content. All authors approved the final version of the manuscript. ZMRD and TM are the guarantors of the study and responsible for the integrity of the work as a whole.

TABLE S1. AGE OF MENOPAUSE SNPS AND ALLELES USED FOR THE GENETIC RISK SCORE

n	Region	Signal SNP	Chr	Position	Alleles
1	1	rs4246511	1	39,152,972	c/t/0.71
2	2	rs12142240	1	46,519,888	t/c/0.68
3	3	rs1411478	1	179,228,905	a/g/0.41
4	4	rs2236918	1	240,084,449	c/g/0.45
5	5	rs704795	2	27,569,998	a/g/0.4
6	6	rs1800932	2	47,871,585	a/g/0.81
7	7	rs930036	2	171,649,264	a/g/0.38
8	8	rs16858210	3	185,106,704	g/a/0.75
9	9	rs4693089	4	84,592,646	a/g/0.51
10	10	rs6856693	4	185,985,800	a/g/0.58
11	11	rs427394	5	6,798,875	g/a/0.41
12	12	rs11738223	5	171,867,097	a/g/0.68
13	13a	rs2241584	5	175,888,783	a/g/0.38
14	13b	rs365132	5	176,311,180	g/t/0.51
15	14a	rs6899676	6	11,003,246	a/g/0.8
16	14b	rs9393800	6	11,059,723	g/a/0.27
17	15a	rs2230365	6	31,633,427	c/t/0.84
18	15b	rs707938	6	31,837,338	g/a/0.32
19	16	rs12196873	6	111,704,751	a/c/0.85
20	17	rs2720044	8	38,099,744	a/c/0.84
21	18	rs10957156	8	61,791,955	a/g/0.76
22	19	rs4879656	9	33,002,382	a/c/0.37
23	20	rs10905065	10	5,809,833	a/g/0.61
24	21a	rs11031006	11	30,183,104	g/a/0.85
25	21b	rs6484478	11	30,263,016	g/a/0.74
26	22	rs10734411	11	32,498,360	a/g/0.47
27	23	rs2277339	12	55,432,336	g/t/0.1
28	24a	rs3741604	12	64,982,677	t/c/0.52
29	24b	rs1183272	12	65,021,688	c/t/0.45
30	24c	rs7397861	12	65,100,733	g/c/0.64
31	25	rs551087	12	119,693,576	g/a/0.29
32	26	rs1727326	12	122,166,039	c/g/0.15
33	27	rs12824058	12	129,370,287	g/a/0.43
34	28	rs4886238	13	60,011,740	g/a/0.66
35	29	rs1713460	14	20,003,455	g/a/0.3
36	30	rs9796	15	39,058,739	t/a/0.46
37	31	rs1054875	15	87,680,130	t/a/0.4
38	32	rs9039	16	9,112,864	c/t/0.28
39	33	rs10852344	16	11,924,420	t/c/0.59
40	34	rs12599106	16	34,355,526	a/t/0.51
41	35	rs8070740	17	5,272,620	a/g/0.76
42	36	rs2941505	17	35,086,230	a/g/0.32
43	37	rs1799949	17	38,498,992	g/a/0.68
44	38	rs349306	19	901,694	g/a/0.13
45	39	rs7259376	19	22,299,545	a/g/0.46

n	Region	Signal SNP	Chr	Position	Alleles
46	40a	rs11668344	19	60,525,476	g/a/0.36
47	40b	rs2547274	19	61,002,040	g/c/0.91
48	40c	rs12461110	19	61,012,475	a/g/0.35
49	41a	rs451417	20	5,889,999	a/c/0.12
50	41b	rs16991615	20	5,896,227	g/a/0.93
51	42a	rs2236553	20	60,760,188	c/t/0.24
52	42b	rs13040088	20	61,019,647	g/a/0.21
53	43	rs5762534	22	26,963,571	t/c/0.84
54	44	rs763121	22	37,209,886	g/a/0.36

Chr = Chromosome

TABLE S2. OBSERVATIONAL ASSOCIATION BETWEEN AGE OF NATURAL MENOPAUSE AND BLOOD PRESSURE TRAITS LOG TRANSFORMED

ANM	SBP		DBP		Hypertension	
	β(95% CI)	p	β(95% CI)	p	OR(95% CI)	p
CoLaus	0.01(-0.08;0.10)	0.861	0.09(0.00;0.18)	0.043	2.17(0.46;10.27)	0.327
RS-I-3	0.05(-0.04;0.14)	0.27	-0.04(-0.12;0.05)	0.438	2.83(0.67;11.87)	0.155
RS-II-1	-0.01(-0.12;0.11)	0.925	-0.03(-0.15;0.09)	0.607	3.89(0.49;30.64)	0.197
RS-III-1	0.16(0.04;0.29)	0.008	0.10(-0.01;0.22)	0.074	3.75(0.56;25.15)	0.174

SBP = Systolic blood pressure; DBP = Diastolic blood pressure; OR = Odds ratio; CI=Confidence interval; p = p value

Models adjusted for age and glomerular filtration rate, body mass index, total cholesterol, drinking status, education level, smoking status, prevalent Type 2 diabetes, history of cardiovascular diseases, statin use, hormone replacement therapy, antihypertensive medication

TABLE S3. CHARACTERISTICS OF PARTICIPANTS WITHOUT IMPUTATION AND MISSING VALUES BY STUDY

Study	CoLaus (n=1139)			RS-I-3 (n=1603)			RS-II-1 (n=790)			RS-III-1 (n=919)		
Variable	I n(%)	NI n (%)	MV n(%)	I n(%)	NI n (%)	MV n(%)	I n(%)	NI n (%)	MV n(%)	I n(%)	NI n (%)	MV n(%)
Age, years mean (SD) y	61.2 (7.0)	61.2(7.0)	0	73.2 (7.5)	73.2(7.5)	0	65.4 (8.4)	65.4(8.4)	0	59.7 (6.5)	59.7(6.5)	0
BMI, Kg/m2 ,mean (SD)	25.6 (4.9)	25.6(4.9)	1(0.1)	27.2 (4.4)	27.2(4.4)	45(2.8)	27.3 (4.3)	27.3(4.3)	3(0.4)	27.4 (4.8)	27.4(4.8)	4(0.4)
Categories of ANM (%)												
Early (40-44)	111 (9.7)	111(9.7)		151 (9.4)	151 (9.4)		68 (8.7)	68(8.7)		63 (6.8)	63(6.8)	
Intermediate (45-49)	308 (27.0)	308(27.0)	0	411 (35.1)	411 (35.1)	0	178 (22.5)	178(22.5)	0	213 (23.2)	213 (23.2)	0
Normal (50-54)	549 (48.2)	549(48.2)		876 (54.7)	876 (54.7)		407 (51.5)	407 (51.5)		512 (55.7)	512(55.7)	
Late (≥55)	171 (15.0)	171(15.0)		165 (10.3)	165 (10.3)		137 (17.3)	137(17.3)		131 (14.2)	131(14.2)	
Smoking status												
No	565(49.6)	565(49.6)	0	1364(85.1)	1331(84.8)	33(2.0)	643(81.4)	643(81.4)	0	702(76.4)	701(76.3)	1(0.1)
Yes	574 (50.4)	574(50.4)		239 (14.9)	239(15.2)		147 (18.6)	147(18.6)		217 (23.6)	211(23.7)	
Alcohol drinker												
No	401(35.2)	401(35.2)	0	402(25.1)	396(25.2)	31(1.9)	275(34.8)	157(55.8)	509(64)	37(4.0)	13(1.8)	194(21)
Yes	738 (64.8)	738 (64.8)		1201 (74.9)	1176(74.8)		515 (65.2)	124(44.1)		882 (95.9)	712(98.2)	
Educational level (%)												
High	123 (10.8)	123(10.8)	1(0.1)	79 (4.9)	76(4.8)	15(0.9)	77 (9.8)	77(9.8)	4(0.5)	171 (18.6)	171(18.7)	2(0.2)
Middle	280 (24.6)	280(24.6)		399 (24.9)	396(24.9)		159 (20.1)	158(20.1)		170 (18.5)	170(18.5)	
Low	735 (64.7)	735(64.7)		1125 (70.2)	1116(70.3)		554 (70.1)	551(70.1)		578 (62.9)	576(62.7)	
Assessed physical traits, mean (SD)												
Total cholesterol, nmol/L, mean (SD)	5.9 (0.99)	5.9(0.99)	0	6.0 (1.0)	6.0(1.0)	89(5.5)	6.0 (1.0)	6.0(1.0)	3(0.4)	5.8 (1.0)	5.8(1.0)	4(0.43)
eGFR mL/min per 1.73 m2, mean (SD)	78.0 (13.7)	78.0(13.7)	0	68.9 (14.5)	68.9(14.5)	114(7.1)	79.7 (15)	79.7(15)	25(3.2)	82.6 (13.2)	82.6(13.2)	47(5.1)
Systolic BP, mm Hg, mean (SD)	130.8 (18.7)	130.8(18.7)	0	143.5 (21.3)	143.5(21.3)	0	142.4 (21.5)	142.4(21.5)	0	132.0 (20.0)	132.0(20.0)	0
Diastolic BP, mm Hg, mean (SD)	78.7 (10.4)	78.7(10.4)	0	74.2 (10.9)	74.2(10.9)	0	77.2 (10.3)	77.2(10.3)	0	84.4 (11.2)	84.4(11.2)	0
Hypertension	492 (43.2)	492(43.2)	0	1110 (69.3)	1110(69.3)	0	492 (62.3)	492(62.3)	0	454 (49.5)	454(49.5)	0

Diabetes	55 (5.0)	55(5.0)	0	200 (12.5)	192(12.6)	74(4.6)	79 (10.0)	79(10.0)	0	62 (6.7)	62(6.7)	1(0.1)
History of cardiovascular diseases	85 (7.5)	85(7.5)	0	176 (11.0)	175(11.0)	14(0.9)	40 (5.1)	40(5.1)	4(0.5)	25 (2.74)	25(2.74)	7(0.8)
Receiving BP-related medication	262 (23.0)	264(23.2)	0	614 (38.3)	614(38.3)	0	226 (28.6)	226(28.6)	0	223 (24.2)	223(24.2)	0
Hormone therapy	638 (56.0)	638(56.0)	0	35 (2.3)	35(2.3)	102(6.3)	31 (3.9)	31(3.9)	0	16 (1.7)	16(1.7)	5(0.5)
Statin therapy	141 (12.4)	141(12.4)	0	206 (12.8)	189(12.6)	102(6.3)	94 (11.9)	94(11.9)	0	201 (21.9)	201(21.9)	5(0.5)

I = Imputed; NI= Not-imputed; MV = Missing values; BMI = Body mass index; eGFR = estimated glomerular filtration rate; n = number of participants; SD = standard deviation; y = years; BP = blood pressure

TABLE S4. ALLELE HARMONIZATION FROM GWAS OF AGE OF NATURAL MENOPAUSE AND UK BIOBANK.

Pre-harmonisation										Post-harmonisation									
Age natural menopause					Diastolic blood pressure					Age natural menopause					Diastolic blood pressure				
SNP	A1	A2	Beta	se	SNP	A 1	A 2	Beta	SE	SNP	a1	a2	beta	se	SNP	A 1	A2	Beta	SE
rs1054875	T	A	-0.19	0.02	rs1054875	T	A	-0.0723682	0.0394608	rs1054875	T	A	-0.19	0.02	rs1054875	T	A	-0.07237	0.03946
rs10734411	A	G	-0.12	0.02	rs10734411	A	G	0.0679943	0.0382846	rs10734411	A	G	-0.12	0.02	rs10734411	A	G	0.06799	0.03828
rs10852344	T	C	-0.16	0.02	rs10852344	C	T	0.148183	0.0390494	rs10852344	T	C	-0.16	0.02	rs10852344	T	C	-0.14818	0.03905
rs10905065	A	G	-0.11	0.02	rs10905065	G	A	0.0221999	0.039216	rs10905065	A	G	-0.11	0.02	rs10905065	A	G	-0.02220	0.03922
rs10957156	A	G	-0.14	0.02	rs10957156	G	A	-0.0101337	0.0443606	rs10957156	A	G	-0.14	0.02	rs10957156	A	G	0.01013	0.04436
rs11031006	G	A	-0.22	0.03	rs11031006	A	G	-0.0935055	0.0548423	rs11031006	G	A	-0.22	0.03	rs11031006	G	A	0.09351	0.05484
rs11668344	G	A	-0.41	0.02	rs11668344	G	A	0.0118139	0.0399256	rs11668344	G	A	-0.41	0.02	rs11668344	G	A	0.01181	0.03993
rs11738223	A	G	-0.12	0.02	rs11738223	G	A	0.0280688	0.0406686	rs11738223	A	G	-0.12	0.02	rs11738223	A	G	-0.02807	0.04067
rs1183272	C	T	-0.07	0.02	rs1183272	C	T	0.0274983	0.0384904	rs1183272	C	T	-0.07	0.02	rs1183272	C	T	0.02750	0.03849
rs12142240	T	C	-0.13	0.02	rs12142240	C	T	-0.025963	0.0412346	rs12142240	T	C	-0.13	0.02	rs12142240	T	C	0.02596	0.04123
rs12196873	A	C	-0.16	0.03	rs12196873	C	A	0.00379956	0.0545896	rs12196873	A	C	-0.16	0.03	rs12196873	A	C	-0.00380	0.05459
rs12461110	A	G	-0.17	0.02	rs12461110	A	G	0.000308302	0.0397769	rs12461110	A	G	-0.17	0.02	rs12461110	A	G	0.00031	0.03978
rs12599106	A	T	-0.12	0.02	rs12599106	T	A	-0.0436909	0.0383738	rs12599106	A	T	-0.12	0.02	rs12599106	A	T	0.04369	0.03837
rs12824058	G	A	-0.14	0.02	rs12824058	G	A	0.0619927	0.0390647	rs12824058	G	A	-0.14	0.02	rs12824058	G	A	0.06199	0.03906
rs13040088	G	A	-0.16	0.02	rs13040088	G	A	-0.00223773	0.0469394	rs13040088	G	A	-0.16	0.02	rs13040088	G	A	-0.00224	0.04694
rs1411478	A	G	-0.13	0.02	rs1411478	A	G	0.0218985	0.0390415	rs1411478	A	G	-0.13	0.02	rs1411478	A	G	0.02190	0.03904
rs16858210	G	A	-0.14	0.02	rs16858210	A	G	0.104604	0.0446923	rs16858210	G	A	-0.14	0.02	rs16858210	G	A	-0.10460	0.04469
rs16991615	G	A	-0.88	0.04	rs16991615	A	G	0.0849204	0.0776969	rs16991615	G	A	-0.88	0.04	rs16991615	G	A	-0.08492	0.07770
rs1713460	G	A	-0.14	0.02	rs1713460	G	A	0.0174353	0.0412314	rs1713460	G	A	-0.14	0.02	rs1713460	G	A	0.01744	0.04123
rs1727326	C	G	-0.19	0.03	rs1727326	C	G	-0.190348	0.0556898	rs1727326	C	G	-0.19	0.03	rs1727326	C	G	-0.19035	0.05569
rs1799949	G	A	-0.14	0.02	rs1799949	A	G	-0.0622116	0.0408268	rs1799949	G	A	-0.14	0.02	rs1799949	G	A	0.06221	0.04083
rs1800932	A	G	-0.17	0.03	rs1800932	G	A	0.149384	0.0484393	rs1800932	A	G	-0.17	0.03	rs1800932	A	G	-0.14938	0.04844
rs2230365	C	T	-0.17	0.03	rs2230365	T	C	0.123302	0.0550528	rs2230365	C	T	-0.17	0.03	rs2230365	C	T	-0.12330	0.05505
rs2236553	C	T	-0.16	0.03	rs2236553	C	T	0.0355651	0.0451348	rs2236553	C	T	-0.16	0.03	rs2236553	C	T	0.03557	0.04513
rs2236918	C	G	-0.15	0.02	rs2236918	C	G	-0.0958727	0.0385286	rs2236918	C	G	-0.15	0.02	rs2236918	C	G	-0.09587	0.03853
rs2241584	A	G	-0.14	0.02	rs2241584	A	G	0.0572729	0.0397824	rs2241584	A	G	-0.14	0.02	rs2241584	A	G	0.05727	0.03978
rs2277339	G	T	-0.31	0.03	rs2277339	G	T	-0.117926	0.0631604	rs2277339	G	T	-0.31	0.03	rs2277339	G	T	-0.11793	0.06316

Pre-harmonisation										Post-harmonisation									
Age natural menopause					Diastolic blood pressure					Age natural menopause					Diastolic blood pressure				
SNP	A1	A2	Beta	se	SNP	A1	A2	Beta	SE	SNP	a1	a2	beta	se	SNP	A1	A2	Beta	SE
rs2547274	G	C	-0.28	0.04	rs2547274	C	G	-0.0624217	0.0662751	rs2547274	G	C	-0.28	0.04	rs2547274	G	C	0.06242	0.06628
rs2720044	A	C	-0.29	0.03	rs2720044	C	A	-0.00259295	0.0517883	rs2720044	A	C	-0.29	0.03	rs2720044	A	C	0.00259	0.05179
rs2941505	A	G	-0.13	0.02	rs2941505	A	G	0.0541778	0.0412672	rs2941505	A	G	-0.13	0.02	rs2941505	A	G	0.05418	0.04127
rs349306	G	A	-0.23	0.04	rs349306	G	A	0.032006	0.0588684	rs349306	G	A	-0.23	0.04	rs349306	G	A	0.03201	0.05887
rs365132	G	T	-0.24	0.02	rs365132	T	G	0.0272027	0.0383385	rs365132	G	T	-0.24	0.02	rs365132	G	T	-0.02720	0.03834
rs3741604	T	C	-0.09	0.02	rs3741604	C	T	0.0213437	0.0384137	rs3741604	T	C	-0.09	0.02	rs3741604	T	C	-0.02134	0.03841
rs4246511	C	T	-0.22	0.02	rs4246511	T	C	0.053419	0.0425499	rs4246511	C	T	-0.22	0.02	rs4246511	C	T	-0.05342	0.04255
rs427394	G	A	-0.13	0.02	rs427394	G	A	-0.0388071	0.03892	rs427394	G	A	-0.13	0.02	rs427394	G	A	-0.03881	0.03892
rs451417	A	C	-0.2	0.03	rs451417	A	C	0.0169453	0.0587391	rs451417	A	C	-0.2	0.03	rs451417	A	C	0.01695	0.05874
rs4693089	A	G	-0.2	0.02	rs4693089	G	A	0.0152142	0.0382873	rs4693089	A	G	-0.2	0.02	rs4693089	A	G	-0.01521	0.03829
rs4879656	A	C	-0.12	0.02	rs4879656	A	C	0.0678728	0.0395141	rs4879656	A	C	-0.12	0.02	rs4879656	A	C	0.06787	0.03951
rs4886238	G	A	-0.18	0.02	Excluded QC					rs4886238	A	A	-0.18	0.02	rs4886238	Excluded QC			
rs551087	G	A	-0.13	0.02	rs551087	G	A	0.0488124	0.0423673	rs551087	G	A	-0.13	0.02	rs551087	G	A	0.04881	0.04237
rs5762534	T	C	-0.16	0.03	rs5762534	C	T	0.104779	0.0539062	rs5762534	T	C	-0.16	0.03	rs5762534	T	C	-0.10478	0.05391
rs6484478	G	A	-0.1	0.02	rs6484478	A	G	0.035815	0.0455432	rs6484478	G	A	-0.1	0.02	rs6484478	G	A	-0.03582	0.04554
rs6856693	A	G	-0.16	0.02	rs6856693	G	A	-0.0760902	0.038678	rs6856693	A	G	-0.16	0.02	rs6856693	A	G	0.07609	0.03868
rs6899676	A	G	-0.23	0.03	rs6899676	G	A	0.00793723	0.0479084	rs6899676	A	G	-0.23	0.03	rs6899676	A	G	-0.00794	0.04791
rs704795	A	G	-0.16	0.02	rs704795	A	G	-0.0733563	0.0391111	rs704795	A	G	-0.16	0.02	rs704795	A	G	-0.07336	0.03911
rs707938	G	A	-0.17	0.02	rs707938	G	A	-0.178388	0.0410044	rs707938	G	A	-0.17	0.02	rs707938	G	A	-0.17839	0.04100
rs7259376	A	G	-0.11	0.02	rs7259376	A	G	-0.01968	0.038394	rs7259376	A	G	-0.11	0.02	rs7259376	A	G	-0.01968	0.03839
rs7397861	G	C	-0.1	0.02	rs7397861	C	G	0.0213642	0.0396937	rs7397861	G	C	-0.1	0.02	rs7397861	G	C	-0.02136	0.03969
rs763121	G	A	-0.16	0.02	rs763121	G	A	-0.0613941	0.0403425	rs763121	G	A	-0.16	0.02	rs763121	G	A	-0.06139	0.04034
rs8070740	A	G	-0.15	0.02	rs8070740	G	A	-0.0066124	0.044759	rs8070740	A	G	-0.15	0.02	rs8070740	A	G	0.00661	0.04476
rs9039	C	T	-0.12	0.02	rs9039	C	T	0.0517817	0.0431436	rs9039	C	T	-0.12	0.02	rs9039	C	T	0.05178	0.04314
rs930036	A	G	-0.19	0.02	rs930036	A	G	0.136959	0.0393574	rs930036	A	G	-0.19	0.02	rs930036	A	G	0.13696	0.03936
rs9393800	G	A	-0.17	0.02	rs9393800	G	A	-0.0495074	0.0435915	rs9393800	G	A	-0.17	0.02	rs9393800	G	A	-0.04951	0.04359
rs9796	T	A	-0.13	0.02	rs9796	T	A	0.199368	0.0383883	rs9796	T	A	-0.13	0.02	rs9796	T	A	0.19937	0.03839

Pre-harmonisation Post-harmonisation

Pre-harmonisation										Post-harmonisation									
Age natural menopause					Diastolic blood pressure					Age natural menopause					Diastolic blood pressure				
SNP	A1	A2	Beta	se	SNP	A 1	A 2	Beta	SE	SNP	a1	a2	beta	se	SNP	A 1	A2	Beta	SE
Age natural menopause					Systolic blood pressure					Age natural menopause					Systolic blood pressure				
SNP	A1	A2	beta	se	SNP	A1	A2	Beta	SE	SNP	A1	A2	beta	se	SNP	A1	A2	Beta	SE
rs1054875	T	A	-0.19	0.02	rs1054875	T	A	-0.11526	0.06477	rs1054875	T	A	-0.19	0.02	rs1054875	T	A	-0.11526	0.06477
rs10734411	A	G	-0.12	0.02	rs10734411	A	G	0.12274	0.06284	rs10734411	A	G	-0.12	0.02	rs10734411	A	G	0.12274	0.06284
rs10852344	T	C	-0.16	0.02	rs10852344	C	T	0.24950	0.06410	rs10852344	T	C	-0.16	0.02	rs10852344	T	C	-0.24950	0.06410
rs10905065	A	G	-0.11	0.02	rs10905065	G	A	0.02862	0.06437	rs10905065	A	G	-0.11	0.02	rs10905065	A	G	-0.02862	0.06437
rs10957156	A	G	-0.14	0.02	rs10957156	G	A	0.17936	0.07281	rs10957156	A	G	-0.14	0.02	rs10957156	A	G	-0.17936	0.07281
rs11031006	G	A	-0.22	0.03	rs11031006	A	G	-0.21563	0.09002	rs11031006	G	A	-0.22	0.03	rs11031006	G	A	0.21563	0.09002
rs11668344	G	A	-0.41	0.02	rs11668344	G	A	-0.04084	0.06553	rs11668344	G	A	-0.41	0.02	rs11668344	G	A	-0.04084	0.06553
rs11738223	A	G	-0.12	0.02	rs11738223	G	A	0.01652	0.06675	rs11738223	A	G	-0.12	0.02	rs11738223	A	G	-0.01652	0.06675
rs1183272	C	T	-0.07	0.02	rs1183272	C	T	-0.02591	0.06318	rs1183272	C	T	-0.07	0.02	rs1183272	C	T	-0.02591	0.06318
rs12142240	T	C	-0.13	0.02	rs12142240	C	T	0.09646	0.06768	rs12142240	T	C	-0.13	0.02	rs12142240	T	C	-0.09646	0.06768
rs12196873	A	C	-0.16	0.03	rs12196873	C	A	0.13502	0.08960	rs12196873	A	C	-0.16	0.03	rs12196873	A	C	-0.13502	0.08960
rs12461110	A	G	-0.17	0.02	rs12461110	A	G	-0.06872	0.06529	rs12461110	A	G	-0.17	0.02	rs12461110	A	G	-0.06872	0.06529
rs12599106	A	T	-0.12	0.02	rs12599106	T	A	0.03619	0.06299	rs12599106	A	T	-0.12	0.02	rs12599106	A	T	-0.03619	0.06299
rs12824058	G	A	-0.14	0.02	rs12824058	G	A	0.09007	0.06412	rs12824058	G	A	-0.14	0.02	rs12824058	G	A	0.09007	0.06412
rs13040088	G	A	-0.16	0.02	rs13040088	G	A	0.01994	0.07705	rs13040088	G	A	-0.16	0.02	rs13040088	G	A	0.01994	0.07705
rs1411478	A	G	-0.13	0.02	rs1411478	A	G	-0.07501	0.06408	rs1411478	A	G	-0.13	0.02	rs1411478	A	G	-0.07501	0.06408
rs16858210	G	A	-0.14	0.02	rs16858210	A	G	0.10218	0.07336	rs16858210	G	A	-0.14	0.02	rs16858210	G	A	-0.10218	0.07336
rs16991615	G	A	-0.88	0.04	rs16991615	A	G	0.31663	0.12753	rs16991615	G	A	-0.88	0.04	rs16991615	G	A	-0.31663	0.12753
rs1713460	G	A	-0.14	0.02	rs1713460	G	A	0.07564	0.06768	rs1713460	G	A	-0.14	0.02	rs1713460	G	A	0.07564	0.06768
rs1727326	C	G	-0.19	0.03	rs1727326	C	G	0.02907	0.09142	rs1727326	C	G	-0.19	0.03	rs1727326	C	G	0.02907	0.09142
rs1799949	G	A	-0.14	0.02	rs1799949	A	G	-0.01027	0.06701	rs1799949	G	A	-0.14	0.02	rs1799949	G	A	0.01027	0.06701
rs1800932	A	G	-0.17	0.03	rs1800932	G	A	0.11622	0.07951	rs1800932	A	G	-0.17	0.03	rs1800932	A	G	-0.11622	0.07951
rs2230365	C	T	-0.17	0.03	rs2230365	T	C	0.21172	0.09036	rs2230365	C	T	-0.17	0.03	rs2230365	C	T	-0.21172	0.09036
rs2236553	C	T	-0.16	0.03	rs2236553	C	T	0.01891	0.07409	rs2236553	C	T	-0.16	0.03	rs2236553	C	T	0.01891	0.07409
rs2236918	C	G	-0.15	0.02	rs2236918	C	G	-0.17409	0.06324	rs2236918	C	G	-0.15	0.02	rs2236918	C	G	-0.17409	0.06324
rs2241584	A	G	-0.14	0.02	rs2241584	A	G	0.11333	0.06530	rs2241584	A	G	-0.14	0.02	rs2241584	A	G	0.11333	0.06530

Pre-harmonisation										Post-harmonisation									
Age natural menopause					Diastolic blood pressure					Age natural menopause					Diastolic blood pressure				
SNP	A1	A2	Beta	se	SNP	A 1	A 2	Beta	SE	SNP	a1	a2	beta	se	SNP	A 1	A2	Beta	SE
rs2277339	G	T	-0.31	0.03	rs2277339	G	T	-0.14484	0.10367	rs2277339	G	T	-0.31	0.03	rs2277339	G	T	-0.14484	0.10367
rs2547274	G	C	-0.28	0.04	rs2547274	C	G	-0.09185	0.10878	rs2547274	G	C	-0.28	0.04	rs2547274	G	C	0.09185	0.10878
rs2720044	A	C	-0.29	0.03	rs2720044	C	A	-0.00244	0.08501	rs2720044	A	C	-0.29	0.03	rs2720044	A	C	0.00244	0.08501
rs2941505	A	G	-0.13	0.02	rs2941505	A	G	-0.00580	0.06774	rs2941505	A	G	-0.13	0.02	rs2941505	A	G	-0.00580	0.06774
rs349306	G	A	-0.23	0.04	rs349306	G	A	-0.15940	0.09663	rs349306	G	A	-0.23	0.04	rs349306	G	A	-0.15940	0.09663
rs365132	G	T	-0.24	0.02	rs365132	T	G	0.11753	0.06293	rs365132	G	T	-0.24	0.02	rs365132	G	T	-0.11753	0.06293
rs3741604	T	C	-0.09	0.02	rs3741604	C	T	0.02098	0.06305	rs3741604	T	C	-0.09	0.02	rs3741604	T	C	-0.02098	0.06305
rs4246511	C	T	-0.22	0.02	rs4246511	T	C	0.13183	0.06984	rs4246511	C	T	-0.22	0.02	rs4246511	C	T	-0.13183	0.06984
rs427394	G	A	-0.13	0.02	rs427394	G	A	-0.04612	0.06388	rs427394	G	A	-0.13	0.02	rs427394	G	A	-0.04612	0.06388
rs451417	A	C	-0.2	0.03	rs451417	A	C	-0.02112	0.09641	rs451417	A	C	-0.2	0.03	rs451417	A	C	-0.02112	0.09641
rs4693089	A	G	-0.2	0.02	rs4693089	G	A	0.07425	0.06284	rs4693089	A	G	-0.2	0.02	rs4693089	A	G	-0.07425	0.06284
rs4879656	A	C	-0.12	0.02	rs4879656	A	C	0.09534	0.06486	rs4879656	A	C	-0.12	0.02	rs4879656	A	C	0.09534	0.06486
rs4886238	G	A	-0.18	0.02	Excluded QC					rs4886238	G	A	-0.18	0.02	rs4886238	Excluded QC			
rs551087	G	A	-0.13	0.02	rs551087	G	A	0.11758	0.06954	rs551087	G	A	-0.13	0.02	rs551087	G	A	0.11758	0.06954
rs5762534	T	C	-0.16	0.03	rs5762534	C	T	0.32279	0.08848	rs5762534	T	C	-0.16	0.03	rs5762534	T	C	-0.32279	0.08848
rs6484478	G	A	-0.1	0.02	rs6484478	A	G	-0.04828	0.07476	rs6484478	G	A	-0.1	0.02	rs6484478	G	A	0.04828	0.07476
rs6856693	A	G	-0.16	0.02	rs6856693	G	A	-0.12295	0.06349	rs6856693	A	G	-0.16	0.02	rs6856693	A	G	0.12295	0.06349
rs6899676	A	G	-0.23	0.03	rs6899676	G	A	-0.03488	0.07864	rs6899676	A	G	-0.23	0.03	rs6899676	A	G	0.03488	0.07864
rs704795	A	G	-0.16	0.02	rs704795	A	G	-0.20850	0.06420	rs704795	A	G	-0.16	0.02	rs704795	A	G	-0.20850	0.06420
rs707938	G	A	-0.17	0.02	rs707938	G	A	-0.31493	0.06731	rs707938	G	A	-0.17	0.02	rs707938	G	A	-0.31493	0.06731
rs7259376	A	G	-0.11	0.02	rs7259376	A	G	-0.10956	0.06302	rs7259376	A	G	-0.11	0.02	rs7259376	A	G	-0.10956	0.06302
rs7397861	G	C	-0.1	0.02	rs7397861	C	G	0.04052	0.06515	rs7397861	G	C	-0.1	0.02	rs7397861	G	C	-0.04052	0.06515
rs763121	G	A	-0.16	0.02	rs763121	G	A	-0.04170	0.06622	rs763121	G	A	-0.16	0.02	rs763121	G	A	-0.04170	0.06622
rs8070740	A	G	-0.15	0.02	rs8070740	G	A	-0.01549	0.07347	rs8070740	A	G	-0.15	0.02	rs8070740	A	G	0.01549	0.07347
rs9039	C	T	-0.12	0.02	rs9039	C	T	0.02521	0.07082	rs9039	C	T	-0.12	0.02	rs9039	C	T	0.02521	0.07082
rs930036	A	G	-0.19	0.02	rs930036	A	G	0.22466	0.06460	rs930036	A	G	-0.19	0.02	rs930036	A	G	0.22466	0.06460
rs9393800	G	A	-0.17	0.02	rs9393800	G	A	-0.11434	0.07155	rs9393800	G	A	-0.17	0.02	rs9393800	G	A	-0.11434	0.07155
rs9796	T	A	-0.13	0.02	rs9796	T	A	0.20068	0.06301	rs9796	T	A	-0.13	0.02	rs9796	T	A	0.20068	0.06301

TABLE S5. OBSERVATIONAL ASSOCIATION BETWEEN AGE OF NATURAL MENOPAUSE AND BLOOD PRESSURE TRAITS

ANM	Model 1		Model 2		Model 3		p trend
	β(95% CI)	p	β(95% CI)	p	β(95% CI)	p	
Systolic Blood Pressure							
CoLaus							
Continuous	0.32 (0.07;0.56)	0.013	0.01(-0.23;0.26)	0.921	-0.01(-0.25;0.23)	0.939	
Categories							
Early	-2.71(-6.39;0.97)	0.149	0.20(-3.35;3.75)	0.913	0.17(-3.36;3.71)	0.923	0.533
Intermediate	-2.70(-5.22;-0.18)	0.036	-0.99(-3.41;1.43)	0.422	-0.74(-3.17;1.67)	0.545	
Normal	Ref	Ref	Ref	Ref	Ref	Ref	
Late	2.76(-0.32;5.85)	0.079	0.88(-2.09;3.84)	0.561	0.86(-2.09;3.82)	0.566	
RS-I-3							
Continuous	0.12(-0.14;0.38)	0.378	0.18(-0.08;0.44)	0.164	0.18(-0.08;0.44)	0.179	
Categories							
Early	-0.66(-4.31;2.99)	0.722	-1.81(-5.43;1.81)	0.327	-1.73(-5.35;1.89)	0.349	0.383
Intermediate	-0.41(-2.89;2.06)	0.743	-0.67(-3.12;1.77)	0.590	-0.58(-3.03;1.86)	0.639	
Normal	Ref	Ref	Ref	Ref	Ref	Ref	
Late	0.83(-2.69;4.34)	0.644	0.34(-3.14;3.81)	0.849	0.53(-2.94;4.01)	0.763	
RS-II-1							
Continuous	-0.19(-0.55;0.17)	0.296	-0.12(-0.45;0.21)	0.484	-0.07(-0.41;0.27)	0.687	
Categories							
Early	-1.66(-6.94;3.62)	0.537	-2.17(-7.07;2.72)	0.384	-2.79(-7.76;2.18)	0.272	0.556
Intermediate	3.51(-0.11;7.13)	0.058	2.70(-0.66;6.06)	0.115	2.62(-0.75;5.99)	0.128	
Normal	Ref	Ref	Ref	Ref	Ref	Ref	
Late	-1.83(-5.81;2.16)	0.369	-1.82(-5.51;1.87)	0.333	-1.61(-5.32;2.10)	0.395	
RS-III-1							
Continuous	0.67(0.33;1.01)	<0.001	0.52(0.19;0.85)	0.002	0.52(0.18;0.86)	0.003	
Categories							
Early	-6.60(-11.78;-1.43)	0.012	-5.89(-10.92;-0.86)	0.022	-5.69(-10.76;-0.63)	0.028	0.01
Intermediate	-4.55(-7.71;-1.39)	0.005	-3.37(-6.46;-0.29)	0.032	-3.28(-6.38;-0.18)	0.038	
Normal	Ref	Ref	Ref	Ref	Ref	Ref	
Late	1.97(-1.83;5.77)	0.309	0.88(-2.83;4.59)	0.641	0.98(-2.74;4.70)	0.606	
Diastolic Blood Pressure							

ANM	Model 1		Model 2		Model 3		p trend
	β(95% CI)	p	β(95% CI)	p	β(95% CI)	p	
CoLaus							
Continuous	0.1(-0.04;0.24)	0.148	0.16(0.01;0.30)	0.031	0.14(-0.04;0.28)	0.058	
Categories							
Early	-2.21(-4.30;-0.11)	0.039	-2.67(-4.77;-0.57)	0.013	-2.66(-4.75;-0.57)	0.013	0.04
Intermediate	-0.70(-2.13;0.73)	0.337	-0.82(-2.26;0.61)	0.261	-0.82(-2.26;0.61)	0.259	
Normal	Ref	Ref	Ref	Ref	Ref	Ref	
Late	-0.35(-2.11;1.40)	0.693	0.03(-1.78;1.72)	0.973	-0.15(-1.89;1.60)	0.868	
RS-I-3							
Continuous	0.00(-0.14;0.13)	0.957	-0.02(-0.16;0.11)	0.74	-0.03(-0.17;0.11)	0.674	
Categories							
Early	0.09(-1.80;1.98)	0.923	0.43(-1.46;2.32)	0.655	0.48(-1.42;2.37)	0.621	0.364
Intermediate	0.63(-0.65;1.91)	0.337	0.70(-0.57;1.98)	0.28	0.73(-0.55;2.00)	0.265	
Normal	Ref	Ref	Ref	Ref	Ref	Ref	
Late	0.21(-1.61;2.03)	0.824	0.35(-1.46;2.16)	0.704	0.32(-1.50;2.13)	0.734	
RS-II-1							
Continuous	-0.01(-0.19;0.17)	0.883	-0.02(-0.20;0.16)	0.854	-0.03(-0.22;0.15)	0.737	
Categories							
Early	-1.19(-3.84;1.47)	0.380	-1.16(-3.82;1.49)	0.390	-1.03(-3.72;1.67)	0.454	0.757
Intermediate	0.25(-1.57;2.07)	0.791	0.28(-1.54;2.11)	0.760	0.31(-1.52;2.14)	0.739	
Normal	Ref	Ref	Ref	Ref	Ref	Ref	
Late	-0.76(-2.76;1.24)	0.457	-0.76(-2.76;1.24)	0.457	-0.84(-2.85;1.18)	0.415	
RS-III-1							
Continuous	0.28(0.09;0.48)	0.004	0.27(0.08;0.46)	0.006	0.24(0.05;0.44)	0.014	
Categories							
Early	-3.64(-6.53;-0.74)	0.014	-3.56(-6.46;-0.66)	0.016	-3.30(-6.21;-0.38)	0.027	0.053
Intermediate	-0.76(-2.53;1.01)	0.399	-0.63(-2.41;1.14)	0.484	-0.51(-2.30;1.27)	0.572	
Normal	Ref	Ref	Ref	Ref	Ref	Ref	
Late	1.47(-0.66;3.60)	0.175	1.35(-0.78;3.49)	0.213	1.27(-0.88;3.41)	0.246	
			Hypertension				
CoLaus							
Continuous	1.04(1.01;1.07)	0.006	1.01(0.98;1.04)	0.38	1.01(0.98;1.04)	0.477	

ANM	Model 1		Model 2		Model 3		p trend
	β(95% CI)	p	β(95% CI)	p	β(95% CI)	p	
Categories							
Early	0.64(0.42;0.98)	0.039	0.81(0.52;1.27)	0.358	0.80(0.51;1.25)	0.327	0.181
Intermediate	0.80(0.61;1.07)	0.133	0.94(0.70;1.26)	0.667	0.95(0.71;1.29)	0.76	
Normal	Ref	Ref	Ref	Ref	Ref	Ref	
Late	1.26(0.89;1.77)	0.193	1.06(0.75;1.51)	0.741	1.02(0.74;1.50)	0.898	
RS-I-3							
Continuous	1.01(0.98;1.04)	0.555	1.02(0.99;1.05)	0.166	1.02(0.99;1.05)	0.148	
Categories							
Early	1.19(0.81;1.74)	0.380	0.97(0.65;1.44)	0.260	0.96(0.65;1.43)	0.849	0.551
Intermediate	1.08(0.84;1.39)	0.549	1.04(0.80;1.34)	0.860	1.04(0.80;1.35)	0.786	
Normal	Ref	Ref	Ref	Ref	Ref	Ref	
Late	1.30(0.89;1.89)	0.17	1.18(0.81;1.73)	0.792	1.24(0.84;1.82)	0.282	
RS-II-1							
Continuous	1.01(0.97;1.04)	0.667	1.02(0.98;1.05)	0.425	1.02(0.98;1.06)	0.317	
Categories							
Early	1.05(0.62;1.77)	0.866	0.96(0.55;1.68)	0.889	0.91(0.51;1.62)	0.751	0.866
Intermediate	1.24(0.86;1.80)	0.248	1.16(0.79;1.71)	0.453	1.15(0.78;1.70)	0.488	
Normal	Ref	Ref	Ref	Ref	Ref	Ref	
Late	1.09(0.73;1.63)	0.665	1.06(0.70;1.61)	0.792	1.06(0.69;1.62)	0.796	
RS-III-1							
Continuous	1.03(1.00;1.07)	0.064	1.02(0.98;1.06)	0.273	1.02(0.98;1.06)	0.271	
Categories							
Early	0.81(0.48;1.38)	0.437	0.85(0.49;1.46)	0.553	0.88(0.51;1.52)	0.637	0.062
Intermediate	0.98(0.71;1.34)	0.877	1.08(0.78;1.50)	0.640	1.09(0.78;1.52)	0.628	
Normal	Ref	Ref	Ref	Ref	Ref	Ref	
Late	1.70(1.15;2.51)	0.008	1.54(1.03;2.29)	0.034	1.58(1.06;2.37)	0.026	

OR= Odds ratio; CI=Confidence interval

Model 1: Antihypertensive medication.
Model 2: Antihypertensive medication, age.
Model 3: Antihypertensive medication, age, smoking status, drinking status, education level
Colaus (n=1139), RS-I-3 (n=1603), RS-II-1 (n=790), RS-III-1 (n= 919)

TABLE S6. P VALUES FOR INTERACTION BETWEEN AGE OF NATURAL MENOPAUSE AND BODY MASS INDEX

	SBP	DBP*	Hypertension
CoLaus	0.108	0.079	0.054
RS-I-3	0.430	0.482	0.101
RS-II-1	0.269	0.079	0.205
RS-III-1	0.564	0.841	0.549

Results based on the fully adjusted model (Model 4). SBP=systolic blood pressure, DBP=diastolic blood pressure, * 3 Observations deleted

TABLE S7. COMPARISON OF ESTIMATES ROTTERDAM STUDY IMPUTED VS WITHOUT IMPUTATION

Cohort	RS-I-3				RS-II-1				RS-III-1			
	Imputed n=1,603		Without imputation n=1,330		Imputed n=790		Without imputation n=265		Imputed n=919		Without imputation n=676	
Trait	Systolic Blood Pressure											
ANM	β (95% CI)	p	β (95% CI)	p	β(95% CI)	p	β (95% CI)	p	β (95% CI)	p	β (95% CI)	p
Continuous	0.15(-0.11;0.40)	0.272	0.28(-0.01;0.56)	0.055	-0.05(-0.38;0.28)	0.769	0.28(-0.26;0.83)	0.305	0.45(0.11;0.78)	0.010	0.31(-0.08;0.69)	0.119
Categories												
Early	-1.83(-5.43;1.77)	0.318	-3.88(-7.79;0.04)	0.052	-2.86(-7.76;2.03)	0.251	-6.15(-13.52;1.22)	0.102	-5.51(-10.50;-0.52)	0.031	-5.17(-10.83;0.48)	0.073
Intermediate	-0.59(-3.02;1.84)	0.318	-1.05(-3.72;1.62)	0.442	2.30(-1.01;5.61)	0.174	0.11(-5.32;5.54)	0.968	-2.88(-5.95;0.19)	0.066	-2.39(-5.96;1.18)	0.189
Normal	Ref											
Late	-0.21(-3.67;3.26)	0.906	-0.83(-4.59;2.93)	0.664	-1.68(-5.33;1.97)	0.367	-0.94(-7.05;5.16)	0.761	0.57(-3.10;4.24)	0.761	-1.17(-5.30;2.96)	0.579
Trait	Diastolic Blood Pressure											
Continuous	-0.05(-0.18;0.09)	0.511	0.00(-0.15;0.14)	0.953	-0.05(-0.23;0.14)	0.619	-0.04(-0.34;0.25)	0.767	0.17(-0.02;0.36)	0.082	0.09(-0.12;0.31)	0.408
Categories												
Early	0.45(-1.43;2.32)	0.641	-0.12(-2.20;1.95)	0.906	-1.13(-3.78;1.53)	0.405	0.74(-3.31;4.78)	0.720	-2.99(-5.80;-0.19)	0.036	-2.28(-5.42;0.86)	0.155
Intermediate	0.72(-0.55;1.99)	0.268	0.79(-0.62;2.20)	0.272	0.34(-1.45;2.14)	0.709	0.44(-2.53;3.42)	0.769	-0.05(-1.78;1.67)	0.951	0.19(-1.80;2.17)	0.855
Normal	Ref											
Late	-0.02(-1.83;1.79)	0.983	-0.02(-2.01;1.97)	0.988	-0.96(-2.95;1.02)	0.342	0.03(-3.32;3.38)	0.986	0.93(-1.13;2.99)	0.378	0.09(-2.20;2.39)	0.936
Trait	Hypertension											
	n=1,603		n=1,455		n=790		n=315		n=919		n=763	
ANM	OR(95% CI)	p	OR(95% CI)	p	OR(95% CI)	p	OR (95% CI)	p	OR (95% CI)	p	OR (95% CI)	p
Continuous	1.02(0.99;1.05)	0.152	1.03(1.00;1.07)	0.068	1.03(0.99;1.07)	0.198	1.06(0.98;1.14)	0.134	1.03(0.99;1.07)	0.156	1.03(0.98;1.08)	0.195
Categories												
Early	0.92(0.61;1.39)	0.693	0.74(0.47;1.16)	0.185	0.91(0.50;1.67)	0.772	0.68(0.26;1.81)	0.442	0.76(0.43;1.35)	0.346	0.68(0.35;1.33)	0.264
Intermediate	1.04(0.79;1.37)	0.780	1.03(0.76;1.40)	0.863	1.05(0.69;1.59)	0.819	0.92(0.44;1.95)	0.838	0.99(0.69;1.41)	0.945	0.89(0.58;1.36)	0.589
Normal	Ref											
Late	1.17(0.79;1.75)	0.429	1.15(0.74;1.79)	0.535	1.03(0.66;1.62)	0.891	1.42(0.61;3.26)	0.414	1.50(0.98;2.29)	0.061	1.31(0.80;2.12)	0.283

OR= Odds ratio; CI=Confidence interval

Models adjusted for age and glomerular filtration rate, body mass index, total cholesterol, drinking status, education level, smoking status, prevalent Type 2 diabetes, history of cardiovascular diseases, statin use, hormone replacement therapy, antihypertensive medication.

TABLE S8. SENSITIVITY ANALYSIS OBSERVATIONAL ESTIMATES OF THE ASSOCIATION BETWEEN CATEGORIES OF ANM AND BP TRAITS

Patients excluded	Hormone Therapy	>65 years	History of CVD	>10 years menopause	Hypertension	HTA defined as use of antihypertensive medication	HTA defined as use of antihypertensive medication & eGFR <60 ml/min/1.73m2
Outcomes	β (95% CI)	β (95% CI)	β (95% CI)	β (95% CI)	β (95% CI)	β (95% CI)	β (95% CI)
Systolic BP							
Early	-2.95(-5.28;-0.62)	-1.35(-4.10;1.39)	-1.53(-3.67;0.61)	0.03(-0.94;0.99)	-0.94(-2.65;0.76)	-	-
Intermediate	-0.26(-1.80;1.29)	-1.06(-2.84;0.73)	-0.44(-1.86;0.98)	-0.90(-3.71;1.91)	-0.56(-1.71;0.60)	-	-
Normal	Ref	Ref	Ref	Ref	Ref		
Late	0.50(-1.45;2.45)	-1.04(-3.22;1.14)	-0.31(-2.06;1.44)	-0.88(-3.32;1.57)	-1.24(-2.77;0.28)	-	-
Diastolic BP							
Early	-1.29(-2.56;-0.02)	-2.07(-3.65;-0.50)	-1.44(-2.63;-0.26)	-2.03(-8.28;4.21)	-1.23(-2.44;-0.02)	-	-
Intermediate	0.32(-0.53;1.16)	-0-22(-1.24;0.79)	0.17(-0.62;0.95)	-0.22(-1.87;1.42)	-0.14(-0.96;0.68)	-	-
Normal	Ref	Ref	Ref	Ref	Ref		
Late	0.16(-0.91;1.23)	-0.35(-1.60;0.89)	0.03(-0.94;0.19)	-0.35(-1.75;1.05)	-0.56(-1.64;0.53)	-	-
Hypertension	OR(95% CI)	OR(95% CI)	OR(95% CI)	OR(95% CI)	OR(95% CI)	OR(95% CI)	OR(95% CI)
Early	0.71(0.53;0.94)	0.83(0.58;1.17)	0.82(0.63;1.06)	0.47(0.17;1.28)	-	1.08(0.83;1.41)	1.09(0.81;1.46)
Intermediate	1.01(0.84;1.21)	0.98(0.78;1.23)	1.02(0.86;1.21)	0.95(0.64;1.39)	-	0.99(0.83;1.18)	1.01(0.83;1.24)
Normal	Ref	Ref	Ref	Ref		Ref	Ref
Late	1.22(0.97;1.55)	1.14(0.87;1.49)	1.15(0.94;1.42)	1.31(0.96;1.79)	-	1.28(1.03;1.58)	1.26(0.10;1.59)

OR= Odds ratio; CI=Confidence interval

Patients excluded= Patients with the characteristic e.g., hormone therapy use etc. were excluded to perform the sensitivity analysis

Fixed effects meta-analysis.

TABLE S9. ASSOCIATION GENETIC RISK SCORE OF AGE OF NATURAL MENOPAUSE (ANM) AND OBSERVED ANM

	n	p Value	R-squared	F *
CoLaus	1137	<0.001	0.0337	40.63
RS-I-3	1603	<0.001	0.0146	23.67
RS-II-1	790	0.0009	0.0140	11.15
RS-III-1	919	0.0000	0.0199	18.60

* First-stage regressions

TABLE S10. ASSOCIATION OF WEIGHTED GENETIC RISK SCORE WITH BLOOD PRESSURE TRAITS BY STUDY.

GRS-ANM	Model 1		Model 2*		Model 1		Model 2*		Model 1		Model 2*	
	Systolic blood pressure				Diastolic blood pressure				Hypertension*			
	β (95% CI)	p	β (95% CI)	p	β (95% CI)	p	β (95% CI)	p	OR (95% CI)	p	OR (95% CI)	p
CoLaus (n=1139)												
Cont.	-0.05 (-0.30;0.21)	0.716	-0.01 (-0.26;0.24)	0.937	-0.03 (-0.18;0.12)	0.675	-0.01 (-0.15;0.14)	0.919	0.99 (0.96;1.02)	0.581	0.99 (0.96;1.03)	0.752
Q5												
1	Ref				Ref				Ref			
2	0.16 (-3.07;3.40)	0.922	0.05 (-3.09;3.19)	0.975	-0.56 (-2.47;1.36)	0.568	-0.60 (-2.43;1.23)	0.521	1.05 (0.71;1.54)	0.808	1.00 (0.67;1.50)	0.995
3	0.58 (-2.65;3.81)	0.726	-0.08 (-3.22;3.07)	0.960	0.58 (-1.33;2.49)	0.550	0.13 (-1.71;1.96)	0.892	1.16 (0.79;1.69)	0.453	1.05 (0.70;1.57)	0.815
4	-1.20 (-4.43;2.04)	0.468	-0.75 (-3.91;2.41)	0.642	-0.86 (-2.77;1.05)	0.377	-0.63 (-2.47;1.21)	0.504	0.88 (0.60;1.30)	0.520	0.84 (0.55;1.27)	0.399
5	0.08 (-3.15;3.31)	0.961	0.24 (-2.91;3.39)	0.882	-0.24 (-2.15;1.67)	0.806	-0.19 (-2.02;1.65)	0.841	0.97 (0.66;1.43)	0.894	0.97 (0.64;1.46)	0.875
RS-I-3 (n= 1603)												
Cont.	-0.20 (-0.44;0.03)	0.082	-0.20 (-0.43;0.03)	0.086	-0.06 (-0.18;0.06)	0.338	-0.06 (-0.18;0.06)	0.325	0.99 (0.97;1.01)	0.401	0.99 (0.97;1.02)	0.639
Q5												
1	Ref				Ref				Ref			
2	2.06 (-1.15;5.27)	0.209	2.00 (-1.16;5.17)	0.215	0.78 (-0.88;2.45)	0.357	0.72 (-0.93;2.37)	0.393	1.07 (0.75;1.51)	0.715	1.14 (0.79;1.63)	0.487
3	-1.34 (-4.52;1.83)	0.407	-1.26 (-4.40;1.88)	0.431	-0.28 (-1.93;1.37)	0.742	-0.31 (-1.95;1.33)	0.709	0.82 (0.59;1.15)	0.249	0.84 (0.59;1.19)	0.318
4	-1.30 (-4.54;1.94)	0.432	-1.41 (-4.62;1.79)	0.388	-0.95 (-2.63;0.73)	0.268	-0.95 (-2.63;0.72)	0.263	0.97 (0.68;1.38)	0.873	0.99 (0.69;1.42)	0.955
5	-2.27 (-5.45;0.92)	0.163	-2.21 (-5.35;0.94)	0.169	-0.63 (-2.28;1.02)	0.454	-0.71 (-2.36;0.93)	0.394	0.89 (0.64;1.25)	0.509	0.95 (0.67;1.35)	0.786
RS-II-1 (n= 790)												
Cont.	-0.06 (-0.36;0.25)	0.715	-0.07 (-0.37;0.22)	0.614	0.05 (-0.11;0.21)	0.529	0.03 (-0.13;0.19)	0.71	1.00 (0.97;1.04)	0.935	1.00 (0.97;1.04)	0.973
Q5												
1	Ref				Ref				Ref			
2	1.11 (-3.20;5.43)	0.613	0.74 (-3.41;4.88)	0.727	1.28 (-1.00;3.56)	0.271	0.83 (-1.42;3.08)	0.470	0.91 (0.57;1.47)	0.701	0.91 (0.55;1.52)	0.725
3	-1.30 (-5.87;3.26)	0.576	-1.23 (-5.60;3.14)	0.582	0.44 (-1.98;2.85)	0.723	0.18 (-2.19;2.56)	0.879	0.75 (0.46;1.25)	0.272	0.73 (0.42;1.25)	0.247
4	-0.42 (-4.72;3.89)	0.850	-1.49 (-5.63;2.65)	0.481	0.53 (-1.74;2.81)	0.646	-0.01 (-2.26;2.23)	0.993	0.87 (0.54;1.40)	0.553	0.86 (0.51;1.44)	0.563
5	-0.81 (-5.20;3.58)	0.716	-0.78 (-4.98;3.43)	0.717	0.72 (-1.61;3.04)	0.545	0.40 (-1.88;2.68)	0.733	0.98 (0.60;1.60)	0.941	1.01 (0.60;1.71)	0.960
RS-III-1 (n=919)												
Cont.	0.06 (-0.22;0.35)	0.655	0.05 (-0.23;0.33)	0.735	0.04 (-0.12;0.20)	0.617	0.01 (-0.14;0.17)	0.863	0.99 (0.96;1.02)	0.655	0.99 (0.96;1.02)	0.574

GRS-ANM	Model 1		Model 2*		Model 1		Model 2*		Model 1		Model 2*	
	Systolic blood pressure				Diastolic blood pressure				Hypertension*			
Q5												
1	Ref				Ref				Ref			
2	-0.43 (-4.49;3.64)	0.837	-0.20 (-4.21;3.80)	0.920	0.11 (-2.23;2.45)	0.925	0.52 (-1.73;2.76)	0.651	0.86 (0.56;1.32)	0.483	0.88 (0.55;1.39)	0.579
3	-2.41 (-6.37;1.55)	0.233	-2.31 (-6.20;1.58)	0.244	0.19 (-2.09;2.47)	0.870	0.21 (-1.97;2.39)	0.853	0.76 (0.50;1.16)	0.199	0.76 (0.49;1.19)	0.228
4	1.55 (-2.47;5.57)	0.449	1.52 (-2.43;5.47)	0.451	2.34 (0.03;4.65)	0.047	2.29 (0.08;4.51)	0.042	1.10 (0.72;1.68)	0.659	1.07 (0.68;1.68)	0.780
5	0.50 (-3.57;4.57)	0.809	0.52 (-3.47;4.52)	0.797	0.65 (-1.69;2.99)	0.585	0.53 (-1.70;2.77)	0.639	0.89 (0.58;1.36)	0.582	0.92 (0.58;1.45)	0.711

Model 1: Adjusted for age, glomerular filtration rate.
Model 2: Adjusted for age and glomerular filtration rate, body mass index, total cholesterol, drinking status, education level, smoking status, prevalent Type 2 diabetes, history of cardiovascular diseases, statin use, hormone replacement therapy, use of antihypertensive medication.
*(n=1137) Observations
OR= Odds ratio; CI=Confidence interval; p=p value; GRS-ANM = Genetic Risk Score of Age of menopause; Cont.=Continuous; Q5=Quintiles
Reference category was lowest quintile
* Not adjusted for antihypertensive medication

TABLE S11. ASSOCIATION OF THE GENETIC RISK SCORE WITH CONFOUNDERS BY STUDY

	CoLaus				RS-I-3				RS-II-1				RS-III-1				Meta-analysis	
	Crude		Adjusted		Crude		Adjusted		Crude		Adjusted		Crude		Adjusted		Crude	Adjusted
Variable	β (95% CI)	p	β (95% CI)	p	β (95% CI)	p	β (95% CI)	p	β (95% CI)	p	β (95% CI)	p	β (95% CI)	p	β (95% CI)	p	β (95% CI)	β (95% CI)
Age	-0.12 (-0.22;-0.01)	0.03	-0.10 (-0.19;-0.00)	0.04	0.01 (-0.07;0.10)	0.78	0.03 (-0.04;0.10)	0.39	0.12 (-0.01;0.25)	0.07	0.10 (0.00;0.21)	0.05	0.07 (-0.03;0.16)	0.18	0.07 (-0.02;0.16)	0.11	0.02 (-0.08; 0.11)	0.02 (-0.06; 0.10)
Glomerular filtration rate	0.08 (-0.12;0.28)	0.44	-0.01 (-0.19;0.17)	0.92	0.07 (-0.10;0.23)	0.44	0.08 (-0.06;0.22)	0.28	-0.04 (-0.28;0.19)	0.71	0.08 (-0.11;0.27)	0.40	0.03 (-0.17;0.23)	0.78	0.09 (-0.09;0.26)	0.35	0.04 (-0.05; 0.14)	0.06 (-0.02; 0.15)
Body mass index	-0.01 (-0.08;0.06)	0.82	-0.00 (-0.07;0.07)	0.92	-0.04 (-0.09;0.01)	0.10	-0.04 (-0.09;0.01)	0.13	0.03 (-0.04;0.09)	0.44	0.03 (-0.04;0.10)	0.37	0.02 (-0.05;0.09)	0.50	0.03 (-0.04;0.09)	0.47	-0.01 (-0.04; 0.03)	-0.0003 (-0.04; 0.04)
Total cholesterol	0.00 (-0.01;0.01)	0.93	0.00 (-0.01;0.01)	0.75	0.00 (-0.01;0.01)	0.98	0.00 (-0.01;0.01)	0.98	-0.01 (-0.02;0.01)	0.39	-0.01 (-0.02;0.01)	0.45	0.00 (-0.01;0.02)	0.93	0.00 (-0.01;0.02)	0.94	-0.002 (-0.01; 0.00)	-0.002 (-0.02; 0.004)
Statin use	0.95 (0.91;1.00)	0.06	0.97 (0.92;1.00)	0.11	-0.02 (-0.06;0.01)	0.21	-0.02 (-0.06;0.01)	0.24	0.00 (-0.05;0.04)	0.86	-0.01 (-0.05;0.04)	0.78	0.00 (-0.04;0.03)	0.85	0.00 (-0.04;0.03)	0.79	0.23 (-0.19; 0.66)	0.24 (-0.23; 0.70)
*Hormone replacement therapy	1.03 (1.00;1.06)	0.01	1.04 (1.01;1.07)	0.01	1.03 (0.96;1.12)	0.37	1.03 (0.96;1.11)	0.39	1.04 (0.96;1.13)	0.30	1.05 (0.96;1.13)	0.28	0.90 (0.80;1.02)	0.09	0.91 (0.81;1.02)	0.11	1.02 (0.98;1.06)	1.02 (0.98;1.07)
*Antihypertensive medication	0.98 (0.94;1.00)	0.15	0.98 (0.94;1.02)	0.28	1.00 (0.97;1.02)	0.82	1.00 (0.98;1.02)	0.91	1.01 (0.98;1.05)	0.47	1.01 (0.97;1.04)	0.69	1.01 (0.98;1.05)	0.40	1.01 (0.98;1.05)	0.44	0.10 (0.98;1.01)	1.00 (0.99; 1.02)
*Drinking status	0.99 (0.96;1.03)	0.74	0.99 (0.97;1.02)	0.76	0.99 (0.97;1.02)	0.49	0.99 (0.97;1.02)	0.53	1.01 (0.97;1.05)	0.56	1.01 (0.97;1.06)	0.50	1.06 (0.97;1.15)	0.18	1.07 (0.98;1.16)	0.14	0.10 (0.98;1.02)	0.98 (0.98;1.02)
*Diabetes mellitus, type 2	0.95 (0.88;1.02)	0.21	0.96 (0.89;1.02)	0.24	0.98 (0.95;1.01)	0.19	0.98 (0.94;1.01)	0.17	1.00 (0.95;1.05)	0.96	1.00 (0.95;1.05)	0.94	0.98 (0.93;1.04)	0.57	0.98 (0.92;1.04)	0.50	0.98 (0.92;1.00)	0.98 (0.92 ;1.01)
*History of CVD	1.01 (0.96;1.07)	0.50	1.02 (0.97;1.08)	0.33	0.98 (0.94;1.01)	0.24	0.98 (0.94;1.01)	0.22	1.01 (0.94;1.08)	0.81	0.99 (0.92;1.07)	0.86	1.04 (0.95;1.14)	0.37	1.04 (0.95;1.13)	0.38	0.10 (0.97;1.02)	0.10 (0.97; 1.02)
*Smoking status	1.03 (1.00;1.05)	0.06	1.02 (0.99;1.05)	0.1	0.96 (0.93;0.99)	0.02	0.96 (0.93;0.99)	0.02	0.97 (0.94;1.01)	0.18	0.98 (0.94;1.02)	0.27	1.01 (0.97;1.04)	0.67	1.01 (0.98;1.05)	0.50	0.99 (0.94;1.03)	0.99 (0.96;1.02)
**Education level	0.99 (0.96;1.02)	0.88	1.00 (0.97;1.03)	0.8	1.00 (0.98;1.03)	0.90	1.00 (0.98;1.03)	0.86	1.03 (1.00;1.07)	0.08	1.03 (1.00;1.07)	0.06	1.00 (0.97;1.03)	0.93	1.00 (0.97;1.03)	0.80	1.00 (0.99; 1.02)	1.01 (0.99;1.02)

OR = Odds ratio; CI=Confidence interval; p=p value

* Logistic regression, OR

** Ordinal logistic regression OR

Adjusted by age, eGFR.

Statistically significant p-value (Bonferroni correction) ≤ 0.0041

TABLE S12. ASSOCIATION OF EACH SNP INCLUDED IN THE GENETIC RISK SCORE WITH CONFOUNDERS BY STUDY

		CoLaus (n=1141)																									
		Age		Smoking status		Alcohol drinker		Educat onal level		eGFR		BMI		Cardiovascular disease history		Total cholesterol		Diabetes		Stat ns therapy		HRT		Receiving BP-related medicat on	
n	SNP	β	p	OR	p	OR	p	OR	p	β	p	β	p	OR	p	β	p	OR	p	OR	p	OR	p	OR	p
1	rs4246511	-0.410	0.224	1.062	0.529	0.852	0.112	1.084	0.406	0.003	0.997	-0.068	0.773	0.810	0.232	-0.063	0.184	0.943	0.786	0.929	0.607	1.162	0.116	0.921	0.462
2	rs12142240	-0.587	0.077	0.945	0.548	1.002	0.985	0.972	0.768	0.696	0.279	0.008	0.972	0.980	0.908	0.073	0.114	1.008	0.972	0.804	0.14	0.867	0.132	0.847	0.144
3	rs1411478	0.267	0.384	0.923	0.354	1.085	0.368	0.939	0.481	-0.620	0.295	0.136	0.522	1.129	0.466	0.033	0.442	1.083	0.692	0.881	0.33	0.861	0.086	1.030	0.776
4	rs2236918	0.103	0.734	0.984	0.851	0.977	0.796	0.985	0.865	1.118	0.057	-0.119	0.573	0.853	0.328	0.089	0.037	0.773	0.194	0.919	0.516	0.926	0.372	1.094	0.379
5	rs704795	-0.405	0.194	1.185	0.054	0.935	0.463	0.968	0.718	-1.369	0.023	-0.032	0.882	0.901	0.539	-0.021	0.638	1.083	0.694	1.040	0.767	1.005	0.957	0.888	0.261
6	rs1800932	-0.146	0.685	1.009	0.927	0.868	0.175	0.948	0.604	-0.613	0.377	-0.110	0.657	0.714	0.114	-0.051	0.305	1.043	0.856	0.880	0.419	1.193	0.086	0.869	0.254
7	rs930036	-0.198	0.499	0.916	0.286	1.053	0.551	1.053	0.544	0.450	0.426	0.055	0.785	1.166	0.321	0.112	0.006	0.979	0.913	0.881	0.32	1.054	0.528	0.925	0.425
8	rs16858210	-0.275	0.42	0.975	0.791	1.119	0.268	0.962	0.692	0.571	0.387	0.267	0.259	1.168	0.384	-0.014	0.77	1.468	0.067	1.140	0.359	0.924	0.411	1.110	0.356
9	rs4693089	0.519	0.088	1.114	0.208	0.986	0.876	1.089	0.33	-0.837	0.155	-0.056	0.79	0.859	0.356	-0.004	0.93	0.816	0.312	1.192	0.179	1.001	0.99	0.976	0.811
10	rs6856693	0.150	0.622	1.020	0.821	1.086	0.356	0.929	0.405	0.163	0.782	0.021	0.921	1.057	0.733	-0.021	0.629	0.791	0.248	0.819	0.132	0.964	0.67	0.920	0.411
11	rs427394	0.173	0.573	0.919	0.33	0.911	0.305	1.010	0.914	-0.061	0.917	-0.033	0.876	1.024	0.885	-0.011	0.805	0.774	0.215	0.977	0.86	0.988	0.89	1.116	0.281
12	rs11738223	0.448	0.163	1.153	0.117	1.038	0.698	1.044	0.643	-0.194	0.755	0.293	0.189	1.120	0.504	-0.043	0.337	1.140	0.525	1.410	0.01	1.235	0.022	1.166	0.149
13	rs2241584	-0.351	0.254	1.194	0.042	0.947	0.548	0.987	0.881	-0.107	0.858	-0.137	0.521	0.848	0.328	0.096	0.026	0.897	0.591	0.896	0.411	1.057	0.527	0.965	0.731
14	rs365132	0.176	0.561	0.975	0.771	0.989	0.899	0.955	0.599	-0.405	0.49	0.395	0.06	1.032	0.846	0.002	0.961	1.276	0.221	1.111	0.42	0.757	0.001	1.276	0.017
15	rs6899676	-0.445	0.249	0.959	0.698	0.974	0.814	0.918	0.439	-0.307	0.681	-0.499	0.062	0.702	0.12	-0.045	0.404	0.848	0.531	0.959	0.804	1.082	0.475	1.058	0.661
16	rs9393800	-0.657	0.063	1.043	0.676	1.180	0.118	1.001	0.991	0.858	0.209	0.239	0.33	0.969	0.867	-0.008	0.873	0.774	0.294	0.768	0.097	1.103	0.33	0.792	0.056
17	rs2230365	-0.205	0.622	0.921	0.482	0.892	0.346	1.210	0.121	-0.836	0.299	-0.260	0.366	0.989	0.961	0.010	0.863	0.962	0.888	1.205	0.273	0.913	0.441	1.073	0.606
18	rs707938	-0.622	0.055	1.069	0.462	1.002	0.985	1.129	0.2	0.483	0.441	0.358	0.111	1.142	0.438	-0.007	0.877	1.014	0.947	0.923	0.568	1.090	0.352	0.935	0.539
19	rs12196873	-0.578	0.139	1.184	0.128	1.270	0.045	1.066	0.578	1.701	0.025	-0.464	0.087	0.875	0.542	0.032	0.555	0.997	0.991	0.788	0.183	1.114	0.333	0.795	0.095
20	rs2720044	1.032	0.114	0.643	0.018	0.908	0.616	0.992	0.967	-1.032	0.414	0.897	0.047	0.991	0.979	0.012	0.894	1.644	0.196	0.930	0.797	1.235	0.26	1.325	0.183
21	rs10957156	0.193	0.578	0.940	0.53	0.876	0.202	0.994	0.951	-0.148	0.826	0.305	0.203	1.287	0.204	-0.044	0.365	0.834	0.402	0.795	0.108	0.945	0.569	0.975	0.827
22	rs4879656	0.039	0.899	0.975	0.768	0.964	0.683	1.155	0.107	-0.145	0.807	0.096	0.651	1.041	0.806	0.040	0.356	0.766	0.198	0.862	0.264	1.072	0.429	1.051	0.627
23	rs10905065	-0.045	0.883	0.976	0.773	1.098	0.294	0.934	0.432	0.117	0.841	-0.238	0.256	0.985	0.923	0.064	0.129	0.843	0.381	1.138	0.322	1.153	0.096	0.867	0.154
24	rs11031006	0.284	0.491	0.921	0.481	1.230	0.098	1.097	0.449	-0.688	0.389	0.056	0.845	1.271	0.248	-0.029	0.618	1.390	0.178	0.856	0.401	1.045	0.705	0.943	0.673
25	rs6484478	-0.359	0.33	1.099	0.365	0.906	0.371	1.096	0.387	-0.116	0.871	-0.111	0.664	0.957	0.823	0.016	0.759	0.908	0.681	0.740	0.043	1.074	0.496	0.965	0.772
26	rs10734411	0.249	0.405	1.117	0.188	1.032	0.718	0.970	0.728	0.790	0.172	0.132	0.524	1.006	0.971	-0.130	0.002	1.068	0.734	1.228	0.107	1.112	0.214	1.091	0.384
27	rs2277339	0.321	0.601	1.386	0.062	1.048	0.797	1.367	0.089	1.405	0.238	-0.063	0.883	0.803	0.532	0.030	0.731	0.858	0.715	0.828	0.495	0.780	0.154	0.696	0.1
28	rs3741604	-0.197	0.511	0.842	0.043	1.017	0.851	1.174	0.063	0.328	0.572	0.188	0.365	0.683	0.018	-0.008	0.846	0.983	0.931	0.860	0.237	1.005	0.955	1.038	0.71
29	rs1183272	-0.099	0.742	1.162	0.079	1.001	0.987	0.855	0.072	-0.488	0.403	-0.293	0.161	1.571	0.005	0.000	0.995	0.872	0.491	1.085	0.529	1.013	0.878	0.907	0.333
30	rs7397861	-0.241	0.438	0.950	0.561	1.001	0.991	0.946	0.539	0.193	0.748	-0.285	0.186	0.840	0.29	-0.099	0.023	1.039	0.852	0.986	0.916	1.045	0.62	1.032	0.766
31	rs551087	-0.119	0.731	1.218	0.045	1.128	0.237	1.034	0.739	-0.456	0.497	0.344	0.152	0.906	0.592	0.061	0.211	1.197	0.443	1.186	0.264	1.119	0.255	0.890	0.31
32	rs1727326	-0.327	0.49	1.017	0.897	0.997	0.983	1.057	0.686	0.408	0.657	-0.347	0.29	0.777	0.292	0.103	0.12	0.780	0.391	0.968	0.87	1.112	0.431	0.915	0.57
33	rs12824058	-0.501	0.098	1.146	0.111	0.938	0.471	0.862	0.089	0.073	0.901	-0.144	0.493	0.995	0.975	-0.024	0.577	1.084	0.683	0.844	0.196	1.037	0.67	0.913	0.372
34	rs4886238	0.249	0.431	1.061	0.504	1.098	0.318	1.076	0.427	-0.467	0.445	-0.216	0.324	0.850	0.35	-0.033	0.455	0.696	0.099	0.983	0.897	0.902	0.251	0.918	0.425
35	rs1713460	0.077	0.849	0.940	0.591	0.890	0.328	1.200	0.124	0.363	0.644	-0.017	0.951	1.090	0.69	-0.005	0.928	1.629	0.049	0.841	0.331	0.834	0.114	1.029	0.833
36	rs9796	-0.261	0.382	1.156	0.085	0.987	0.879	0.950	0.549	-0.418	0.47	-0.170	0.412	1.300	0.103	0.072	0.083	0.893	0.565	1.160	0.248	0.875	0.117	1.036	0.72
37	rs1054875	0.135	0.667	1.239	0.016	0.938	0.489	0.951	0.579	-0.721	0.235	0.000	0.999	1.327	0.087	-0.054	0.216	0.871	0.505	1.026	0.85	1.009	0.918	1.039	0.713
38	rs9039	0.139	0.695	0.756	0.005	0.995	0.958	0.984	0.877	-0.330	0.629	-0.025	0.919	0.717	0.106	-0.068	0.167	0.996	0.987	0.919	0.582	1.247	0.03	0.909	0.427
39	rs10852344	-0.049	0.872	0.967	0.701	0.987	0.883	1.100	0.284	-0.320	0.588	0.053	0.803	0.827	0.243	-0.019	0.665	1.210	0.348	0.937	0.621	0.970	0.722	1.120	0.272
40	rs12599106	-1.012	0.006	1.236	0.044	1.174	0.144	0.883	0.249	0.071	0.922	-0.063	0.806	0.940	0.757	-0.042	0.423	0.629	0.056	0.894	0.481	0.959	0.688	0.979	0.865
41	rs8070740	0.416	0.225	1.115	0.263	1.061	0.564	1.183	0.098	-0.092	0.89	-0.301	0.206	1.324	0.108	0.017	0.723	0.961	0.862	1.152	0.325	0.856	0.109	0.964	0.749
42	rs2941505	0.034	0.912	0.989	0.897	0.840	0.061	1.128	0.18	0.184	0.761	-0.259	0.23	0.967	0.839	-0.062	0.157	0.929	0.714	1.119	0.409	1.189	0.05	1.117	0.296
43	rs1799949	0.016	0.96	1.117	0.23	0.927	0.431	1.055	0.575	-0.326	0.606	0.207	0.362	1.170	0.361	-0.065	0.157	1.224	0.33	1.183	0.221	0.998	0.982	1.019	0.861
44	rs349306	0.352	0.605	0.766	0.168	1.179	0.408	1.221	0.298	-1.271	0.335	0.193	0.682	1.067	0.861	-0.064	0.503	0.776	0.54	0.828	0.501	0.937	0.736	0.757	0.203
45	rs7259376	0.410	0.186	0.974	0.763	1.081	0.394	0.925	0.385	-0.012	0.984	-0.265	0.218	0.898	0.519	-0.037	0.399	0.689	0.065	1.017	0.899	1.108	0.244	0.960	0.695
46	rs11668344	0.078	0.81	1.053	0.576	1.050	0.61	1.096	0.336	0.370	0.556	-0.188	0.404	0.800	0.214	0.009	0.84	0.880	0.554	0.723	0.025	1.168	0.095	0.867	0.194
47	rs2547274	-0.486	0.444	1.142	0.457	0.987	0.946	0.911	0.606	0.360	0.769	0.108	0.806	0.709	0.361	-0.024	0.785	1.392	0.381	0.549	0.061	1.337	0.114	0.692	0.107
48	rs12461110	-0.345	0.325	0.978	0.818	0.896	0.287	1.163	0.141	0.012	0.986	-0.138	0.57	0.952	0.795	-0.029	0.549	0.957	0.848	1.066	0.671	1.125	0.237	0.998	0.988
49	rs451417	-1.090	0.02	1.001	0.995	1.133	0.375	1.064	0.645	-0.202	0.823	-0.131	0.686	1.122	0.635	0.053	0.418	0.502	0.083	1.064	0.754	1.094	0.5	0.797	0.169
50	rs16991615	-0.371	0.591	0.711	0.083	0.747	0.143	1.536	0.046	-0.167	0.901	0.269	0.574	0.368	0.064	0.025	0.798	1.274	0.557	0.826	0.543	0.777	0.196	0.965	0.877
51	rs2236553	0.203	0.682	0.941	0.662	1.086	0.57	0.901	0.468	-0.081	0.932	-0.509	0.138	0.966	0.896	-0.023	0.738	0.632	0.129	1.209	0.385	0.920	0.555	1.020	0.904
52	rs13040088	0.305	0.44	1.038	0.737	0.907	0.399	0.886	0.284	-0.237	0.757	-0.312	0.255	0.971	0.892	0.007	0.899	1.152	0.57	0.965	0.835	1.282	0.029	0.920	0.531
53	rs5762534	-0.081	0.845	0.818	0.087	0.928	0.541	0.960	0.733	0.552	0.491	-0.270	0.348	0.751	0.237	-0.030	0.599	1.699	0.026	1.100	0.582	0.881	0.281	1.102	0.477
54	rs763121	-0.087	0.786	0.892	0.209	1.155	0.133	0.993	0.936	0.367	0.554	0.263	0.237	0.765	0.138	0.030	0.506	1.158	0.474	0.989	0.933	0.931	0.435	1.068	0.535

		RI (n=1,603)																									
		Age		Smoking status		Alcohol drinker		Educational level		eGFR		BMI		Cardiovascular disease history		Total cholesterol		Diabetes		Statins therapy		HRT		Receiving BP-related medication	
n	SNP	β	p	OR	p	OR	p	OR	p	β	p	β	p	OR	p	β	p	OR	p	OR	p	OR	p	OR	p
1	rs4246511	-0.061	0.839	0.736	0.005	1.001	0.992	1.235	0.017	-0.150	0.804	0.033	0.854	0.994	0.962	0.020	0.617	1.076	0.558	0.920	0.494	1.248	0.428	0.975	0.76
2	rs12142240	-0.083	0.775	0.981	0.861	1.044	0.633	0.940	0.458	-0.478	0.402	-0.016	0.93	1.034	0.789	-0.007	0.847	1.269	0.058	0.924	0.514	1.672	0.074	0.895	0.161
3	rs1411478	-0.389	0.156	1.333	0.006	1.244	0.009	1.149	0.082	0.074	0.891	-0.059	0.721	1.156	0.221	0.001	0.968	0.948	0.631	1.138	0.274	1.441	0.153	0.970	0.684
4	rs2236918	0.068	0.805	1.242	0.04	1.174	0.061	1.077	0.357	0.156	0.783	0.017	0.916	1.087	0.482	0.052	0.138	0.898	0.342	0.921	0.463	0.889	0.64	0.884	0.104
5	rs704795	0.452	0.1	0.964	0.725	0.859	0.075	0.947	0.496	-0.419	0.438	0.135	0.401	1.077	0.525	0.000	0.997	1.108	0.363	0.811	0.06	1.086	0.742	1.176	0.031
6	rs1800932	0.024	0.943	1.173	0.209	1.017	0.87	1.005	0.962	-0.213	0.741	0.019	0.923	0.901	0.45	0.037	0.399	0.810	0.12	1.024	0.862	1.992	0.072	1.038	0.679
7	rs930036	-0.098	0.725	1.027	0.794	1.097	0.275	0.902	0.195	1.054	0.058	-0.039	0.81	0.812	0.073	-0.003	0.934	0.946	0.613	0.915	0.426	1.188	0.501	0.978	0.774
8	rs16858210	0.011	0.973	1.077	0.534	1.194	0.063	0.962	0.672	0.445	0.475	-0.024	0.895	1.049	0.723	-0.020	0.624	1.189	0.191	1.207	0.174	1.536	0.18	1.075	0.406
9	rs4693089	0.042	0.874	1.140	0.182	1.035	0.679	1.064	0.419	0.372	0.472	0.202	0.196	0.993	0.949	0.017	0.632	1.199	0.087	1.132	0.277	1.156	0.539	0.926	0.285
10	rs6856693	0.519	0.066	0.947	0.602	0.923	0.35	0.857	0.059	-1.071	0.054	-0.204	0.225	1.261	0.049	0.002	0.965	1.110	0.376	1.125	0.301	1.053	0.841	1.036	0.645
11	rs427394	-0.017	0.948	1.196	0.084	0.981	0.818	1.061	0.448	-0.057	0.914	-0.327	0.036	1.066	0.575	-0.014	0.692	0.903	0.356	0.957	0.686	0.833	0.439	0.958	0.555
12	rs11738223	-0.354	0.216	0.952	0.647	0.940	0.482	1.015	0.855	1.013	0.072	0.151	0.37	0.869	0.244	0.067	0.065	1.129	0.307	1.051	0.682	1.711	0.064	0.937	0.402
13	rs2241584	-0.373	0.174	0.989	0.912	1.026	0.759	0.953	0.542	1.517	0.006	-0.266	0.097	0.968	0.781	0.033	0.37	0.860	0.173	0.868	0.232	1.147	0.582	0.921	0.267
14	rs365132	-0.313	0.245	1.035	0.729	1.109	0.21	0.960	0.6	0.201	0.702	0.197	0.21	1.076	0.519	0.003	0.922	1.127	0.308	1.088	0.439	0.833	0.447	1.016	0.823
15	rs6899676	0.295	0.362	1.015	0.902	1.057	0.569	0.932	0.448	-0.955	0.138	-0.039	0.842	1.190	0.225	0.054	0.201	0.860	0.232	0.990	0.942	0.992	0.979	0.931	0.417
16	rs9393800	-0.117	0.703	0.960	0.717	1.136	0.169	1.093	0.319	1.333	0.03	-0.057	0.751	0.768	0.036	0.032	0.417	0.863	0.245	1.161	0.242	1.961	0.042	0.896	0.187
17	rs2230365	-0.011	0.976	1.163	0.299	1.009	0.937	0.991	0.932	-1.393	0.073	0.357	0.112	1.511	0.023	-0.056	0.254	1.055	0.741	1.196	0.268	1.210	0.597	1.250	0.034
18	rs707938	-0.035	0.904	0.920	0.44	0.974	0.767	0.839	0.037	1.038	0.078	-0.098	0.568	0.814	0.091	0.057	0.125	0.929	0.536	1.083	0.507	1.704	0.071	0.938	0.421
19	rs12196873	0.759	0.058	1.059	0.707	0.888	0.351	0.961	0.732	-0.530	0.507	0.445	0.059	1.140	0.457	-0.056	0.288	1.144	0.433	1.017	0.921	1.097	0.8	1.088	0.442
20	rs2720044	-0.004	0.993	0.912	0.551	0.997	0.982	0.918	0.474	0.602	0.507	-0.291	0.234	1.254	0.23	-0.023	0.667	0.986	0.934	1.092	0.619	1.849	0.199	0.902	0.365
21	rs10957156	-0.092	0.767	0.904	0.376	0.934	0.477	1.095	0.315	0.121	0.842	-0.257	0.158	1.024	0.859	0.001	0.98	1.033	0.809	1.048	0.713	0.894	0.692	0.900	0.211
22	rs4879656	0.071	0.798	1.008	0.935	0.963	0.655	0.833	0.023	0.242	0.678	0.103	0.528	1.066	0.586	-0.015	0.681	1.110	0.356	1.055	0.644	1.206	0.461	1.009	0.901
23	rs10905065	0.181	0.507	0.969	0.753	1.072	0.408	0.999	0.988	-0.240	0.653	0.235	0.143	0.946	0.631	-0.006	0.856	1.048	0.675	0.975	0.823	0.933	0.78	1.072	0.347
24	rs11031006	0.146	0.68	1.303	0.059	0.883	0.261	1.061	0.564	1.015	0.161	0.092	0.661	0.793	0.102	0.027	0.554	1.261	0.14	0.785	0.082	1.186	0.63	1.005	0.96
25	rs6484478	0.047	0.886	1.003	0.983	0.947	0.601	1.066	0.504	-1.056	0.101	0.151	0.427	0.979	0.88	0.028	0.522	0.827	0.136	0.879	0.319	0.608	0.062	1.046	0.613
26	rs10734411	0.220	0.416	0.916	0.387	0.899	0.199	0.921	0.291	-0.829	0.12	0.077	0.626	1.065	0.583	0.047	0.19	0.983	0.882	1.189	0.126	1.350	0.222	0.895	0.135
27	rs2277339	0.036	0.937	1.166	0.396	0.908	0.503	1.211	0.169	-0.539	0.557	-0.079	0.773	0.855	0.405	-0.091	0.13	1.159	0.45	1.222	0.323	1.444	0.438	0.952	0.696
28	rs3741604	-0.072	0.786	1.010	0.917	0.962	0.635	0.988	0.872	-0.615	0.243	0.065	0.679	0.866	0.21	-0.014	0.676	0.941	0.585	0.953	0.675	1.514	0.093	0.997	0.966
29	rs1183272	-0.215	0.42	0.922	0.415	0.993	0.931	1.001	0.985	-0.321	0.553	-0.054	0.732	0.922	0.476	0.012	0.732	1.006	0.959	0.969	0.782	1.491	0.121	1.032	0.669
30	rs7397861	0.593	0.033	0.878	0.222	0.969	0.716	0.941	0.452	-0.793	0.152	-0.060	0.709	1.009	0.939	0.017	0.641	1.016	0.887	0.955	0.692	0.784	0.355	1.019	0.802
31	rs551087	-0.159	0.592	0.988	0.915	1.059	0.522	0.982	0.835	-0.112	0.849	-0.138	0.428	0.867	0.247	0.040	0.295	0.960	0.73	0.974	0.83	1.214	0.489	0.906	0.223
32	rs1727326	-0.294	0.498	1.095	0.582	1.009	0.948	0.971	0.815	-0.298	0.725	0.336	0.182	1.411	0.088	-0.042	0.443	0.996	0.984	1.236	0.266	1.200	0.657	0.962	0.742
33	rs12824058	-0.064	0.816	1.004	0.971	0.881	0.135	0.940	0.436	-0.315	0.559	0.057	0.721	0.944	0.622	-0.017	0.648	0.981	0.868	0.845	0.131	0.988	0.963	0.992	0.916
34	rs4886238	0.004	0.988	1.033	0.765	0.921	0.357	1.025	0.774	0.150	0.796	-0.041	0.809	1.087	0.503	-0.024	0.534	0.965	0.766	1.130	0.365	0.806	0.394	1.105	0.209
35	rs1713460	0.123	0.669	0.920	0.432	0.934	0.447	1.004	0.964	-0.003	0.996	-0.050	0.766	0.964	0.76	-0.031	0.408	1.214	0.113	1.181	0.168	1.241	0.425	1.008	0.921
36	rs9796	0.227	0.412	1.143	0.192	1.015	0.863	0.916	0.271	0.434	0.424	-0.180	0.263	0.913	0.435	0.060	0.089	0.886	0.292	0.842	0.137	1.166	0.55	0.937	0.384
37	rs1054875	0.012	0.964	1.111	0.316	1.067	0.441	0.895	0.158	-0.258	0.645	0.117	0.465	1.092	0.454	0.005	0.891	0.983	0.878	1.036	0.755	1.004	0.988	0.990	0.898
38	rs9039	-0.093	0.752	0.987	0.907	1.135	0.161	1.075	0.393	0.173	0.764	-0.293	0.088	0.968	0.791	0.026	0.484	0.871	0.237	1.004	0.974	0.862	0.573	0.984	0.836
39	rs10852344	0.319	0.265	0.998	0.984	0.887	0.166	0.955	0.574	0.169	0.762	-0.032	0.852	1.014	0.908	0.010	0.792	1.108	0.366	0.887	0.307	0.756	0.327	1.018	0.82
40	rs12599106	-0.432	0.119	0.909	0.356	1.033	0.706	1.013	0.87	0.818	0.131	0.196	0.224	0.988	0.92	-0.023	0.532	0.869	0.234	0.960	0.72	0.895	0.655	0.893	0.135
41	rs8070740	0.715	0.023	0.921	0.476	0.925	0.428	0.973	0.761	-1.701	0.007	-0.010	0.959	1.092	0.518	-0.028	0.482	1.061	0.645	1.014	0.914	0.714	0.198	1.110	0.226
42	rs2941505	0.693	0.015	1.304	0.017	0.988	0.888	1.043	0.607	-1.081	0.06	0.228	0.175	1.247	0.078	-0.030	0.417	0.900	0.352	0.987	0.91	0.731	0.226	1.056	0.482
43	rs1799949	-0.017	0.953	0.965	0.732	0.962	0.653	1.217	0.018	0.174	0.758	0.337	0.044	1.120	0.351	-0.008	0.823	0.960	0.723	0.827	0.093	1.358	0.262	0.806	0.005
44	rs349306	0.133	0.793	1.055	0.783	1.116	0.472	1.012	0.936	-0.551	0.584	-0.130	0.664	0.759	0.173	0.039	0.555	1.088	0.701	1.328	0.219	0.797	0.593	1.377	0.025
45	rs7259376	-0.160	0.552	0.974	0.796	0.961	0.624	0.943	0.449	0.253	0.634	-0.300	0.056	0.903	0.368	0.019	0.579	1.139	0.235	0.927	0.489	0.750	0.246	0.908	0.189
46	rs11668344	-0.387	0.156	1.027	0.792	1.136	0.127	1.228	0.01	1.120	0.035	-0.127	0.43	1.004	0.972	0.027	0.453	1.065	0.579	0.945	0.618	1.381	0.207	0.893	0.128
47	rs2547274	-0.680	0.138	1.143	0.456	0.947	0.698	0.954	0.721	1.666	0.061	0.302	0.259	0.760	0.13	0.070	0.228	0.893	0.534	1.128	0.546	0.898	0.791	0.907	0.431
48	rs12461110	0.275	0.322	0.914	0.375	0.877	0.131	0.890	0.142	-1.007	0.063	0.306	0.062	1.207	0.121	0.022	0.53	0.899	0.363	1.065	0.59	1.056	0.832	1.087	0.27
49	rs451417	0.019	0.965	0.865	0.338	1.057	0.666	0.953	0.694	-0.195	0.815	0.023	0.926	0.877	0.449	0.009	0.863	0.849	0.313	0.852	0.339	1.144	0.744	1.099	0.416
50	rs16991615	-0.267	0.614	1.102	0.638	0.965	0.827	1.003	0.982	1.054	0.322	0.449	0.153	0.877	0.546	0.038	0.592	1.501	0.105	0.991	0.967	0.686	0.352	1.015	0.916
51	rs2236553	-0.215	0.524	0.998	0.987	0.981	0.849	1.059	0.56	0.488	0.478	-0.221	0.266	0.913	0.521	0.021	0.633	0.894	0.425	0.872	0.36	1.063	0.849	0.895	0.224
52	rs13040088	0.821	0.013	1.109	0.416	0.947	0.593	1.174	0.099	-0.050	0.938	-0.050	0.797	1.229	0.163	0.003	0.938	1.195	0.202	0.911	0.481	0.552	0.031	0.963	0.678
53	rs5762534	0.317	0.391	1.287	0.088	1.060	0.603	1.044	0.689	-0.609	0.409	-0.191	0.371	0.864	0.332	0.057	0.223	1.057	0.722	0.939	0.674	0.522	0.016	0.940	0.537
54	rs763121	0.526	0.069	1.032	0.773	0.919	0.35	0.925	0.343	-0.316	0.578	-0.107	0.536	0.983	0.887	0.020	0.594	1.161	0.217	1.078	0.52	0.908	0.709	1.094	0.258

		RII (n=790)																							
		Age		Smoking status		Alcohol drinker		Educational level		eGFR		BMI		Cardiovascular disease history		Total cholesterol		Diabetes		Statins therapy		HRT		Receiving BP-related medication	
n	SNP	β	p	OR	p	OR	p	OR	p	β	p	β	p	OR	p	β	p	OR	p	OR	p	OR	p	OR	p
1	rs4246511	0.048	0.918	1.018	0.901	1.067	0.604	1.171	0.196	0.898	0.284	-0.136	0.568	0.813	0.401	-0.020	0.702	1.325	0.151	1.312	0.133	0.850	0.558	1.062	0.628
2	rs12142240	-0.089	0.845	1.060	0.679	1.046	0.754	0.944	0.622	-0.391	0.636	0.625	0.008	0.847	0.503	0.042	0.421	1.110	0.573	1.122	0.501	0.957	0.876	1.093	0.465
3	rs1411478	-0.147	0.732	1.018	0.893	0.977	0.877	1.024	0.829	-0.056	0.942	-0.124	0.572	0.775	0.271	-0.019	0.692	0.878	0.443	1.099	0.554	0.777	0.332	0.944	0.612
4	rs2236918	-0.419	0.339	0.846	0.212	1.221	0.152	1.136	0.263	0.659	0.412	-0.115	0.609	0.755	0.242	0.076	0.125	0.993	0.969	1.037	0.821	1.009	0.974	0.837	0.124
5	rs704795	0.199	0.642	1.011	0.933	0.887	0.34	0.983	0.874	-0.114	0.885	0.215	0.326	1.720	0.032	0.011	0.814	0.887	0.475	0.998	0.991	1.424	0.199	1.309	0.019
6	rs1800932	-0.112	0.832	1.089	0.606	1.070	0.668	1.223	0.155	-0.336	0.726	-0.263	0.33	0.865	0.598	-0.070	0.245	1.193	0.422	1.109	0.605	1.389	0.363	0.957	0.75
7	rs930036	0.684	0.116	0.741	0.023	1.033	0.806	1.022	0.846	-0.423	0.588	-0.132	0.553	1.117	0.645	0.030	0.548	0.889	0.49	1.005	0.974	0.716	0.198	1.065	0.584
8	rs16858210	-0.469	0.335	0.878	0.372	1.045	0.728	1.014	0.913	0.734	0.399	-0.050	0.842	0.863	0.564	0.011	0.84	0.770	0.155	0.928	0.669	1.525	0.209	1.085	0.529
9	rs4693089	-0.426	0.333	1.016	0.909	0.831	0.165	0.968	0.772	0.591	0.462	-0.174	0.442	1.102	0.686	-0.059	0.24	0.884	0.483	0.881	0.436	0.957	0.869	1.308	0.022
10	rs6856693	0.217	0.63	0.808	0.127	0.894	0.455	1.196	0.121	-0.015	0.986	-0.350	0.127	0.658	0.102	-0.034	0.507	1.075	0.682	1.026	0.878	0.768	0.35	0.965	0.764
11	rs427394	-0.049	0.909	1.043	0.748	0.950	0.725	1.054	0.635	-0.354	0.646	-0.349	0.111	0.785	0.293	-0.010	0.842	0.744	0.077	1.038	0.812	1.499	0.147	0.951	0.655
12	rs11738223	0.048	0.919	1.330	0.056	0.845	0.274	0.934	0.568	-0.238	0.78	0.337	0.164	1.109	0.692	0.099	0.065	1.164	0.427	0.816	0.237	0.934	0.813	1.002	0.985
13	rs2241584	0.625	0.176	1.195	0.214	0.875	0.331	1.084	0.5	-0.222	0.789	-0.131	0.583	1.306	0.303	-0.099	0.059	1.014	0.941	0.900	0.53	0.762	0.326	1.018	0.883
14	rs365132	-0.436	0.311	1.243	0.099	0.835	0.127	0.948	0.627	0.839	0.283	-0.087	0.691	1.198	0.444	0.062	0.209	0.879	0.453	0.893	0.474	1.215	0.46	0.928	0.508
15	rs6899676	-0.464	0.348	1.587	0.006	0.991	0.952	0.816	0.1	-0.341	0.7	0.296	0.24	0.786	0.339	-0.016	0.781	1.111	0.601	1.181	0.38	1.000	1	0.989	0.932
16	rs9393800	-0.318	0.509	1.200	0.229	1.095	0.571	1.257	0.072	0.700	0.418	-0.057	0.818	1.428	0.214	0.083	0.128	0.978	0.906	0.888	0.494	1.895	0.071	1.011	0.931
17	rs2230365	0.375	0.515	0.837	0.294	1.016	0.937	1.039	0.795	-0.941	0.361	-0.319	0.278	1.601	0.209	-0.023	0.722	1.060	0.803	1.317	0.232	0.936	0.847	1.266	0.137
18	rs707938	0.559	0.214	1.181	0.232	1.002	0.991	1.041	0.724	-1.362	0.094	0.162	0.483	0.941	0.8	0.028	0.587	0.810	0.229	0.980	0.902	0.999	0.998	0.989	0.925
19	rs12196873	0.129	0.843	1.188	0.406	0.937	0.815	0.918	0.605	-1.073	0.36	0.128	0.702	1.090	0.812	0.028	0.706	1.255	0.412	1.151	0.572	1.629	0.303	1.216	0.271
20	rs2720044	-0.032	0.963	0.688	0.059	0.984	0.931	1.004	0.982	-0.717	0.56	0.166	0.634	1.333	0.467	-0.018	0.818	0.914	0.736	1.010	0.968	0.737	0.437	0.991	0.958
21	rs10957156	0.915	0.065	0.890	0.433	0.984	0.905	0.881	0.314	-2.263	0.012	0.155	0.542	1.751	0.084	-0.047	0.41	1.055	0.788	0.806	0.215	0.896	0.709	1.114	0.415
22	rs4879656	0.268	0.537	1.285	0.065	0.969	0.843	1.056	0.628	0.073	0.926	0.143	0.52	1.011	0.962	-0.042	0.395	1.055	0.757	1.281	0.13	1.365	0.261	1.051	0.665
23	rs10905065	0.381	0.366	0.940	0.634	0.995	0.967	1.214	0.071	-0.689	0.361	-0.374	0.084	0.817	0.392	-0.033	0.487	0.756	0.108	0.847	0.295	0.935	0.796	0.931	0.524
24	rs11031006	0.009	0.986	1.790	0.003	0.940	0.68	1.076	0.611	-0.514	0.606	-0.050	0.86	1.601	0.186	-0.071	0.257	1.240	0.357	1.106	0.631	0.964	0.912	1.040	0.792
25	rs6484478	-0.037	0.943	0.724	0.032	1.067	0.656	1.100	0.477	-0.269	0.77	0.171	0.516	0.888	0.66	-0.061	0.298	0.804	0.263	1.255	0.254	0.957	0.888	0.941	0.652
26	rs10734411	-0.093	0.835	1.031	0.824	1.009	0.949	0.956	0.701	-0.414	0.609	-0.052	0.822	0.983	0.944	-0.051	0.31	0.960	0.818	1.020	0.905	0.716	0.231	0.858	0.196
27	rs2277339	0.449	0.535	1.052	0.82	0.831	0.527	1.024	0.898	-1.487	0.251	-0.109	0.769	0.755	0.432	-0.039	0.636	1.540	0.19	0.906	0.703	2.257	0.178	1.061	0.757
28	rs3741604	0.172	0.688	1.069	0.609	1.043	0.715	1.165	0.169	0.850	0.269	-0.006	0.978	0.752	0.23	-0.036	0.462	0.651	0.013	0.931	0.651	1.277	0.353	0.984	0.883
29	rs1183272	-0.248	0.555	1.175	0.209	1.033	0.82	1.030	0.788	0.670	0.375	0.166	0.44	0.854	0.498	-0.024	0.617	0.847	0.323	1.098	0.543	1.465	0.14	1.008	0.942
30	rs7397861	0.638	0.157	0.985	0.911	1.057	0.696	1.064	0.591	-1.094	0.182	0.092	0.691	1.023	0.926	-0.067	0.191	0.940	0.732	1.277	0.134	1.021	0.939	1.129	0.303
31	rs551087	-0.408	0.399	1.150	0.354	0.940	0.693	0.959	0.733	0.274	0.752	0.061	0.806	1.578	0.113	-0.041	0.458	0.952	0.796	1.270	0.195	1.791	0.083	1.218	0.13
32	rs1727326	0.210	0.759	1.256	0.298	1.035	0.862	1.163	0.402	-2.726	0.027	0.622	0.075	1.744	0.202	0.044	0.573	0.748	0.253	0.883	0.611	0.524	0.073	1.178	0.374
33	rs12824058	0.212	0.633	0.824	0.15	1.002	0.992	0.896	0.337	-0.089	0.911	0.288	0.206	1.354	0.226	-0.048	0.337	0.836	0.306	1.194	0.284	1.194	0.521	1.043	0.719
34	rs4886238	-0.092	0.838	0.861	0.274	0.917	0.515	0.994	0.958	-0.232	0.778	-0.521	0.024	0.760	0.245	0.011	0.833	0.909	0.59	0.924	0.63	1.972	0.038	1.072	0.562
35	rs1713460	-0.371	0.408	0.824	0.15	1.006	0.967	0.932	0.541	0.199	0.807	-0.393	0.087	0.756	0.23	0.024	0.633	0.731	0.068	0.878	0.424	1.124	0.678	0.871	0.24
36	rs9796	-0.642	0.136	1.041	0.76	0.845	0.254	0.910	0.399	1.598	0.04	-0.313	0.156	1.282	0.297	-0.042	0.394	1.066	0.71	1.054	0.74	0.874	0.609	0.954	0.679
37	rs1054875	-0.046	0.915	0.941	0.644	1.147	0.312	1.066	0.569	-0.190	0.807	0.148	0.506	1.138	0.59	-0.060	0.226	1.312	0.129	1.075	0.652	0.989	0.968	1.093	0.442
38	rs9039	-0.385	0.405	0.979	0.879	0.879	0.504	0.990	0.931	-0.550	0.512	0.242	0.305	1.220	0.447	-0.015	0.767	1.238	0.265	1.113	0.535	0.777	0.352	0.957	0.717
39	rs10852344	-0.095	0.83	0.815	0.133	0.798	0.236	0.920	0.458	0.153	0.85	0.068	0.763	0.720	0.191	0.047	0.341	1.108	0.554	1.332	0.073	0.975	0.925	0.963	0.746
40	rs12599106	0.266	0.544	0.960	0.758	0.897	0.553	0.974	0.818	0.827	0.303	0.092	0.682	0.912	0.699	0.029	0.554	1.007	0.968	1.158	0.361	1.174	0.551	1.248	0.055
41	rs8070740	-1.163	0.019	1.107	0.513	1.003	0.983	0.939	0.624	0.322	0.724	-0.174	0.495	1.240	0.452	-0.090	0.112	1.015	0.942	1.350	0.126	1.171	0.62	0.878	0.313
42	rs2941505	0.069	0.879	0.940	0.657	0.759	0.056	1.078	0.521	0.572	0.49	0.004	0.987	0.950	0.835	-0.015	0.769	1.123	0.527	0.794	0.158	1.605	0.121	0.884	0.3
43	rs1799949	-0.617	0.178	1.136	0.371	1.082	0.535	0.902	0.371	1.076	0.19	0.112	0.631	0.758	0.248	-0.015	0.776	0.841	0.328	1.221	0.252	0.931	0.796	0.854	0.187
44	rs349306	1.712	0.217	0.521	0.098	0.745	0.563	0.684	0.284	-3.956	0.117	0.263	0.711	1.022	0.977	0.032	0.838	0.925	0.885	0.938	0.9	0.591	0.494	1.754	0.147
45	rs7259376	-0.266	0.528	1.031	0.812	0.849	0.326	0.982	0.864	0.159	0.834	-0.262	0.223	0.749	0.204	-0.015	0.756	1.182	0.321	0.837	0.251	0.728	0.22	0.973	0.803
46	rs11668344	-0.423	0.341	1.030	0.828	0.875	0.354	1.082	0.495	0.797	0.322	0.138	0.545	0.940	0.798	-0.040	0.427	0.939	0.72	0.948	0.743	1.763	0.059	0.992	0.942
47	rs2547274	-0.722	0.346	1.141	0.586	0.975	0.903	0.717	0.068	1.598	0.256	-0.707	0.07	1.514	0.39	-0.116	0.185	0.527	0.013	1.911	0.064	5.940	0.069	1.087	0.684
48	rs12461110	-0.634	0.16	0.842	0.208	1.066	0.628	1.020	0.865	1.020	0.212	-0.083	0.719	0.960	0.866	-0.052	0.308	0.779	0.154	0.857	0.346	1.198	0.525	1.036	0.765
49	rs451417	0.230	0.735	1.028	0.896	1.104	0.621	1.008	0.966	-0.845	0.49	0.437	0.21	0.973	0.94	-0.117	0.127	1.043	0.878	1.210	0.472	1.862	0.239	1.266	0.207
50	rs16991615	-1.081	0.179	1.009	0.972	0.797	0.388	0.958	0.836	0.774	0.593	0.007	0.987	0.850	0.692	0.003	0.972	0.897	0.725	0.931	0.804	0.528	0.107	0.742	0.142
51	rs2236553	0.273	0.588	1.106	0.521	1.173	0.301	0.844	0.187	-0.841	0.355	-0.209	0.417	1.334	0.328	0.062	0.278	0.676	0.036	1.209	0.325	1.870	0.092	1.091	0.515
52	rs13040088	-0.592	0.247	0.982	0.906	1.020	0.914	1.367	0.024	-1.463	0.114	-0.275	0.293	1.177	0.575	0.029	0.612	1.028	0.894	0.796	0.204	0.658	0.14	0.923	0.55
53	rs5762534	0.411	0.485	0.835	0.299	0.802	0.359	0.910	0.529	0.372	0.372007	-0.043	0.886	0.927	0.808	-0.023	0.735	0.914	0.692	0.996	0.985	0.746	0.376	0.900	0.488
54	rs763121	0.205	0.652	0.966	0.804	1.004	0.976	1.139	0.269	0.018	0.983	0.191	0.411	0.906	0.685	0.020	0.697	0.872	0.442	0.889	0.474	1.059	0.838	0.943	0.622

		RIII (n=921)																							
		Age		Smoking status		Alcohol drinker		edtyp3		eGFR		BMI		cvdbase		chol		DIAB		stat in		HRT		ant HTA	
n	SNP	β	p	OR	p	OR	p	OR	p	β	p	β	p	OR	p	β	p	OR	p	OR	p	OR	p	OR	p
1	rs4246511	-0.369	0.289	1.167	0.228	1.453	0.226	1.204	0.09	0.661	0.36	0.139	0.59	1.567	0.218	-0.014	0.808	0.900	0.615	1.050	0.705	0.854	0.692	0.983	0.888
2	rs12142240	0.112	0.729	1.047	0.697	1.016	0.96	0.980	0.844	0.055	0.935	-0.203	0.393	0.409	0.002	0.122	0.018	1.013	0.947	0.796	0.055	0.845	0.652	1.110	0.374
3	rs1411478	-0.405	0.193	0.932	0.532	1.070	0.835	1.069	0.487	-0.292	0.649	-0.021	0.927	0.713	0.243	-0.064	0.202	1.083	0.679	1.163	0.197	1.661	0.194	0.983	0.88
4	rs2236918	0.250	0.422	1.036	0.756	0.920	0.818	1.006	0.947	0.228	0.729	-0.112	0.625	0.645	0.142	0.023	0.649	0.905	0.601	0.827	0.102	2.021	0.076	0.928	0.506
5	rs704795	0.096	0.75	1.224	0.068	0.896	0.663	0.956	0.634	0.601	0.343	0.092	0.679	0.997	0.992	0.040	0.413	0.903	0.58	1.062	0.594	1.193	0.624	1.289	0.021
6	rs1800932	0.446	0.223	0.918	0.513	0.971	0.939	0.949	0.643	-1.065	0.171	0.256	0.346	1.406	0.379	-0.082	0.164	1.136	0.583	0.975	0.851	0.934	0.871	1.104	0.458
7	rs930036	0.393	0.209	1.037	0.747	0.970	0.925	1.201	0.062	0.127	0.847	-0.229	0.323	0.971	0.921	0.036	0.475	0.995	0.981	1.034	0.776	0.494	0.051	1.133	0.272
8	rs16858210	0.391	0.27	1.031	0.815	0.867	0.629	0.960	0.711	0.422	0.561	0.169	0.519	0.714	0.277	-0.003	0.964	1.273	0.298	1.204	0.173	1.650	0.304	0.847	0.181
9	rs4693089	0.055	0.858	0.970	0.787	0.987	0.962	1.086	0.392	-0.232	0.717	-0.040	0.861	1.364	0.293	0.018	0.717	1.005	0.977	1.202	0.11	1.194	0.627	0.938	0.562
10	rs6856693	0.132	0.672	0.935	0.552	0.758	0.289	1.004	0.966	-0.617	0.336	0.213	0.354	1.329	0.327	-0.002	0.974	1.245	0.247	0.857	0.19	1.039	0.917	0.964	0.743
11	rs427394	-0.423	0.161	1.141	0.23	1.008	0.979	1.044	0.648	0.555	0.374	0.119	0.595	1.190	0.553	-0.006	0.908	1.150	0.454	1.250	0.05	0.585	0.128	1.040	0.716
12	rs11738223	-0.495	0.136	1.012	0.923	1.198	0.641	0.968	0.752	1.132	0.105	0.066	0.788	1.141	0.681	0.052	0.33	0.586	0.006	1.042	0.739	0.615	0.187	1.009	0.939
13	rs2241584	0.214	0.495	1.104	0.387	1.244	0.541	1.019	0.845	-0.223	0.729	-0.186	0.421	1.099	0.753	0.053	0.29	0.909	0.616	1.037	0.758	2.216	0.065	1.173	0.161
14	rs365132	0.043	0.89	0.985	0.894	0.742	0.44	1.042	0.668	0.650	0.318	-0.084	0.713	1.849	0.042	0.002	0.968	1.123	0.541	1.057	0.628	1.842	0.102	1.297	0.02
15	rs6899676	-0.086	0.815	1.134	0.351	1.007	0.984	0.922	0.476	0.199	0.791	0.468	0.083	0.653	0.183	-0.010	0.866	1.507	0.098	0.942	0.66	0.655	0.285	0.923	0.54
16	rs9393800	0.487	0.17	0.857	0.223	1.164	0.632	0.971	0.789	-0.102	0.891	-0.488	0.064	2.152	0.066	0.036	0.528	0.884	0.56	1.264	0.088	1.031	0.942	1.059	0.656
17	rs2230365	0.219	0.61	0.824	0.198	1.583	0.247	1.409	0.013	-1.112	0.227	-0.170	0.59	1.129	0.772	0.011	0.869	1.075	0.788	1.330	0.091	1.306	0.627	0.817	0.174
18	rs707938	0.449	0.17	0.690	0.001	0.760	0.509	0.804	0.031	1.011	0.14	0.207	0.39	1.362	0.345	0.018	0.738	1.046	0.823	0.894	0.352	0.577	0.13	1.015	0.897
19	rs12196873	-0.166	0.715	0.892	0.477	1.002	0.997	1.322	0.055	0.307	0.747	-0.122	0.716	1.053	0.907	-0.016	0.829	0.693	0.143	1.052	0.767	4.993	0.123	0.904	0.528
20	rs2720044	-0.208	0.655	1.246	0.207	0.682	0.412	0.921	0.562	0.098	0.92	-0.446	0.191	0.911	0.827	-0.022	0.768	1.041	0.888	0.859	0.367	0.824	0.709	1.090	0.611
21	rs10957156	-0.278	0.416	1.075	0.562	0.869	0.739	1.115	0.309	0.697	0.321	0.140	0.579	1.464	0.286	-0.061	0.266	1.166	0.479	1.262	0.078	0.515	0.073	1.235	0.095
22	rs4879656	0.105	0.732	1.134	0.261	0.795	0.546	0.918	0.362	0.292	0.654	0.008	0.973	1.092	0.762	0.075	0.127	1.189	0.366	0.919	0.459	0.580	0.127	1.078	0.498
23	rs10905065	-0.072	0.818	1.058	0.617	0.828	0.587	1.104	0.308	-0.020	0.976	-0.083	0.719	1.471	0.181	-0.027	0.59	0.833	0.352	1.083	0.491	0.969	0.932	1.043	0.705
24	rs11031006	0.129	0.746	1.001	0.996	0.887	0.785	1.120	0.362	0.959	0.259	-0.058	0.843	1.493	0.36	-0.077	0.229	1.158	0.567	1.004	0.978	0.753	0.509	1.128	0.41
25	rs6484478	0.128	0.724	1.110	0.437	1.111	0.801	0.950	0.649	-2.184	0.005	0.037	0.889	0.936	0.845	-0.027	0.647	1.025	0.914	0.942	0.656	1.416	0.47	0.783	0.053
26	rs10734411	-0.237	0.442	0.957	0.691	1.203	0.571	0.970	0.75	-0.226	0.73	0.349	0.125	0.948	0.855	-0.047	0.349	1.237	0.263	1.063	0.592	0.795	0.529	1.104	0.37
27	rs2277339	0.969	0.062	0.933	0.707	1.193	0.687	1.153	0.39	-0.120	0.913	0.432	0.258	1.243	0.682	-0.004	0.961	1.100	0.773	0.954	0.807	3.433	0.228	1.147	0.476
28	rs3741604	-0.021	0.947	0.829	0.099	1.549	0.206	1.026	0.796	-1.010	0.122	0.026	0.91	1.021	0.944	0.033	0.514	0.960	0.83	1.084	0.489	0.780	0.5	1.178	0.145
29	rs1183272	-0.122	0.694	0.957	0.695	1.239	0.521	1.017	0.865	-0.678	0.288	0.256	0.263	0.818	0.495	0.039	0.433	0.982	0.922	1.067	0.572	0.936	0.855	1.075	0.515
30	rs7397861	0.008	0.98	1.200	0.099	1.038	0.896	0.914	0.35	-1.154	0.069	0.139	0.542	0.744	0.337	0.009	0.853	1.123	0.536	0.885	0.294	0.930	0.843	1.071	0.533
31	rs551087	0.825	0.013	0.870	0.244	0.807	0.572	0.933	0.498	-0.936	0.185	-0.009	0.972	1.162	0.651	0.006	0.905	1.165	0.468	1.162	0.236	0.933	0.858	1.115	0.37
32	rs1727326	0.201	0.678	0.963	0.827	0.775	0.644	0.854	0.283	-0.352	0.725	-0.163	0.647	0.851	0.71	0.045	0.566	1.172	0.609	1.285	0.184	0.711	0.51	1.223	0.262
33	rs12824058	0.118	0.701	1.106	0.367	0.874	0.67	1.099	0.324	-0.101	0.873	-0.085	0.708	0.792	0.412	-0.029	0.551	1.025	0.896	0.981	0.868	0.933	0.846	0.906	0.37
34	rs4886238	0.108	0.731	0.923	0.482	1.136	0.712	1.036	0.72	1.194	0.065	-0.262	0.258	0.591	0.062	-0.018	0.718	0.935	0.725	0.899	0.362	1.166	0.696	0.954	0.678
35	rs1713460	0.069	0.832	1.047	0.699	1.027	0.93	0.912	0.359	1.069	0.125	0.036	0.883	1.167	0.626	0.050	0.345	1.270	0.251	1.045	0.717	1.182	0.671	1.120	0.337
36	rs9796	-0.027	0.927	0.988	0.907	0.975	0.931	1.055	0.561	0.343	0.576	0.260	0.233	0.571	0.049	0.000	0.997	1.172	0.388	0.926	0.488	1.215	0.582	0.808	0.045
37	rs1054875	0.035	0.913	0.902	0.365	1.079	0.843	1.050	0.616	-0.417	0.525	-0.410	0.079	1.131	0.684	-0.102	0.045	1.038	0.847	0.721	0.005	1.716	0.183	0.926	0.496
38	rs9039	0.041	0.903	1.113	0.388	1.042	0.901	0.927	0.462	-0.429	0.544	0.309	0.216	0.877	0.676	-0.063	0.246	1.706	0.022	1.126	0.353	1.783	0.208	0.902	0.393
39	rs10852344	0.070	0.817	0.894	0.315	1.196	0.527	0.867	0.13	-0.395	0.528	-0.016	0.943	1.953	0.018	0.105	0.032	0.759	0.154	0.940	0.587	1.730	0.116	1.125	0.279
40	rs12599106	0.207	0.518	0.946	0.634	1.067	0.811	0.890	0.244	-0.163	0.804	0.109	0.643	1.334	0.344	-0.069	0.179	1.017	0.931	0.915	0.456	2.328	0.031	1.141	0.249
41	rs8070740	-0.121	0.732	0.974	0.836	1.332	0.344	0.859	0.159	-0.044	0.952	-0.357	0.169	0.722	0.297	-0.014	0.812	1.264	0.306	1.152	0.291	1.176	0.708	0.982	0.883
42	rs2941505	0.078	0.808	1.003	0.976	0.853	0.65	0.854	0.112	-0.063	0.924	0.169	0.476	1.843	0.078	-0.117	0.024	1.460	0.073	1.209	0.121	0.761	0.457	1.150	0.234
43	rs1799949	-0.162	0.605	0.998	0.985	1.132	0.684	1.122	0.241	0.877	0.172	-0.292	0.204	1.355	0.333	0.010	0.847	0.796	0.222	0.877	0.253	1.278	0.536	0.919	0.451
44	rs349306	-0.479	0.367	0.912	0.625	1.107	0.859	1.110	0.529	1.963	0.071	-0.470	0.229	0.832	0.697	0.067	0.432	0.867	0.649	0.926	0.692	4.699	0.198	0.729	0.082
45	rs7259376	0.068	0.82	1.164	0.163	0.887	0.648	1.106	0.275	-0.968	0.12	-0.010	0.963	1.729	0.066	-0.034	0.485	1.054	0.773	1.103	0.381	1.634	0.191	1.144	0.212
46	rs11668344	0.197	0.528	1.160	0.197	1.078	0.789	0.993	0.941	0.165	0.8	-0.112	0.625	1.076	0.805	-0.035	0.494	1.178	0.405	1.078	0.521	0.898	0.766	0.993	0.953
47	rs2547274	0.374	0.519	1.171	0.467	1.143	0.778	0.941	0.733	-0.778	0.529	0.047	0.913	0.632	0.322	0.058	0.537	1.543	0.3	0.781	0.228	3.020	0.309	1.403	0.134
48	rs12461110	0.807	0.011	0.997	0.977	1.145	0.618	0.950	0.604	-1.598	0.015	0.282	0.226	1.022	0.942	-0.039	0.444	1.185	0.394	0.825	0.097	0.621	0.184	0.840	0.12
49	rs451417	-0.336	0.495	0.872	0.433	1.669	0.189	1.120	0.461	1.326	0.203	-0.014	0.969	0.670	0.335	0.114	0.149	0.689	0.175	0.807	0.226	1.321	0.66	1.011	0.953
50	rs16991615	-1.144	0.061	1.272	0.305	0.876	0.804	0.867	0.442	-0.999	0.428	0.008	0.986	1.722	0.456	-0.040	0.685	1.411	0.423	1.354	0.22	2.230	0.432	0.805	0.303
51	rs2236553	-0.180	0.612	0.944	0.653	0.664	0.276	1.133	0.265	0.282	0.702	0.076	0.771	0.800	0.478	0.127	0.026	1.114	0.63	0.743	0.019	1.225	0.647	0.879	0.301
52	rs13040088	-0.097	0.797	0.889	0.381	0.914	0.788	1.097	0.429	1.789	0.022	0.039	0.89	1.219	0.601	-0.028	0.64	1.024	0.92	1.149	0.335	0.745	0.477	1.012	0.929
53	rs5762534	0.037	0.933	0.883	0.424	0.742	0.475	0.815	0.132	-1.179	0.221	-0.356	0.271	1.220	0.657	0.062	0.38	0.713	0.172	0.950	0.75	1.000		1.062	0.704
54	rs763121	-0.075	0.817	1.096	0.436	1.114	0.744	0.823	0.051	0.553	0.444	-0.461	0.054	1.199	0.566	0.055	0.295	0.697	0.06	1.085	0.503	1.100	0.805	0.951	0.663

TABLE S13. ASSOCIATION OF EACH SNP INCLUDED IN THE GENETIC RISK SCORE WITH BLOOD PRESSURE TRAITS BY STUDY.

		CoLaus (n=1141)						RS-I-3 (n=1,603)						RS-II-1 (n=790)						RS-III-1 (n=919)					
		SBP		DBP		Hypertension		SBP		DBP		Hypertension		SBP		DBP		Hypertension		SBP		DBP		Hypertension	
n	SNP	β	p	β	p	OR	p	β	P	β	p	OR	p	β	p	β	p	OR	p	β	p	β	p	OR	p
1	rs4246511	-0.787	0.346	-0.705	0.153	0.975	0.799	-0.223	0.79	-0.112	0.795	1.032	0.743	1.379	0.204	0.461	0.422	1.061	0.663	2.550	0.238	1.935	0.356	1.002	0.99
2	rs12142240	-0.194	0.814	-0.193	0.692	0.904	0.305	0.688	0.394	0.295	0.481	0.910	0.304	0.179	0.867	-0.515	0.362	1.120	0.396	-1.578	0.432	-1.554	0.425	1.158	0.243
3	rs1411478	0.074	0.923	-0.254	0.571	1.058	0.533	-0.963	0.207	-0.118	0.766	1.025	0.778	0.005	0.996	0.385	0.467	0.970	0.805	0.495	0.798	0.894	0.634	0.987	0.914
4	rs2236918	0.535	0.478	-0.425	0.341	1.033	0.721	0.368	0.633	0.417	0.298	0.953	0.590	-1.231	0.228	-0.808	0.134	0.859	0.231	-0.051	0.979	0.227	0.904	0.929	0.537
5	rs704795	-0.482	0.534	-0.833	0.069	0.944	0.535	0.155	0.839	-0.199	0.615	1.033	0.710	0.626	0.530	0.368	0.484	1.152	0.251	-1.684	0.369	-2.211	0.224	1.284	0.032
6	rs1800932	-0.978	0.270	-0.422	0.422	0.828	0.077	-0.984	0.285	0.270	0.571	1.150	0.195	-0.977	0.426	-0.098	0.881	0.934	0.649	-0.672	0.768	-0.757	0.733	1.028	0.844
7	rs930036	0.753	0.297	-0.019	0.964	0.985	0.856	-1.171	0.131	-0.459	0.253	1.051	0.579	0.382	0.707	0.045	0.933	0.985	0.903	-1.835	0.346	-1.368	0.469	1.204	0.127
8	rs16858210	0.141	0.867	0.268	0.592	1.123	0.246	0.418	0.635	0.132	0.773	1.112	0.302	-0.265	0.815	-0.788	0.187	1.136	0.367	2.054	0.351	1.886	0.378	0.853	0.230
9	rs4693089	0.981	0.193	0.176	0.694	1.012	0.893	0.394	0.593	-0.060	0.875	0.878	0.126	1.313	0.200	0.563	0.299	1.394	0.010	2.039	0.286	1.812	0.329	0.927	0.519
10	rs6856693	-0.096	0.898	0.034	0.940	0.940	0.493	0.545	0.487	-1.066	0.009	0.824	0.034	0.155	0.882	-0.329	0.552	1.014	0.914	2.802	0.148	2.766	0.141	1.084	0.495
11	rs427394	-0.096	0.898	0.461	0.304	1.131	0.174	-0.099	0.895	0.313	0.418	0.868	0.097	0.124	0.901	-0.061	0.908	0.946	0.655	1.911	0.308	1.680	0.356	1.131	0.287
12	rs11738223	0.175	0.826	0.471	0.318	1.130	0.196	1.658	0.037	0.518	0.209	0.979	0.812	1.227	0.265	-0.520	0.372	0.881	0.350	-1.553	0.452	-1.774	0.376	0.976	0.846
13	rs2241584	0.005	0.995	0.471	0.296	1.088	0.351	-1.380	0.070	-0.401	0.311	0.960	0.636	0.808	0.453	0.358	0.530	0.866	0.279	1.516	0.436	0.978	0.605	1.125	0.327
14	rs365132	1.261	0.093	0.482	0.278	1.287	0.005	1.173	0.116	0.279	0.472	1.097	0.280	-0.588	0.558	-0.610	0.250	0.958	0.727	-0.334	0.862	-0.761	0.684	1.286	0.035
15	rs6899676	-0.504	0.597	0.033	0.954	0.965	0.752	0.310	0.730	0.053	0.909	0.964	0.719	0.653	0.570	-0.063	0.917	0.939	0.653	1.197	0.599	0.883	0.689	0.921	0.555
16	rs9393800	-0.070	0.936	0.080	0.877	0.858	0.143	-1.387	0.105	-0.263	0.553	0.952	0.612	-1.579	0.158	0.256	0.666	0.886	0.377	-0.496	0.822	-0.281	0.896	0.909	0.478
17	rs2230365	0.922	0.370	0.645	0.290	1.112	0.384	0.422	0.688	0.331	0.543	1.188	0.172	0.739	0.581	0.747	0.292	1.118	0.512	2.261	0.395	2.301	0.373	0.765	0.089
18	rs707938	1.081	0.177	0.307	0.518	1.087	0.381	1.433	0.077	0.544	0.195	1.180	0.080	-0.862	0.411	0.003	0.995	1.021	0.871	3.256	0.110	2.487	0.209	0.975	0.841
19	rs12196873	-1.077	0.267	-1.126	0.050	0.868	0.225	1.200	0.282	0.201	0.728	1.193	0.183	0.359	0.813	-0.742	0.355	1.155	0.457	1.574	0.576	2.434	0.373	0.939	0.714
20	rs2720044	-1.302	0.420	-1.330	0.165	1.062	0.755	0.294	0.801	0.341	0.574	0.913	0.492	1.828	0.249	0.012	0.988	0.898	0.579	1.342	0.641	0.202	0.943	1.091	0.623
21	rs10957156	0.037	0.966	0.089	0.861	0.937	0.528	0.092	0.915	0.124	0.781	0.876	0.174	1.642	0.157	0.731	0.233	0.965	0.803	2.058	0.333	1.728	0.402	1.274	0.071
22	rs4879656	0.779	0.305	0.573	0.203	1.041	0.662	1.204	0.117	0.949	0.017	1.085	0.359	0.005	0.996	0.732	0.170	1.075	0.564	-0.391	0.837	-0.970	0.599	1.041	0.730
23	rs10905065	0.380	0.610	0.687	0.119	1.027	0.763	-0.624	0.409	-0.008	0.984	1.068	0.446	0.107	0.914	0.064	0.902	0.889	0.336	-0.479	0.805	0.229	0.903	1.052	0.671
24	rs11031006	0.960	0.347	0.368	0.543	0.965	0.772	0.747	0.447	-0.211	0.679	1.135	0.270	2.094	0.104	0.506	0.457	1.128	0.459	-1.824	0.462	-1.874	0.436	1.146	0.383
25	rs6484478	2.072	0.023	0.808	0.134	1.080	0.478	1.472	0.106	0.527	0.265	1.093	0.405	1.179	0.324	0.460	0.467	0.986	0.924	-4.740	0.036	-3.451	0.117	0.743	0.028
26	rs10734411	0.178	0.810	0.221	0.614	1.097	0.296	0.747	0.320	-0.141	0.718	0.889	0.176	-1.422	0.173	-0.411	0.456	0.874	0.295	-0.142	0.941	-1.310	0.482	1.154	0.224
27	rs2277339	-3.323	0.029	-1.422	0.115	0.690	0.047	-0.117	0.928	0.614	0.357	1.022	0.884	0.785	0.641	-0.508	0.568	0.995	0.979	2.795	0.387	3.280	0.296	1.036	0.859
28	rs3741604	0.683	0.357	0.150	0.733	0.940	0.486	0.765	0.300	0.485	0.205	0.959	0.624	1.075	0.28	0.878	0.095	1.018	0.882	1.997	0.304	0.969	0.607	1.098	0.435
29	rs1183272	-0.001	0.999	0.358	0.418	1.078	0.396	-0.478	0.519	0.096	0.804	0.981	0.821	0.757	0.439	0.613	0.236	1.082	0.518	-0.830	0.666	-1.108	0.553	0.998	0.987
30	rs7397861	-0.101	0.896	-0.230	0.614	0.987	0.883	-0.965	0.212	-0.583	0.146	0.998	0.982	0.418	0.691	0.336	0.546	1.099	0.466	-1.444	0.453	-2.221	0.234	1.023	0.849
31	rs551087	0.378	0.660	0.576	0.257	0.996	0.969	-0.828	0.316	-0.312	0.465	0.878	0.165	0.535	0.634	-0.075	0.899	1.355	0.036	-1.854	0.372	-1.561	0.439	1.019	0.885
32	rs1727326	1.443	0.219	1.104	0.112	1.158	0.298	0.105	0.931	-0.484	0.438	0.869	0.299	1.846	0.248	0.687	0.416	1.255	0.272	2.988	0.319	2.109	0.468	1.266	0.223
33	rs12824058	-0.538	0.473	-0.757	0.088	0.924	0.38	-0.337	0.659	0.143	0.718	1.089	0.335	0.583	0.572	0.269	0.622	1.047	0.720	-0.934	0.624	-0.983	0.595	0.877	0.260
34	rs4886238	0.144	0.854	0.874	0.059	1.049	0.607	0.414	0.608	0.396	0.344	1.043	0.651	-1.448	0.169	-0.657	0.238	1.102	0.464	-4.228	0.031	-4.069	0.033	0.935	0.571
35	rs1713460	-0.416	0.678	0.277	0.641	1.020	0.867	1.005	0.208	-0.287	0.489	0.958	0.642	-1.542	0.14	-0.069	0.901	0.847	0.190	-1.663	0.412	-2.838	0.149	1.147	0.278
36	rs9796	0.672	0.364	0.579	0.186	1.042	0.637	-0.528	0.491	-0.108	0.787	0.962	0.662	-0.479	0.634	-0.311	0.559	0.878	0.295	0.674	0.714	0.059	0.973	0.897	0.336

		CoLaus (n=1141)						RS-I-3 (n=1,603)						RS-II-1 (n=790)						RS-III-1 (n=919)					
		SBP		DBP		Hypertension		SBP		DBP		Hypertension		SBP		DBP		Hypertension		SBP		DBP		Hypertension	
n	SNP	β	p	β	p	OR	p	β	P	β	p	OR	p	β	p	β	p	OR	p	β	p	β	p	OR	p
37	rs1054875	-0.290	0.709	-0.182	0.693	1.065	0.495	-0.146	0.848	-0.224	0.571	0.920	0.339	-1.129	0.264	-0.227	0.671	0.944	0.643	1.108	0.573	2.247	0.239	0.848	0.172
38	rs9039	0.759	0.385	0.214	0.678	0.938	0.537	0.414	0.612	0.080	0.849	1.008	0.931	-1.106	0.304	-0.429	0.451	0.947	0.677	-2.448	0.244	-3.577	0.079	0.808	0.092
39	rs10852344	0.396	0.600	0.131	0.770	1.074	0.427	-2.503	0.002	-1.064	0.010	1.015	0.873	1.023	0.317	-0.256	0.636	1.017	0.895	0.734	0.698	1.070	0.560	1.037	0.757
40	rs12599106	-1.265	0.170	-0.815	0.135	0.869	0.201	-0.803	0.296	-0.126	0.752	0.942	0.498	1.657	0.104	0.539	0.318	1.369	0.014	-1.996	0.315	-2.474	0.199	1.149	0.256
41	rs8070740	-0.872	0.305	-0.667	0.185	0.871	0.176	1.059	0.227	0.233	0.609	1.027	0.791	-0.535	0.646	0.000	0.999	0.889	0.402	-2.781	0.204	-2.499	0.240	1.039	0.778
42	rs2941505	-0.220	0.776	0.361	0.429	1.018	0.847	0.911	0.251	0.295	0.473	0.967	0.710	-1.293	0.221	-0.545	0.33	0.874	0.297	0.896	0.654	0.639	0.742	1.082	0.527
43	rs1799949	0.216	0.790	0.109	0.821	1.090	0.371	0.542	0.491	0.613	0.133	0.768	0.003	0.240	0.822	-0.168	0.766	0.974	0.840	1.929	0.320	2.133	0.258	0.931	0.543
44	rs349306	-3.019	0.073	-1.384	0.166	0.561	0.004	0.230	0.871	-0.479	0.514	1.570	0.011	4.680	0.147	0.628	0.714	1.734	0.202	2.461	0.456	2.543	0.427	0.718	0.084
45	rs7259376	-0.556	0.469	-0.572	0.208	0.943	0.518	-0.301	0.687	-0.634	0.101	0.970	0.721	-0.864	0.378	-0.251	0.628	0.967	0.785	2.195	0.237	1.935	0.283	1.185	0.139
46	rs11668344	-0.047	0.953	-0.330	0.488	0.973	0.773	-0.972	0.200	-0.409	0.299	0.964	0.670	-0.430	0.678	-0.348	0.526	1.091	0.498	0.096	0.960	0.296	0.875	1.001	0.994
47	rs2547274	-0.385	0.806	0.291	0.754	0.753	0.136	-1.989	0.118	-1.354	0.040	0.924	0.578	0.352	0.844	0.751	0.426	1.188	0.448	3.414	0.343	3.360	0.337	1.210	0.412
48	rs12461110	-1.549	0.074	-0.400	0.436	0.892	0.270	1.470	0.057	0.160	0.690	1.035	0.701	0.488	0.642	-0.432	0.437	1.171	0.233	1.366	0.489	1.484	0.439	0.798	0.063
49	rs451417	-1.874	0.106	-1.412	0.039	0.841	0.219	0.265	0.821	-0.106	0.861	0.962	0.773	-3.413	0.031	-0.740	0.376	1.196	0.381	2.197	0.473	2.728	0.358	1.322	0.155
50	rs16991615	-1.104	0.518	-0.627	0.536	0.968	0.874	1.095	0.457	1.045	0.171	0.833	0.258	0.023	0.990	0.052	0.958	0.946	0.808	2.341	0.539	2.078	0.574	0.715	0.126
51	rs2236553	-0.761	0.535	0.160	0.825	0.901	0.474	0.269	0.774	-0.129	0.790	0.924	0.461	-1.370	0.242	-0.306	0.621	1.024	0.869	-2.297	0.297	-3.103	0.146	0.902	0.441
52	rs13040088	0.354	0.717	0.888	0.125	1.139	0.261	-0.248	0.789	0.567	0.238	0.936	0.531	-1.146	0.338	-0.518	0.413	0.913	0.531	0.477	0.839	-0.755	0.741	1.005	0.975
53	rs5762534	2.952	0.004	0.260	0.669	1.262	0.056	-1.724	0.093	-0.320	0.548	1.012	0.922	-0.599	0.662	-0.421	0.561	0.899	0.519	-2.532	0.354	-2.048	0.440	1.105	0.561
54	rs763121	0.195	0.805	0.447	0.341	0.950	0.587	1.608	0.045	-0.040	0.923	0.983	0.85	-0.893	0.398	-0.744	0.183	0.945	0.663	0.712	0.724	1.110	0.570	0.974	0.829

Bonferroni correction for significant threshold p=0.0009259259

Beta estimates correspond to adjusted values (age, eGFR).

TABLE S14. CAUSAL ESTIMATES DERIVED FROM INSTRUMENTAL VARIABLE ANALYSIS GENETIC RISK SCORE WITH SNPS RELATED AND NON-RELATED TO DNA DAMAGE-RESPONSE (DDR) GENES.

	CoLaus (n=1139)		RS-I-3(n=1603)		RS-II-1(n=790)		RS-III-1(n=919)		Meta-analysis	
Variable	β (95% CI)	p	β (95% CI)	p	β (95% CI)	p	β (95% CI)	p	β (95% CI)	I^2
Genetic Risk Score with 37 SNPs related to DNA damage-response (DDR) genes										
Crude										
Systolic BP	-2.17 (-6.62; 2.29)	0.34	0.65 (-0.59;1.89)	0.30	-0.52 (-2.33;1.27)	0.57	1.37 (-0.51; 3.25)	0.15	0.42 (-0.46; 1.30)	13.1%
Diastolic BP	-0.61 (-2.85; 1.62)	0.59	-0.35 (-0.99;0.28)	0.28	-0.39 (-1.32;0.45)	0.38	0.39 (-0.64; 1.43)	0.45	-0.23 (-0.68; 0.22)	0.0%
*Hypertension	0.93 (0.61;1.42)	0.77	1.04 (0.92;1.19)	0.51	1.04 (0.88;1.24)	0.704	1.04 (0.83;1.26)	0.68	1.04 (0.95;1.13)	0.0%
Adjusted										
Systolic BP	-1.39 (-4.04;1.35)	0.41	0.66 (-0.53;1.85)	0.28	-0.75 (-2.41; 0.90)	0.37	0.87 (-1.09;2.81)	0.39	0.21 (-0.63; 1.04)	4.9%
Diastolic BP	-0.32 (-2.16;1.51)	0.73	-0.25 (-0.88;0.36)	0.42	-0.52 (-1.42;0.37)	0.25	0.30 (-0.79;1.40)	0.58	-0.27 (-0.71; 0.18)	0.0%
*Hypertension	0.90 (0.58;1.41)	0.67	1.06 (0.93; 1.22)	0.39	1.06 (0.88; 1.29)	0.52	1.04 (0.85;1.28)	0.66	1.05 (0.95;1.15)	0.0%
Genetic Risk Score with 17 SNPs non-related to DNA damage-response (DDR) genes										
Crude										
Systolic BP	3.77 (-4.02;11.57)	0.34	-4.79 (-8.07;-1.51)	0.004	-1.81 (-8.67; 5.05)	0.61	0.45 (-3.17; 4.08)	0.81	-1.88 (-4.08; 0.32)	54.8%
Diastolic BP	1.93 (-2.33; 6.19)	0.38	-1.27 (-2.64;0.10)	0.07	0.56 (-2.67;3.81)	0.73	-0.15 (-2.19;1.89)	0.88	-0.60 (-1.64; 0.44)	0.0%
*Hypertension	1.70 (0.87;3.32)	0.12	0.76 (0.60;0.98)	0.03	1.21 (0.641;2.29)	0.553	0.98 (0.68;1.41)	0.93	0.90 (0.75;1.08)	52.6%
Adjusted										
Systolic BP	1.73 (-5.89; 9.35)	0.66	-4.44 (-7.67; -1.22)	0.007	-3.66 (-11.75; 4.43)	0.38	-0.19 (-3.54;3.16)	0.91	-2.16 (-4.30; -0.02)	31.6%
Diastolic BP	2.91 (-3.33;9.16)	0.36	-1.39 (-2.81; 0.02)	0.05	0.43 (-3.11;3.97)	0.81	-0.01 (-1.88;1.86)	0.99	-0.66 (-1.72; 0.40)	3.5%
*Hypertension	1.54 (0.74;3.22)	0.25	0.80 (0.62;1.04)	0.09	1.18 (0.58;2.39)	0.66	0.84 (0.58; 1.25)	0.41	0.88 (0.72;1.07)	13.3%

*OR= Odds ratio; CI=Confidence interval; p=p value

TABLE S15. OUTLIER SNPS IDENTIFIED IN THE MR-PRESSO

SNP	p value
rs10852344	0.044
rs5762534	0.044
rs707938	<0.004
rs930036	<0.004
rs9796	0.012

TABLE S16. CAUSAL ESTIMATES OF ANM IN SYSTOLIC AND DIASTOLIC BLOOD PRESSURE FROM SNPS RELATED AND NON-RELATED TO DDR GENES USING METHODS IMPLEMENTED IN MR-BASE WITH DATA FROM GWAS OF ANM AND UK BIOBANK

Outcome	Method	β	SE	p-value	P_h	Q-statistics
29 SNPs*						
Diastolic BP	Weighted median	0.082	0.059	0.165	$2.22e^{-12}$	114.2392
	Inverse Variance Weighted	0.071	0.076	0.353		
	MR-PRESSO	0.086	0.054	0.125		
	MR-Egger	0.085	0.169	0.618		
Systolic BP	Weighted median	0.357	0.096	<0.001	$6.10e^{-11}$	105.6183
	Inverse Variance Weighted	0.291	0.120	0.0151		
	MR-PRESSO	0.312	0.090	0.002		
	MR-Egger	0.329	0.266	0.226		
17 SNPs**						
Diastolic BP	Weighted median	-0.030	0.105	0.772	$1.40e^{-07}$	63.36921
	Inverse Variance Weighted	-0.046	0.135	0.732		
	MR-PRESSO	-0.042	0.091	0.650		
	MR-Egger	0.279	0.542	0.614		
Systolic BP	Weighted median	0.040	0.189	0.831	$6.97e^{-06}$	53.21131
	Inverse Variance Weighted	0.175	0.203	0.389		
	MR-PRESSO	0.156	0.170	0.375		
	MR-Egger	0.136	0.825	0.871		

(CI) Confidence Interval; P_h, P-value for heterogeneity

* From 39 Damage DNA repair SNPs were identified in the reference GWAS of age at natural menopause Day et al. Clumping the identified SNPs jus 29 SNPs were identified as independent and included in the analyses.

** From 17 Non-Damage DNA repair SNPs were identified in the reference GWAS of age at natural menopause Day et al. The 17 SNPs were classified as independent SNPs after the implementation of the function clump in the two MR package.

TABLE S17. IDENTIFICATION OF PLEIOTROPIC SNPS

SNP	*CHR*	*Position (bp)*	*EA*	*OA*	*Gene*	*INFO*	BMI	Menarche
rs704795	2	27716494	A	G	*FNDC4*	0.999	x	
rs11738223	5	171934492	G	A	*SH3PXD2B*	0.990	x	
rs365132	5	176378574	T	G	*UIMC1*	1.000		x
rs11031006	11	30226528	A	G	*ARL14EP*	0.993		x
rs6484478	11	30306440	G	A	*FSHB*	0.999	x	
rs9039	16	9205363	C	T	*C16orf72*	0.989	x	
rs8070740	17	5331896	G	A	*RPAIN*	0.998	x	
rs1799949	17	41245466	A	G	*BRCA1*	1.000	x	

TABLE S18. TWO-SAMPLE MENDELIAN RANDOMIZATION EXCLUDING SNPS ASSOCIATED WITH BMI AND MENARCHE

	BMI (37 SNPs)			Menarche (38 SNPs)		
Outcome/Method	Beta	SE	p-value	Beta	SE	p-value
Diastolic blood pressure						
Weighted median	0.051	0.056	0.362	0.018	0.054	0.741
Inverse Variance Weighted	0.052	0.067	0.439	0.059	0.068	0.386
MR-Egger	0.988	0.153	0.522	0.110	0.153	0.476
MR-Egger Intercept	-0.010	0.031	0.737	-0.011	0.029	0.710
MR-PRESSO	0.058	0.051	0.263	0.066	0.052	0.214
Systolic blood pressure						
Weighted median	0.366	0.096	<0.001	0.354	0.094	<0.001
Inverse Variance Weighted	0.252	0.109	0.021	0.277	0.108	0.010
MR-Egger	0.300	0.247	0.249	0.300	0.243	0.237
MR-Egger Intercept	-0.008	0.049	0.865	-0.005	0.047	0.923
MR-PRESSO	0.259	0.099	0.013	0.265	0.084	0.003

TABLE S19. TWO-SAMPLE MENDELIAN RANDOMIZATION INCLUDING ALL SNPS

	53 SNPs		
Outcome/Method	Beta	SE	p-value
Diastolic Blood pressure			
Weighted median	0.044	0.050	0.381
Inverse Variance Weighted	0.046	0.054	0.394
MR-Egger	0.115	0.129	0.373
MR-Egger Intercept	-0.015(-0.064;0.034)		
Systolic Blood pressure			
Weighted median	0.132	0.083	0.112
Inverse Variance Weighted	0.209	0.086	0.015
MR-Egger	0.268	0.207	0.194
MR-Egger Intercept	-0.013(-0.091; 0.066)		

Figure S1. Flow chart of Study sample.

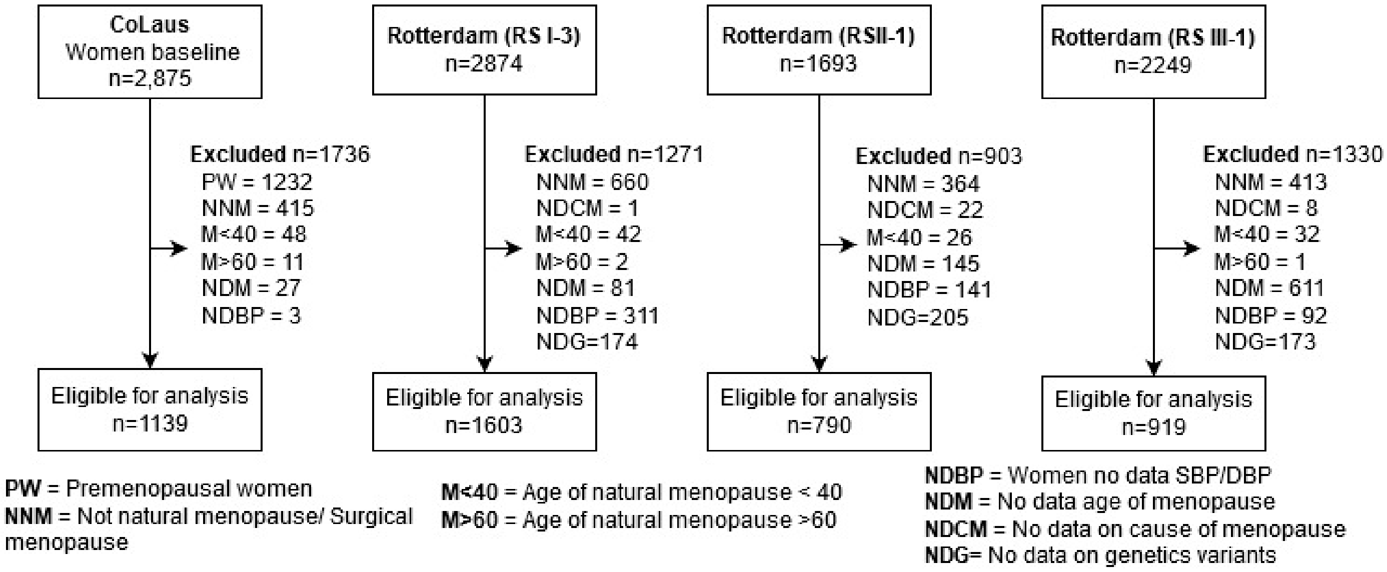

Figure S2. Non-linear spline models.

Figure II.a Colaus

Spline age natural menopause SBP

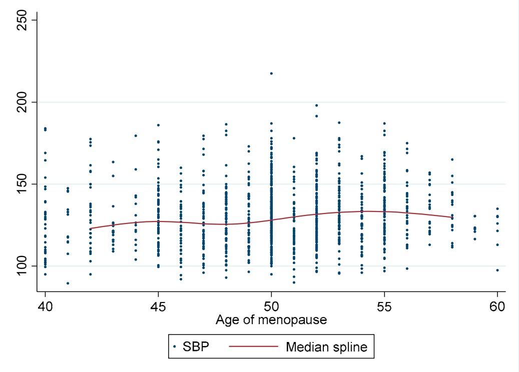

Spline age natural menopause DBP

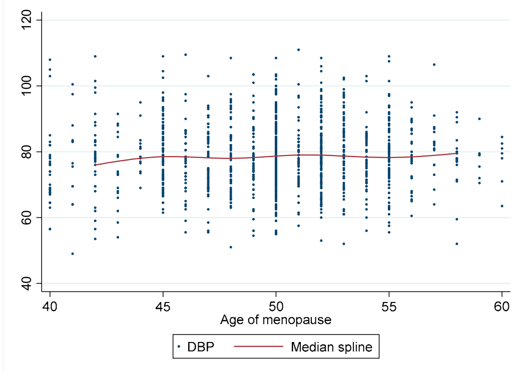

Figure II.b RSI

Spline age natural menopause SBP

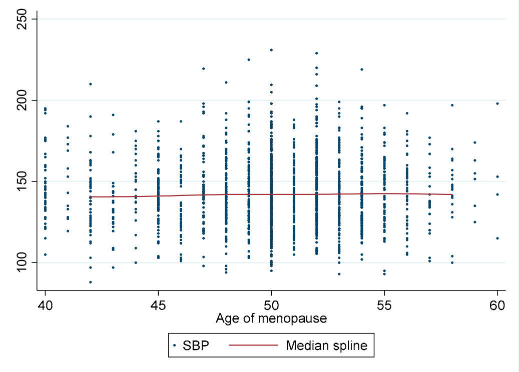

Spline age natural menopause DBP

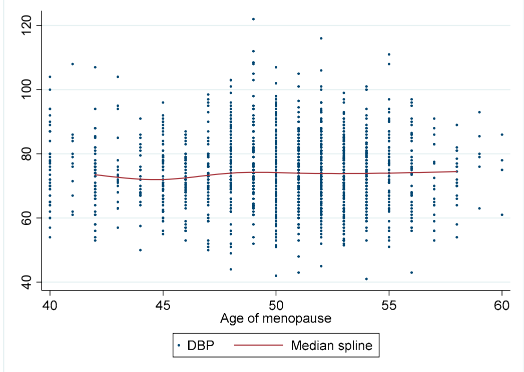

Figure II.c RSII

Spline age natural menopause SBP

Spline age natural menopause DBP

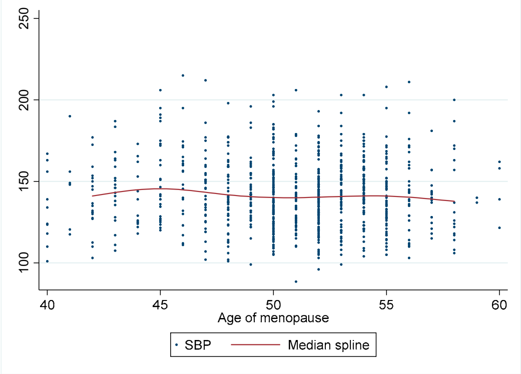

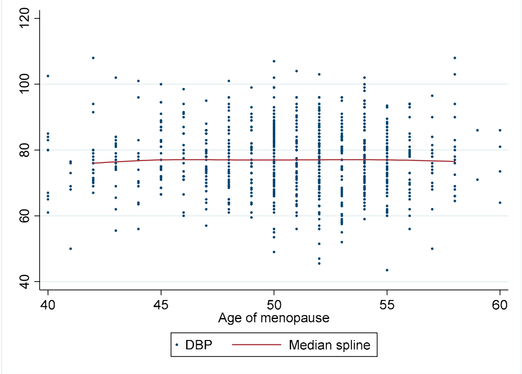

Figure II.d RSIII

Spline age natural menopause SBP	Spline age natural menopause DBP

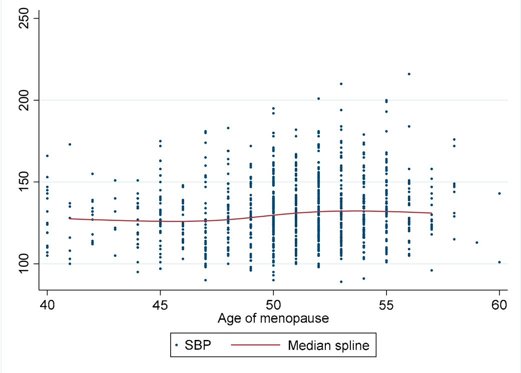

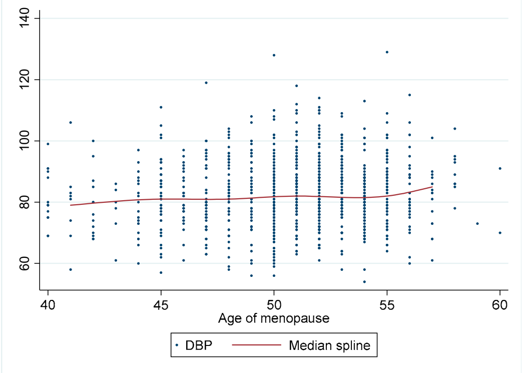

Figure S3. Sensitivity analysis observational association ANM and Blood pressure traits

Figure S3a. Age of natural menopause association with Blood Pressure in women without Hormone Therapy

ANM association with SBP in women without HT

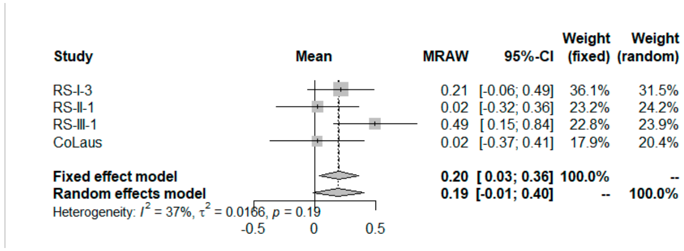

ANM association with DBP in women without HT

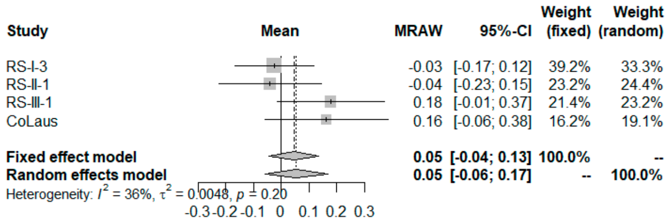

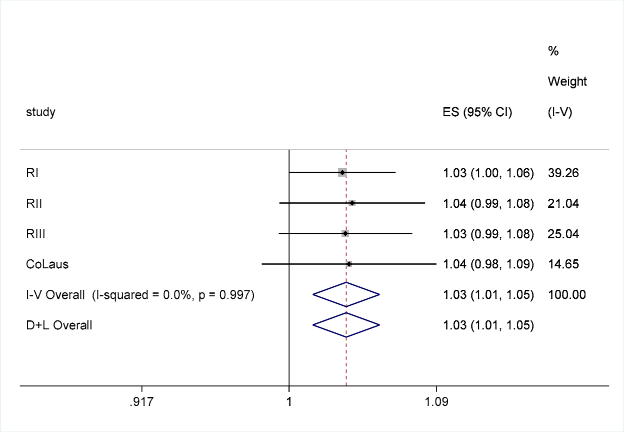

RS-I-3 n= 1466; RS-II-1 n= 759; RS-III-1 n= 898; CoLaus n= 499. 827 observations deleted which include missing values and women who reported the use of hormone replacement therapy. Regressions by study were not adjusted by Hormone replacement therapy.

Figure S3b. Age of natural menopause association with Blood Pressure traits in women aged 65 years and younger.

ANM association with SBP in women <=65 years

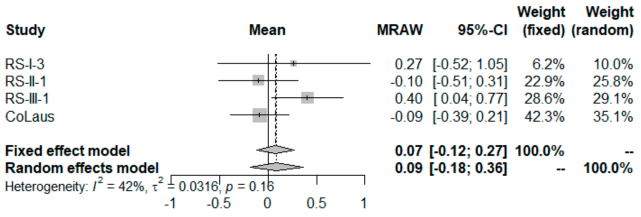

ANM association with DBP in women <=65 years

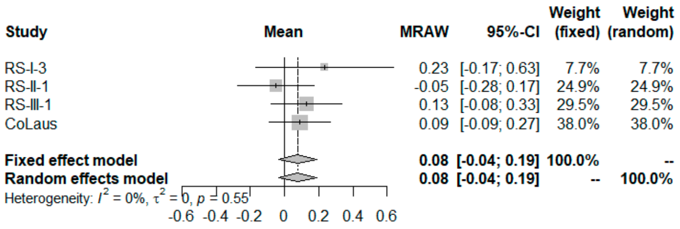

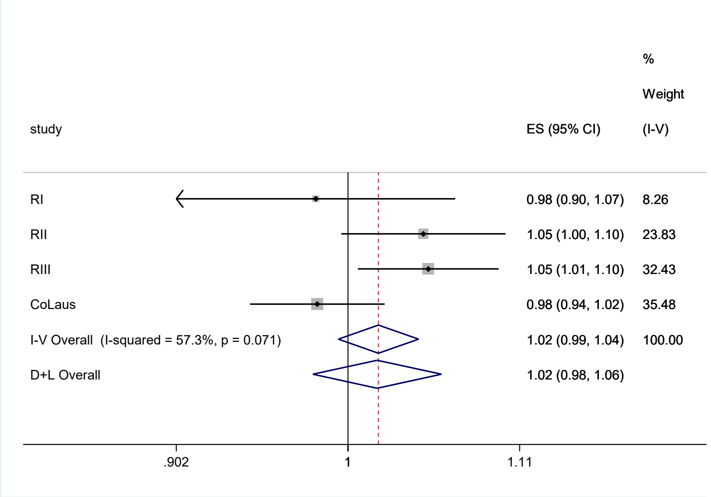

RS-I-3 n= 235; RS-II-1 n= 518; RS-III-1 n=826; CoLaus n=818. 2,052 observations deleted which include women older than 65 years old
Regressions by study were fully adjusted.

Figure S3c. Age of natural menopause association with Blood Pressure traits in women without history of cardiovascular diseases.

ANM association with SBP in women without history of CVD

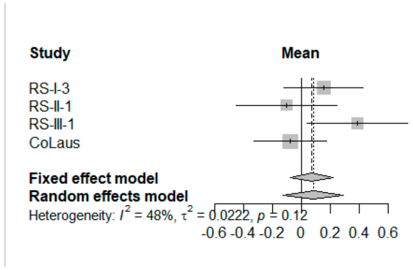

ANM association with DBP in women without history of CVD

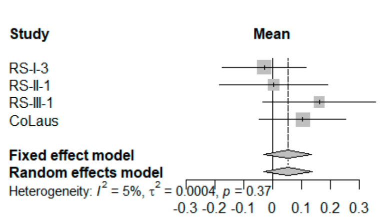

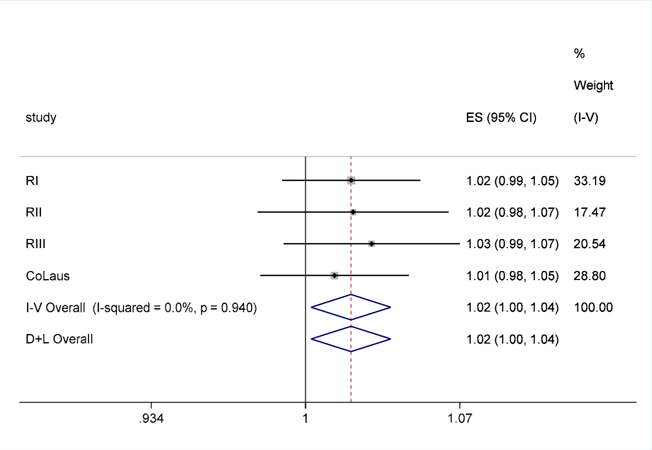

RS-I-3 n=1414; RS-II-1 n=746; RS-III-1 n=887; CoLaus n=1052. 350 observations deleted which include women who reported history of cardiovascular diseases and missing values. Regressions by study were not adjusted by history of cardiovascular diseases.

Figure S3d. Age of natural menopause association with Blood Pressure traits in Women with maximum 10 years after menopause

ANM association with SBP in women <=10 years menopause

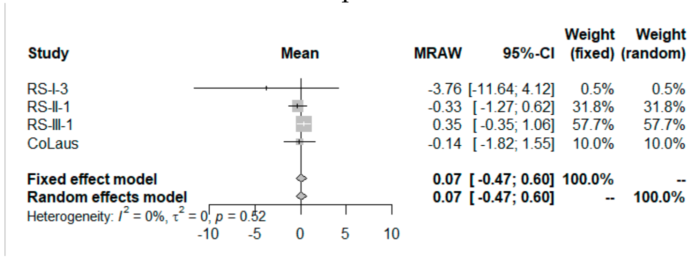

ANM association with DBP in women <=10 years menopause

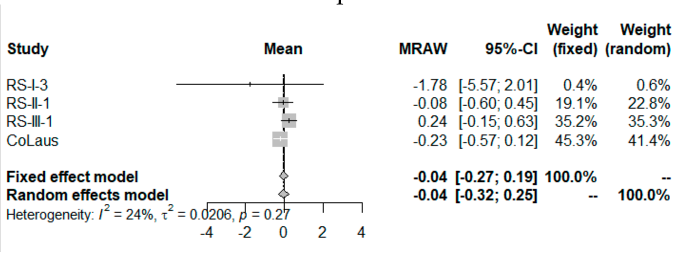

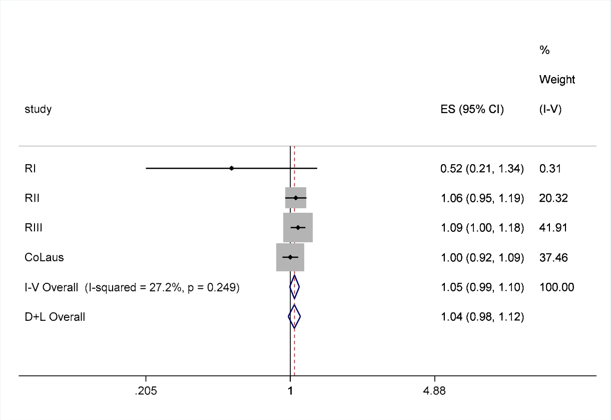

RS-I-3 n=51; RS-II-1 n=312; RS-III-1 n=611; CoLaus n=570. 2,905 observations deleted which include missing values and women who reported experiencing menopause more than 10 years ago

Regressions by study were fully adjusted

Use of antihypertensive medication was excluded from the HTA regressions

Figure S3e. Age of natural menopause association with Blood Pressure traits in Women without hypertension.

ANM asocciation with DBP in women without hypertension

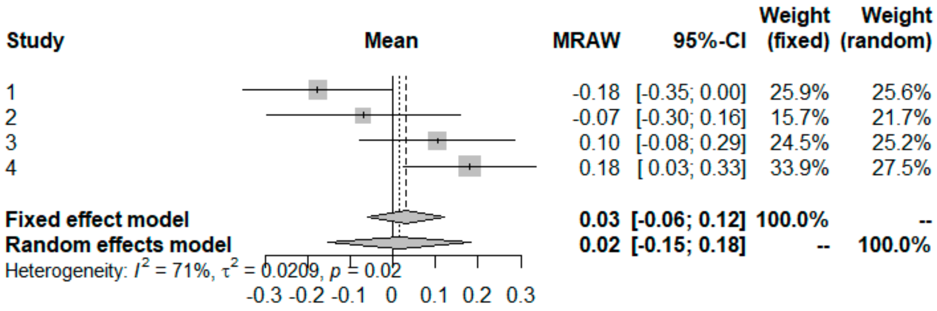

ANN asocciation wuth SBP un winen without hypertension

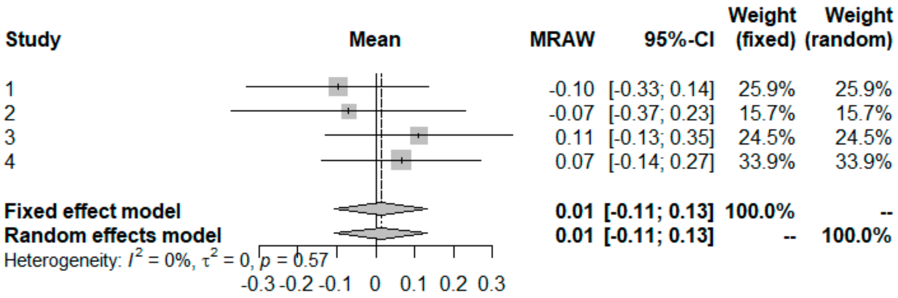

Study 1:RS-I-3 n=493; Study 2: RS-II-1 n=298; Study 3: RS-III-1 n=465; Study 4: CoLaus n=645. 2,548 women who had hypertension were deleted
Regressions by study were fully adjusted
Use of antihypertensive medication was excluded
Pooled analysis regressions were adjusted by cohort

Figure S3f. Age of natural menopause association with hypertension defined antihypertensive medication

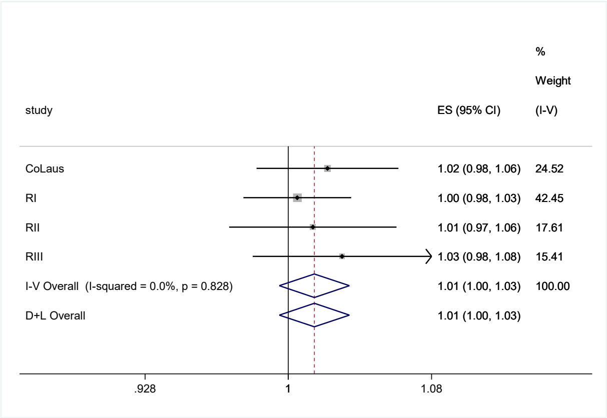

Prevalence of hypertension defined as antihypertensive medication CoLaus n=262(23.0); RS-I-3 n=614(38.3); RS-II-1 n=226(28.6); RS-III-1 n=223(24.2)

Figure S3g. Age of natural menopause association with hypertension defined antihypertensive medication in women with estimated glomerular filtration rate smaller than 60 ml/min/1.73m2

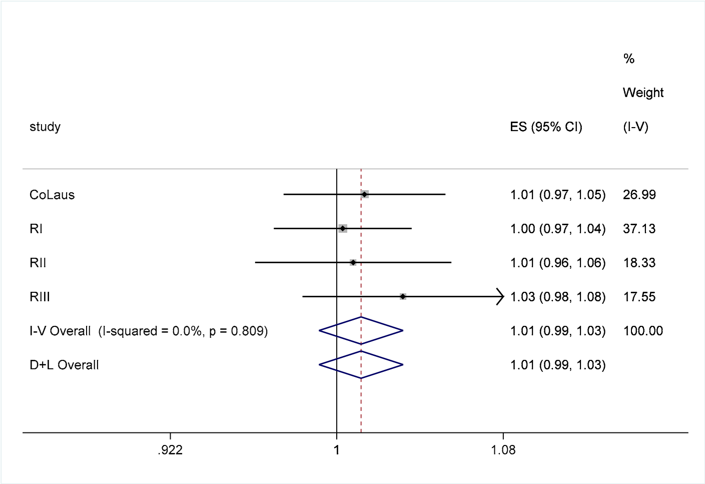

Study 1:RS-I-3 n= 1,237; Study 2: RS-II-1 n= 708; Study 3: RS-III-1 n= 878; Study 4: CoLaus n= 1,033; 593 observations deleted

Regressions by study were fully adjusted

FIGURE S4. ASSOCIATION OF GENETIC RISK SCORE OF ANM AFTER THE "LEAVE-ONE-OUT" ANALYSES.

CoLaus

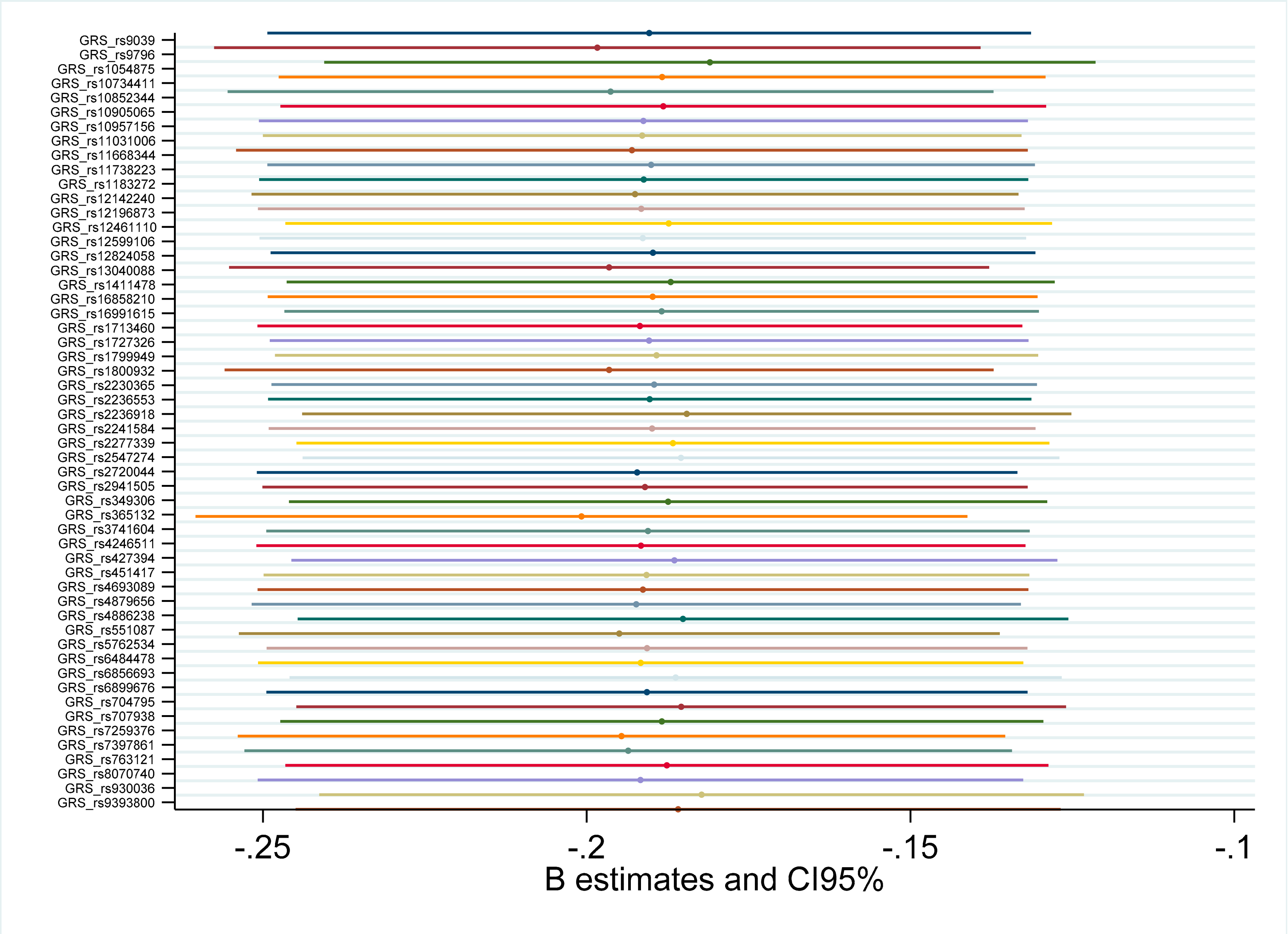
GRS_rs9039
GRS_rs9796
GRS_rs1054875
GRS_rs10734411
GRS_rs10852344
GRS_rs10905065
GRS_rs10957156
GRS_rs11031006
GRS_rs11668344
GRS_rs11738223
GRS_rs1183272
GRS_rs12142240
GRS_rs12196873
GRS_rs12461110
GRS_rs12599106
GRS_rs12824058
GRS_rs13040088
GRS_rs1411478
GRS_rs16858210
GRS_rs16991615
GRS_rs1713460
GRS_rs1727326
GRS_rs1799949
GRS_rs1800932
GRS_rs2230365
GRS_rs2236553
GRS_rs2236918
GRS_rs2241584
GRS_rs2277339
GRS_rs2547274
GRS_rs2720044
GRS_rs2941505
GRS_rs349306
GRS_rs365132
GRS_rs3741604
GRS_rs4246511
GRS_rs427394
GRS_rs451417
GRS_rs4693089
GRS_rs4879656
GRS_rs4886238
GRS_rs551087
GRS_rs5762534
GRS_rs6484478
GRS_rs6856693
GRS_rs6899676
GRS_rs704795
GRS_rs707938
GRS_rs7259376
GRS_rs7397861
GRS_rs763121
GRS_rs8070740
GRS_rs930036
GRS_rs9393800
-.25
-.2
-.15
-.1
B estimates and CI95%

RS-I-3

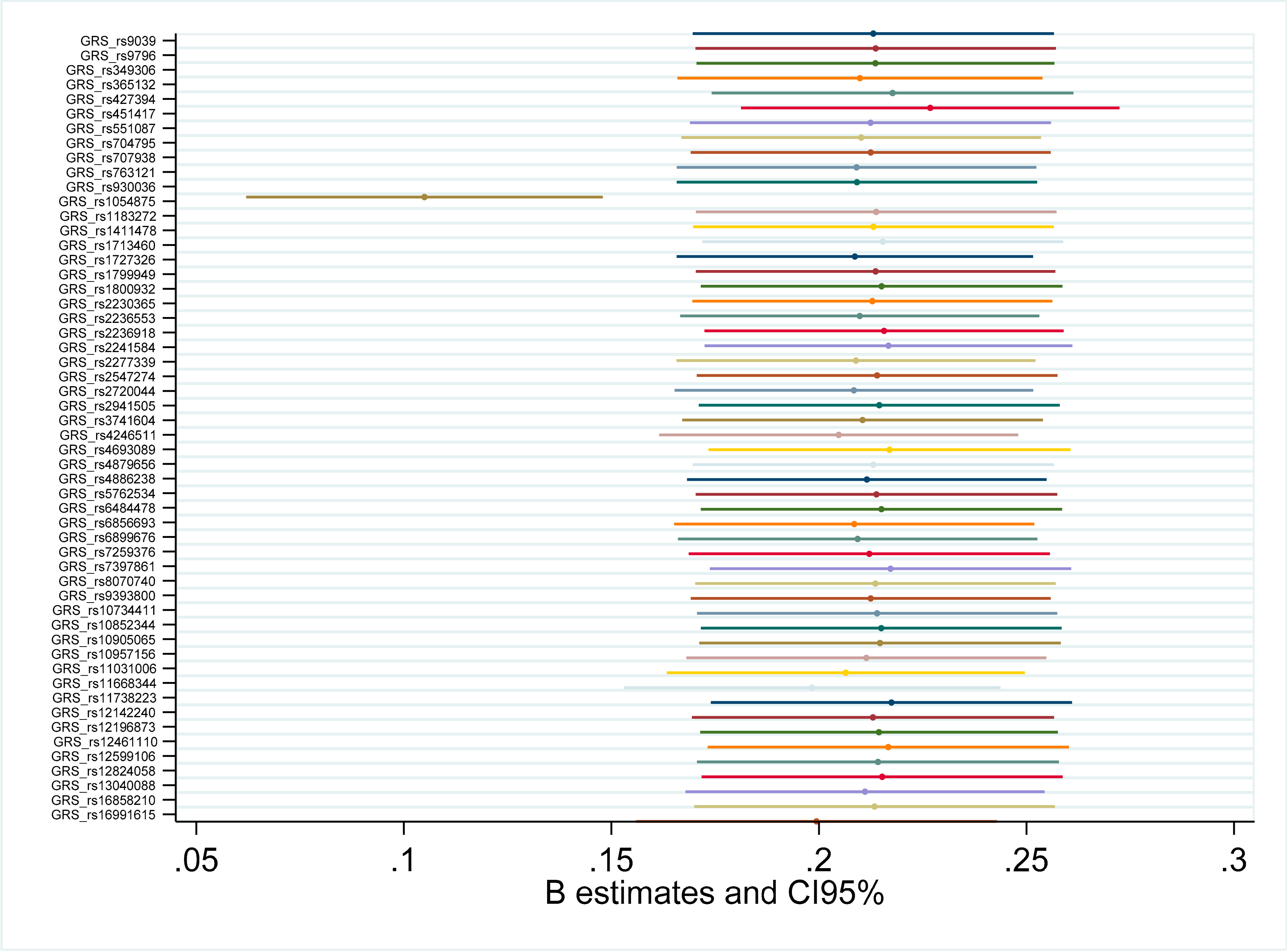
GRS_rs9039
GRS_rs9796
GRS_rs349306
GRS_rs365132
GRS_rs427394
GRS_rs451417
GRS_rs551087
GRS_rs704795
GRS_rs707938
GRS_rs763121
GRS_rs930036
GRS_rs1054875
GRS_rs1183272
GRS_rs1411478
GRS_rs1713460
GRS_rs1727326
GRS_rs1799949
GRS_rs1800932
GRS_rs2230365
GRS_rs2236553
GRS_rs2236918
GRS_rs2241584
GRS_rs2277339
GRS_rs2547274
GRS_rs2720044
GRS_rs2941505
GRS_rs3741604
GRS_rs4246511
GRS_rs4693089
GRS_rs4879656
GRS_rs4886238
GRS_rs5762534
GRS_rs6484478
GRS_rs6856693
GRS_rs6899676
GRS_rs7259376
GRS_rs7397861
GRS_rs8070740
GRS_rs9393800
GRS_rs10734411
GRS_rs10852344
GRS_rs10905065
GRS_rs10957156
GRS_rs11031006
GRS_rs11668344
GRS_rs11738223
GRS_rs12142240
GRS_rs12196873
GRS_rs12461110
GRS_rs12599106
GRS_rs12824058
GRS_rs13040088
GRS_rs16858210
GRS_rs16991615
.05
.1
.15
.2
.25
.3
B estimates and CI95%

RS-II-1

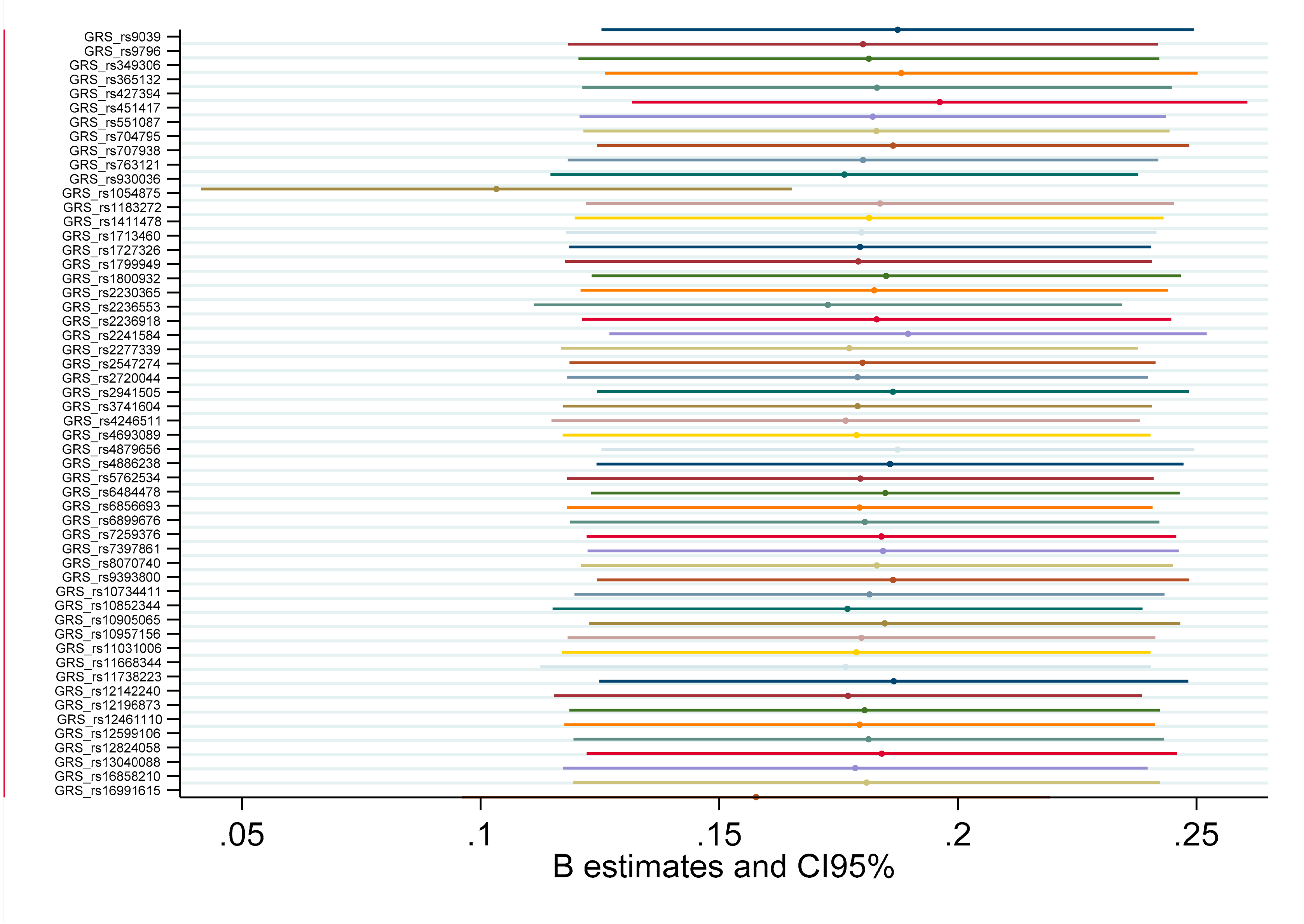
GRS_rs9039
GRS_rs9796
GRS_rs349306
GRS_rs365132
GRS_rs427394
GRS_rs451417
GRS_rs551087
GRS_rs704795
GRS_rs707938
GRS_rs763121
GRS_rs930036
GRS_rs1054875
GRS_rs1183272
GRS_rs1411478
GRS_rs1713460
GRS_rs1727326
GRS_rs1799949
GRS_rs1800932
GRS_rs2230365
GRS_rs2236553
GRS_rs2236918
GRS_rs2241584
GRS_rs2277339
GRS_rs2547274
GRS_rs2720044
GRS_rs2941505
GRS_rs3741604
GRS_rs4246511
GRS_rs4693089
GRS_rs4879656
GRS_rs4886238
GRS_rs5762534
GRS_rs6484478
GRS_rs6856693
GRS_rs6899676
GRS_rs7259376
GRS_rs7397861
GRS_rs8070740
GRS_rs9393800
GRS_rs10734411
GRS_rs10852344
GRS_rs10905065
GRS_rs10957156
GRS_rs11031006
GRS_rs11668344
GRS_rs11738223
GRS_rs12142240
GRS_rs12196873
GRS_rs12461110
GRS_rs12599106
GRS_rs12824058
GRS_rs13040088
GRS_rs16858210
GRS_rs16991615
.05
.1
.15
.2
.25
B estimates and CI95%

RS-III-1

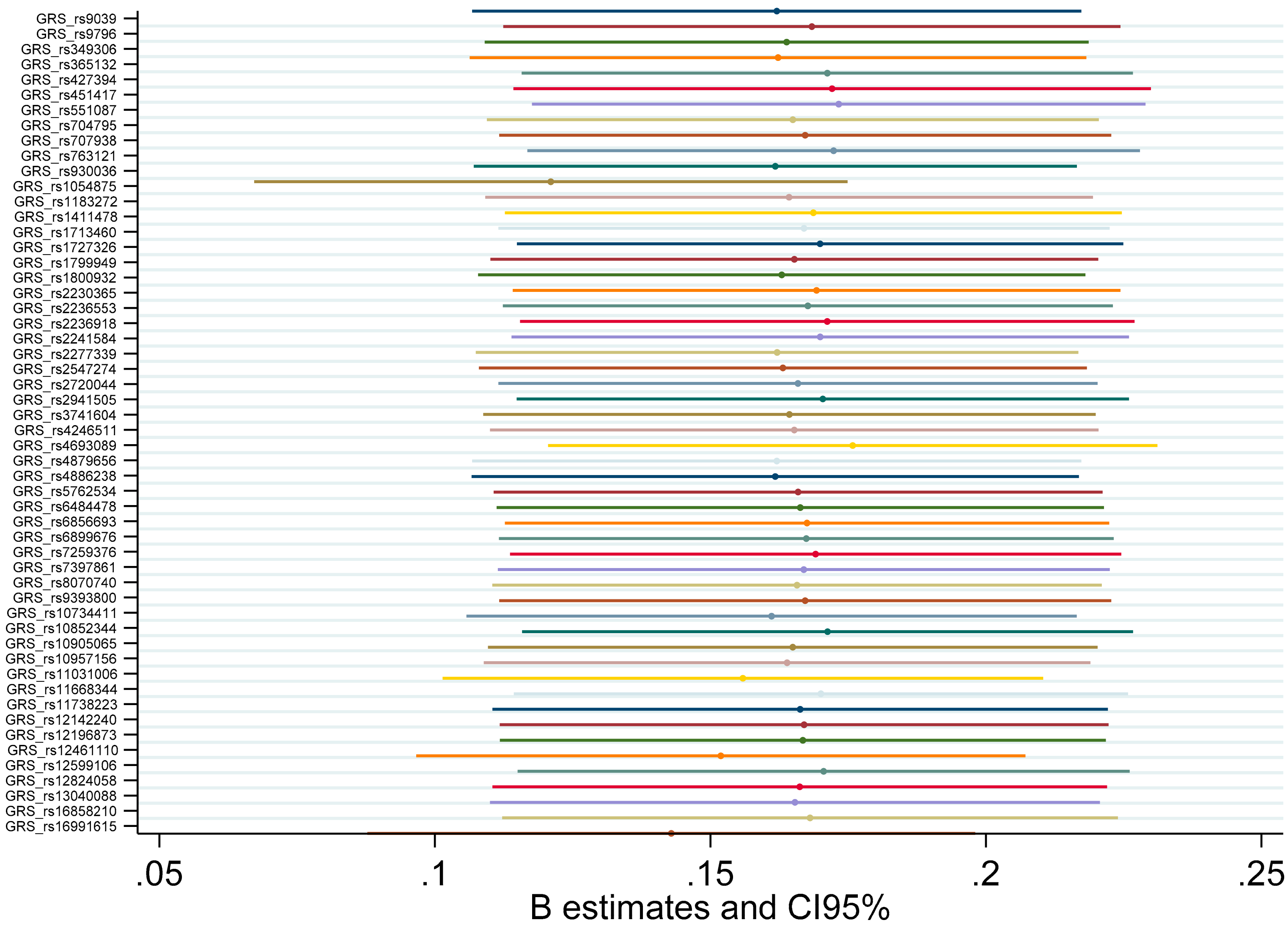
GRS_rs9039
GRS_rs9796
GRS_rs349306
GRS_rs365132
GRS_rs427394
GRS_rs451417
GRS_rs551087
GRS_rs704795
GRS_rs707938
GRS_rs763121
GRS_rs930036
GRS_rs1054875
GRS_rs1183272
GRS_rs1411478
GRS_rs1713460
GRS_rs1727326
GRS_rs1799949
GRS_rs1800932
GRS_rs2230365
GRS_rs2236553
GRS_rs2236918
GRS_rs2241584
GRS_rs2277339
GRS_rs2547274
GRS_rs2720044
GRS_rs2941505
GRS_rs3741604
GRS_rs4246511
GRS_rs4693089
GRS_rs4879656
GRS_rs4886238
GRS_rs5762534
GRS_rs6484478
GRS_rs6856693
GRS_rs6899676
GRS_rs7259376
GRS_rs7397861
GRS_rs8070740
GRS_rs9393800
GRS_rs10734411
GRS_rs10852344
GRS_rs10905065
GRS_rs10957156
GRS_rs11031006
GRS_rs11668344
GRS_rs11738223
GRS_rs12142240
GRS_rs12196873
GRS_rs12461110
GRS_rs12599106
GRS_rs12824058
GRS_rs13040088
GRS_rs16858210
GRS_rs16991615
.05
.1
.15
.2
.25
B estimates and CI95%

FIGURE S5. CAUSAL ESTIMATES DERIVED FROM INSTRUMENTAL VARIABLE ANALYSIS FOR A YEAR INCREASE IN ANM AND RISK OF BLOOD PRESSURE TRAITS USING INDIVIDUAL PARTICIPANT DATA AND EXCLUDING THE SNP RS1054875

Crude	Adjusted
**SBP**	
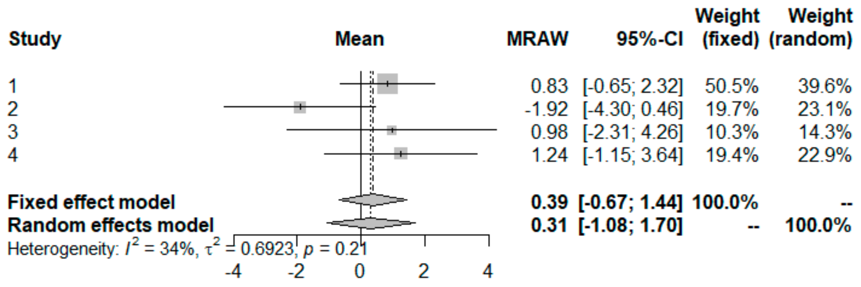	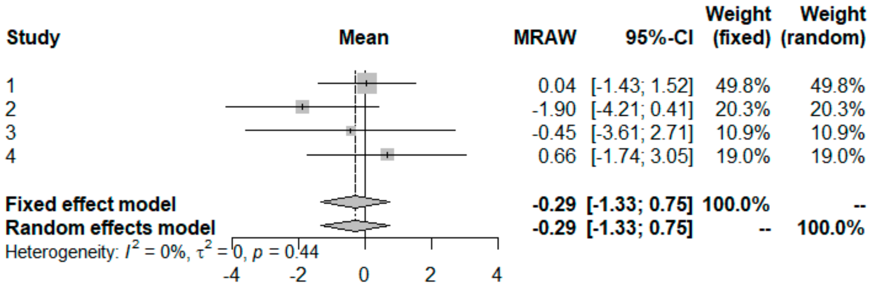
**DBP**	
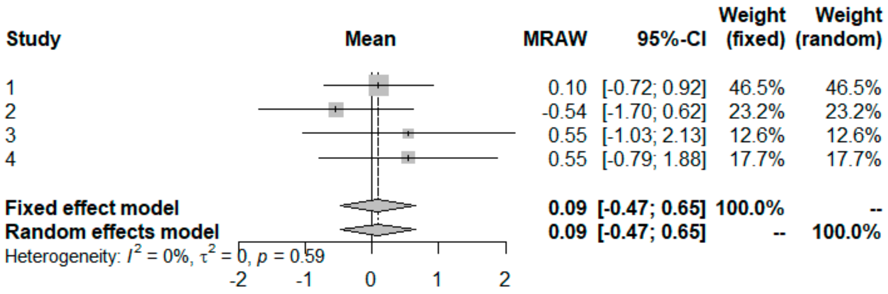	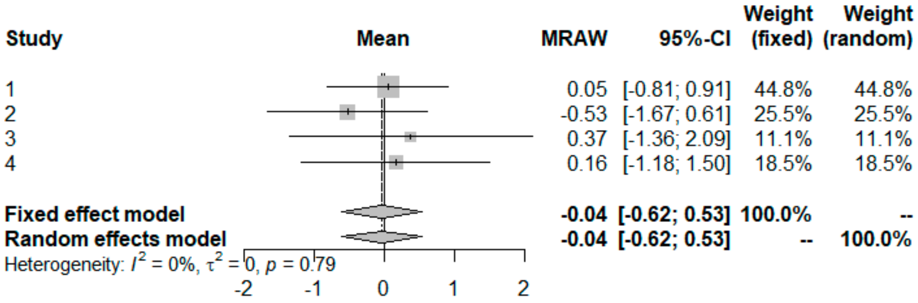
**HTA**	

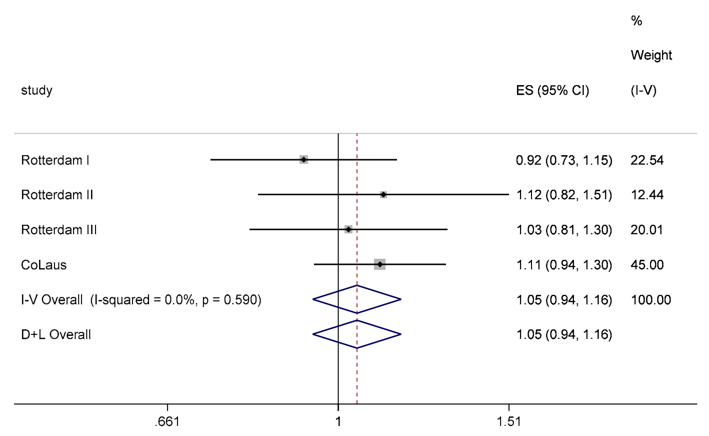
%
Weight
study
ES (95% CI)
(I-V)
Rotterdam I
0.92 (0.73, 1.15)
22.54
Rotterdam II
1.12 (0.82, 1.51)
12.44
Rotterdam III
1.03 (0.81, 1.30)
20.01
CoLaus
1.11 (0.94, 1.30)
45.00
I-V Overall (I-squared = 0.0%, p = 0.590)
1.05 (0.94, 1.16)
100.00
D+L Overall
1.05 (0.94, 1.16)
.661
1
1.51

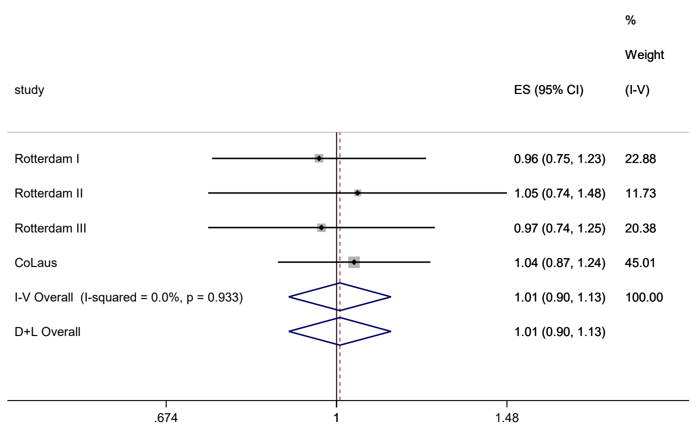
%
Weight
study
ES (95% CI)
(I-V)
Rotterdam I
0.96 (0.75, 1.23)
22.88
Rotterdam II
1.05 (0.74, 1.48)
11.73
Rotterdam III
0.97 (0.74, 1.25)
20.38
CoLaus
1.04 (0.87, 1.24)
45.01
I-V Overall (I-squared = 0.0%, p = 0.933)
1.01 (0.90, 1.13)
100.00
D+L Overall
1.01 (0.90, 1.13)
.674
1
1.48

FIGURE S6. FUNNEL PLOT OF CAUSAL ASSOCIATION BETWEEN ANM AND BP.

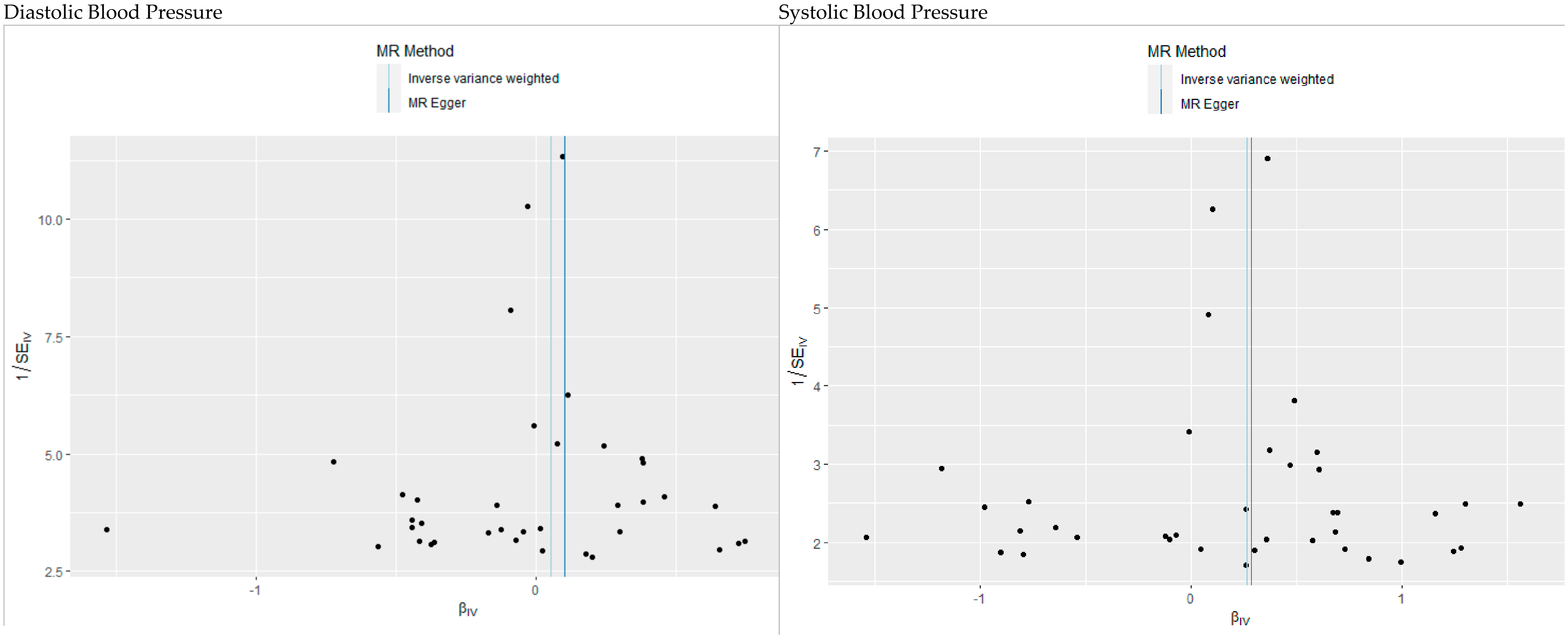

FIGURE S7. MENDELIAN RANDOMIZATION DIAGNOSTIC PLOTS

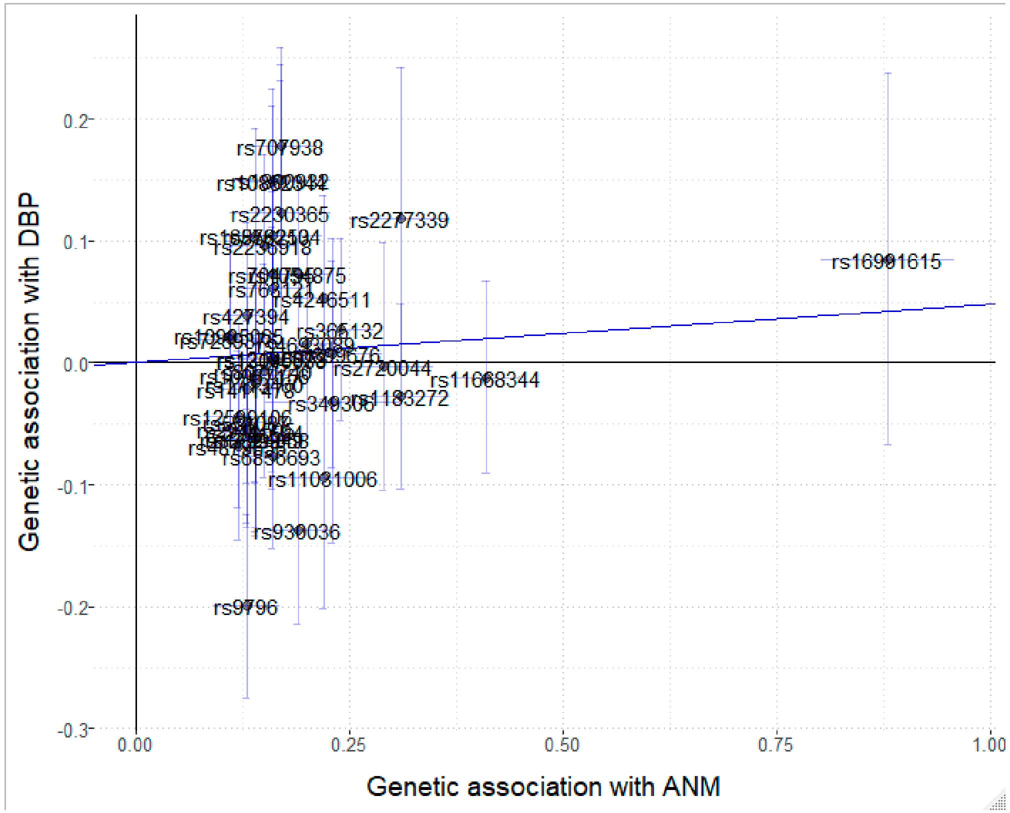

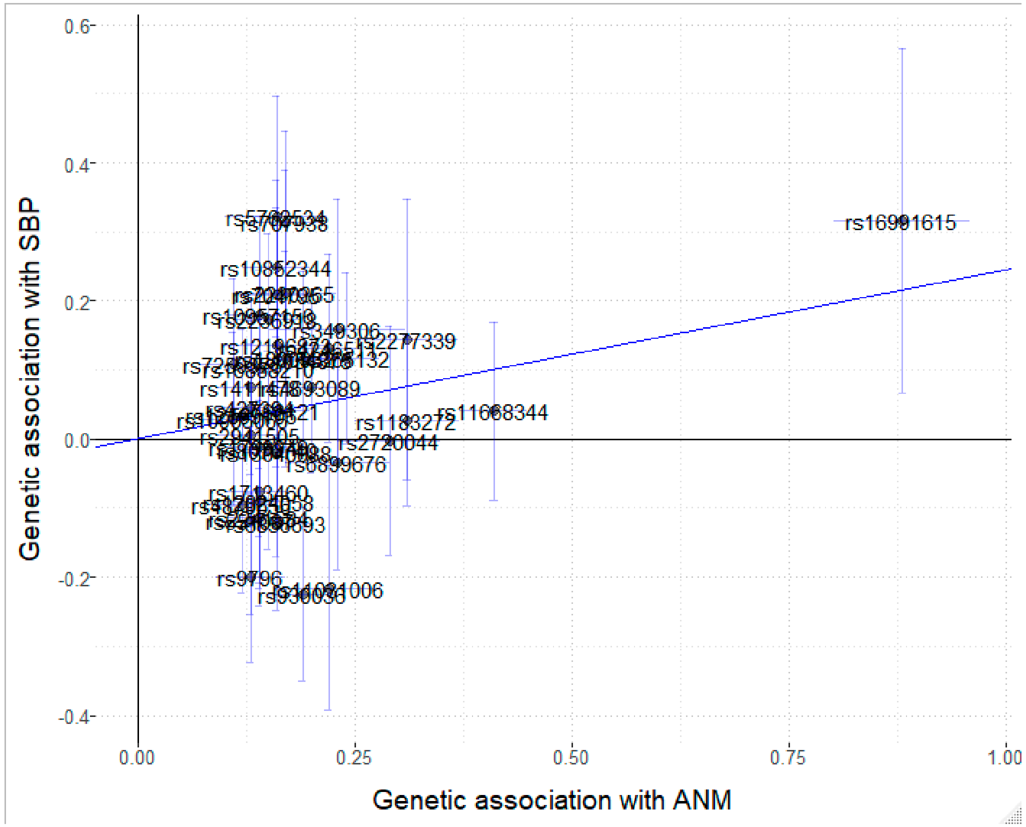

Figure S8. Leave-one-out plot to assess if a single variant is driving the association between ANM and BP

Inverse Weighted median

Figure S8a. Diastolic Blood Pressure

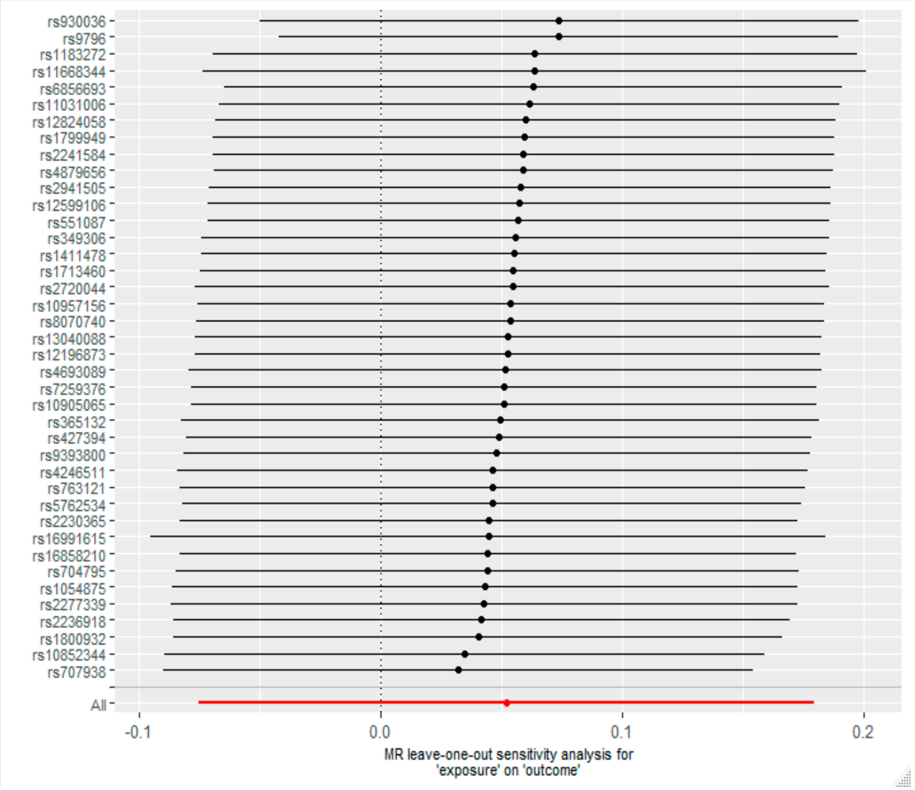

Figure S8b. Systolic Blood Pressure

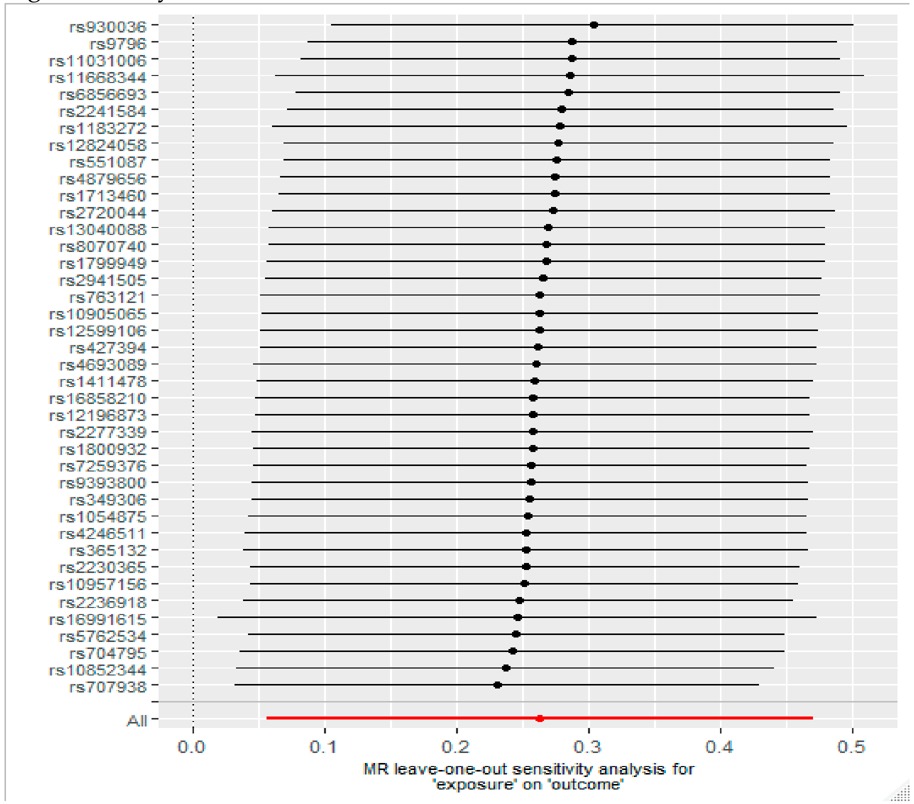

MR-Egger

Figure S8c. Diastolic Blood Pressure

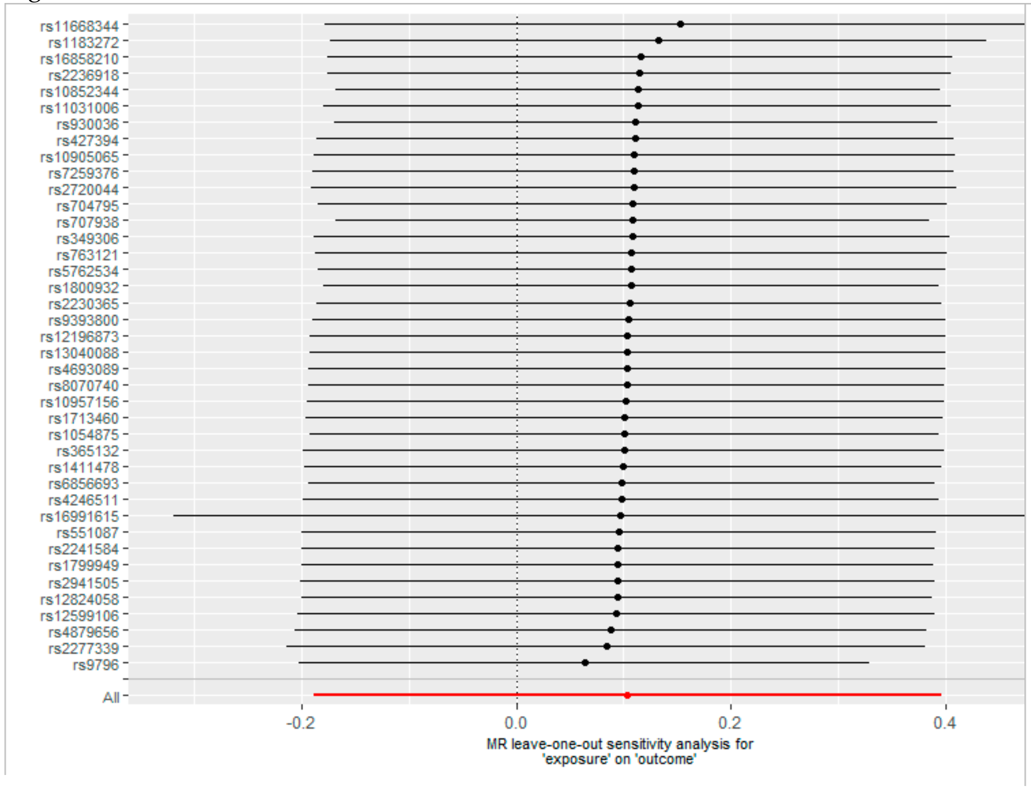

Figure S8d. Systolic Blood Pressure

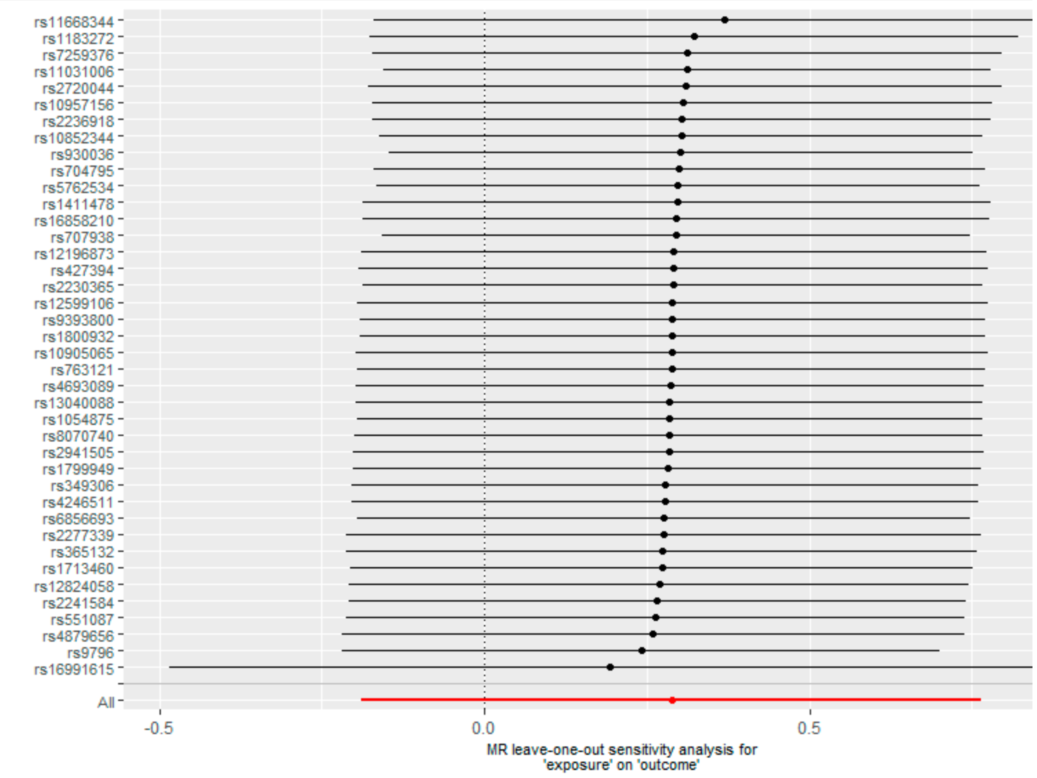

FIGURE S9. SCATTER PLOTS, LEAVE-ONE-OUT, FUNNEL PLOTS SNPS RELATED TO DDR GENES

a) Scatter plots SNPs related to DDR genes

Diastolic Blood Pressure

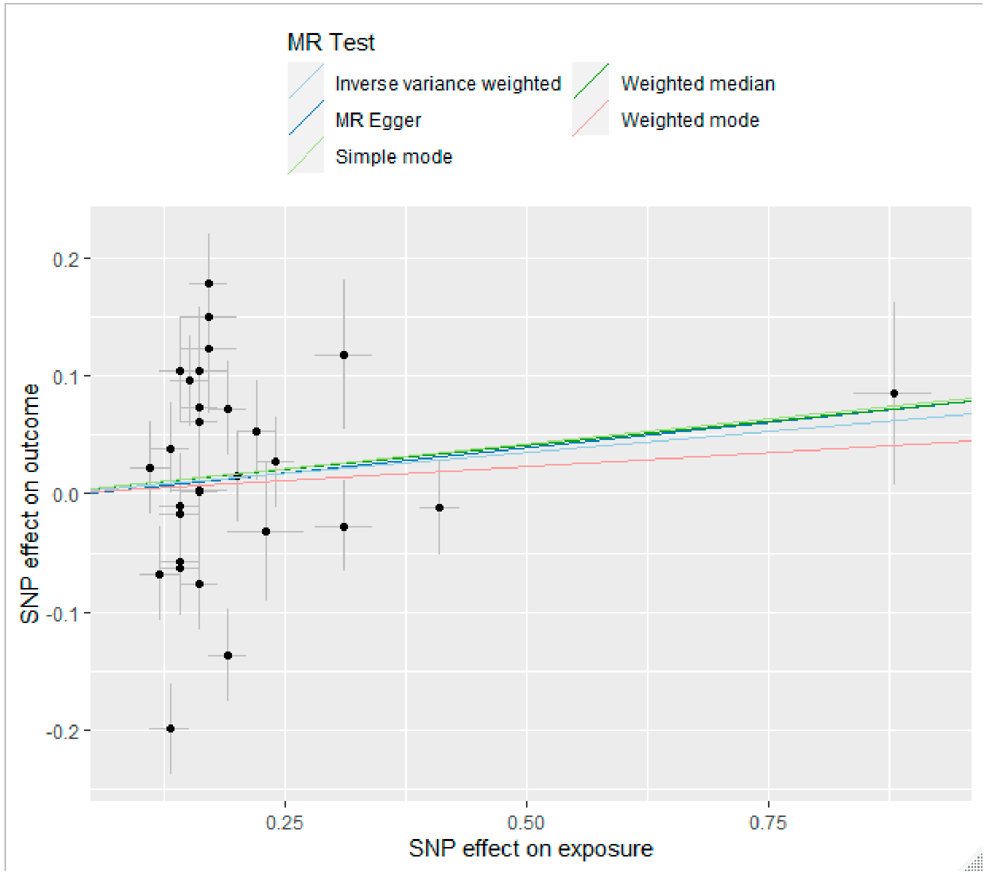

Systolic Blood Pressure

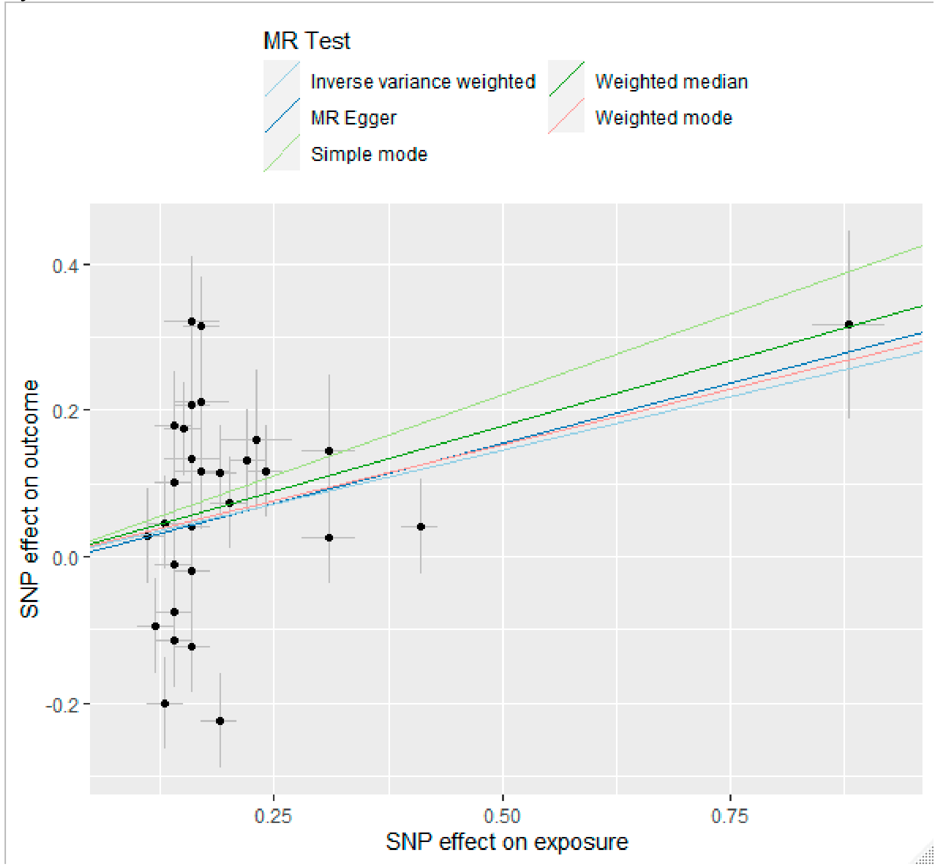

b) Leave-one-out analyses exposure and outcome SNPs related to DDR genes

Diastolic Blood Pressure 29 DDR genes

Weighted median

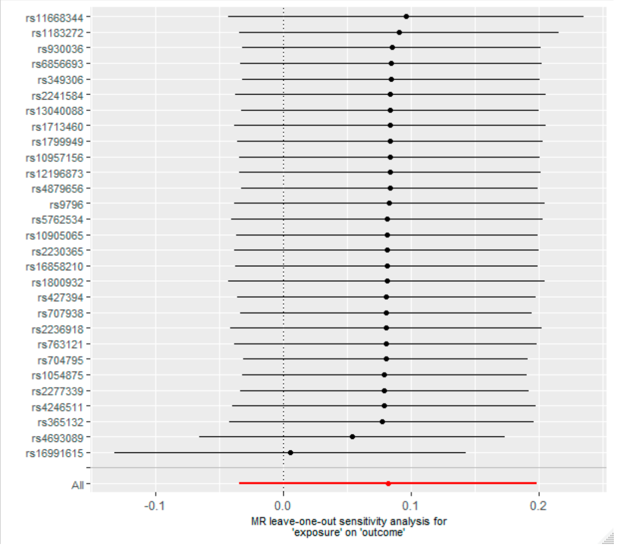

Inverse variance weighted

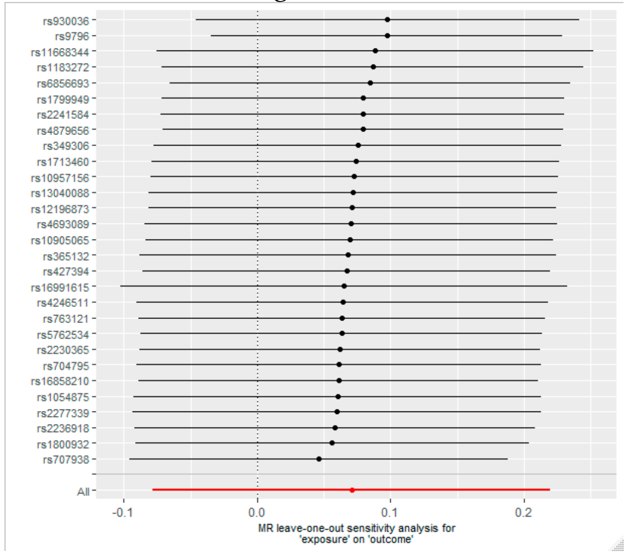

MR-Egger

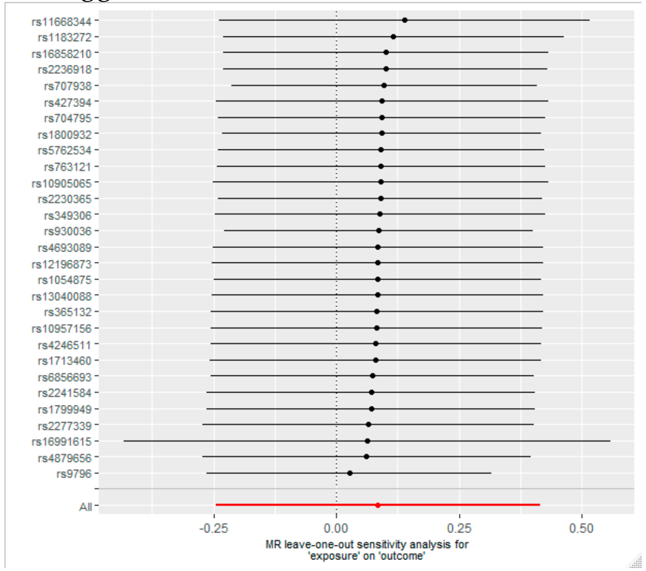

Systolic Blood Pressure 29 DDR genes

Weighted median

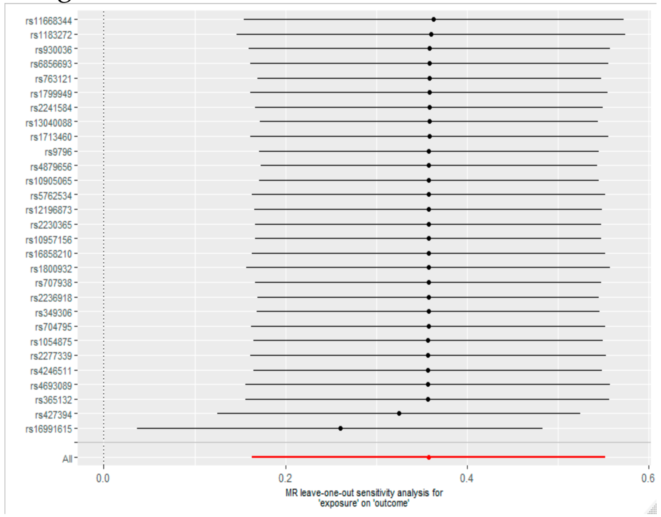

Inverse variance weighted

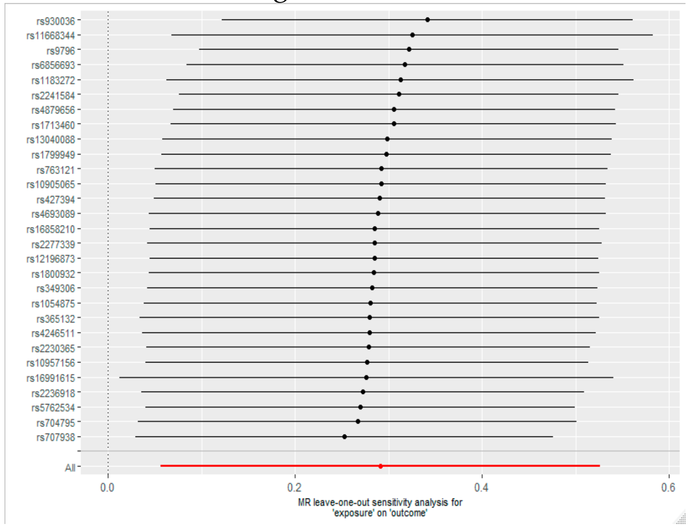

MR-Egger

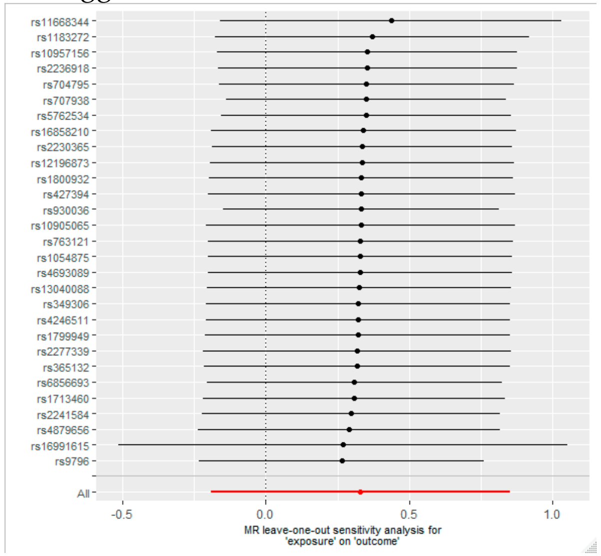

c) Funnel plot SNPs related to DDR genes

Diastolic Blood Pressure

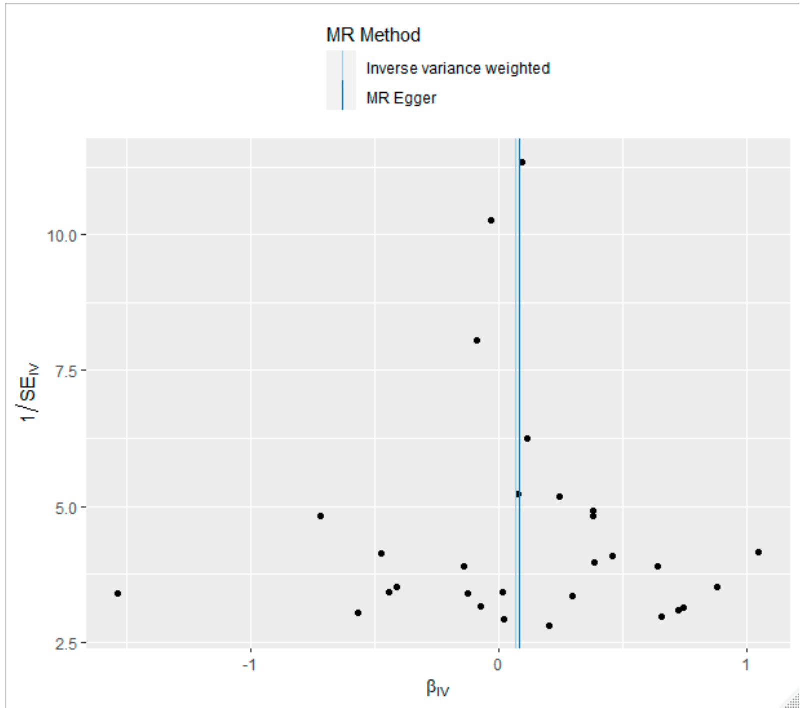

Systolic Blood Pressure

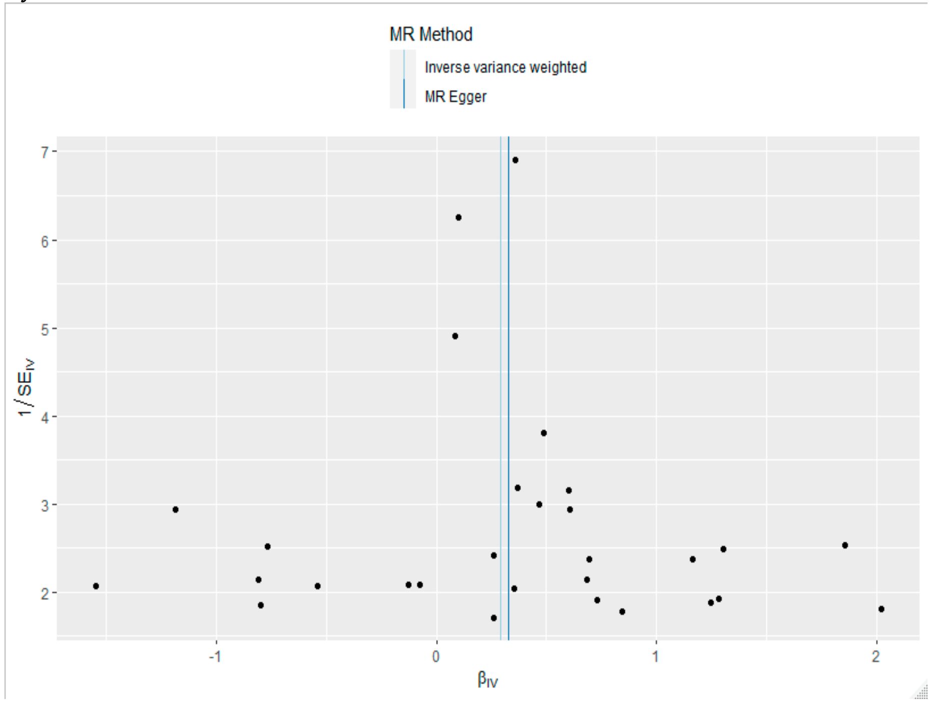

FIGURE S10. SCATTER PLOTS, LEAVE-ONE-OUT, FUNNEL PLOTS SNPS NON-RELATED TO DDR GENES

a) Scatter plots SNPs non-related to DDR genes

Diastolic Blood Pressure

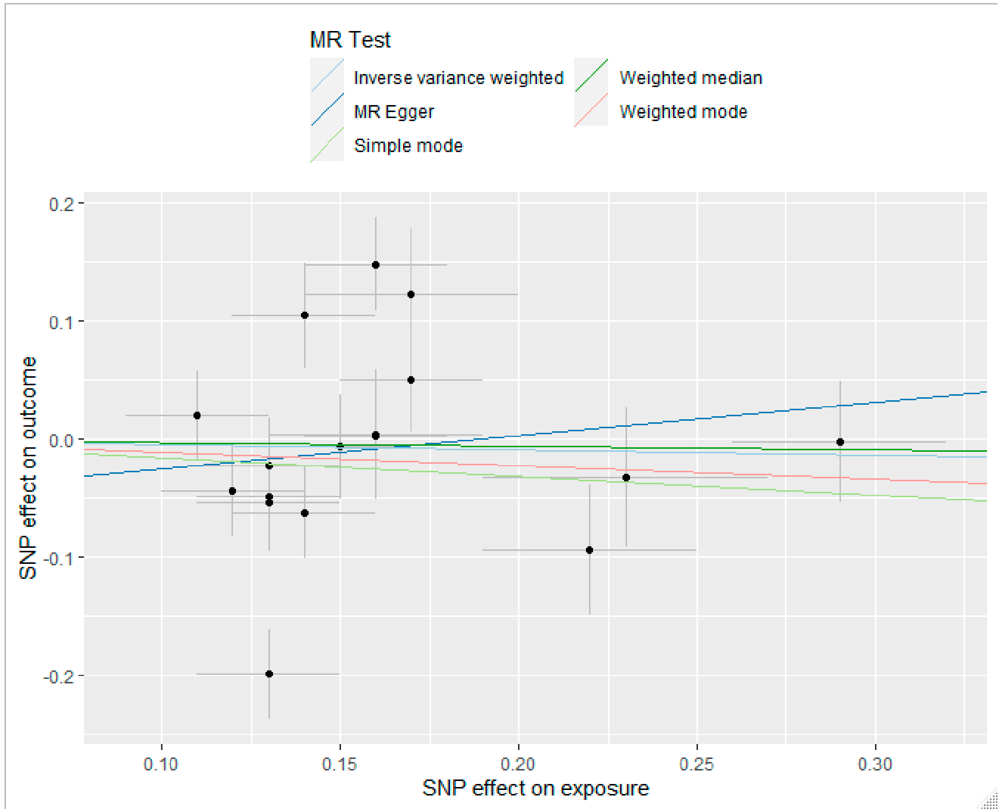

Systolic Blood Pressure

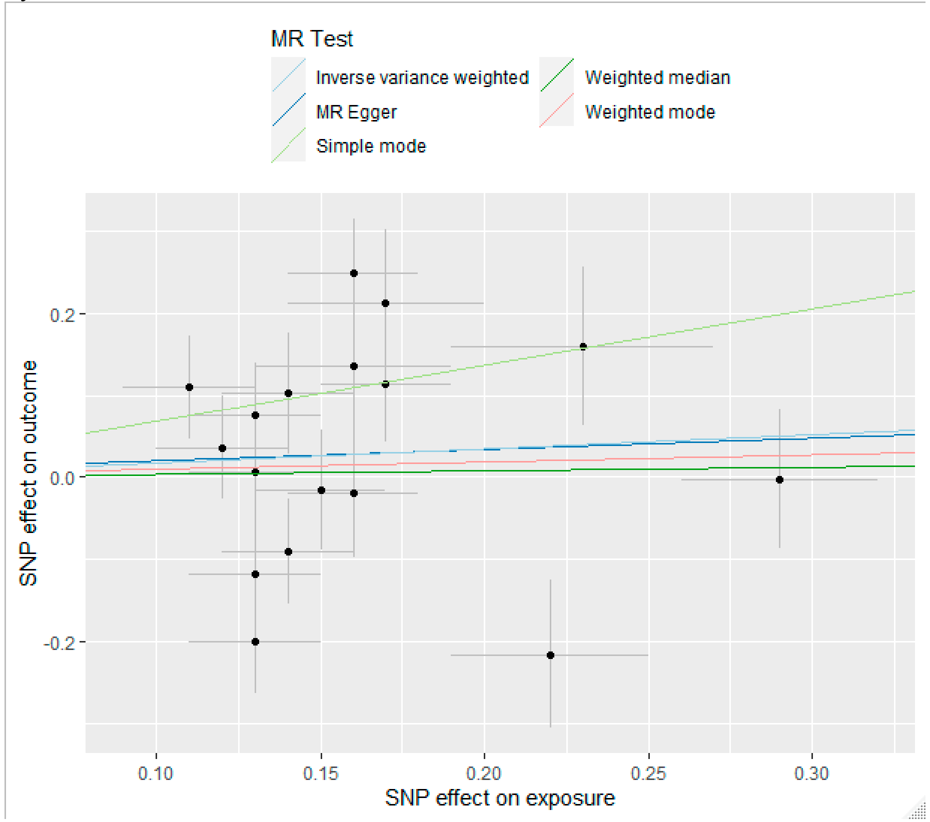

b) Leave-one-out analyses exposure and outcome SNPs non-related to DDR genes

Diastolic Blood Pressure 17 NON-DDR genes

Weighted median

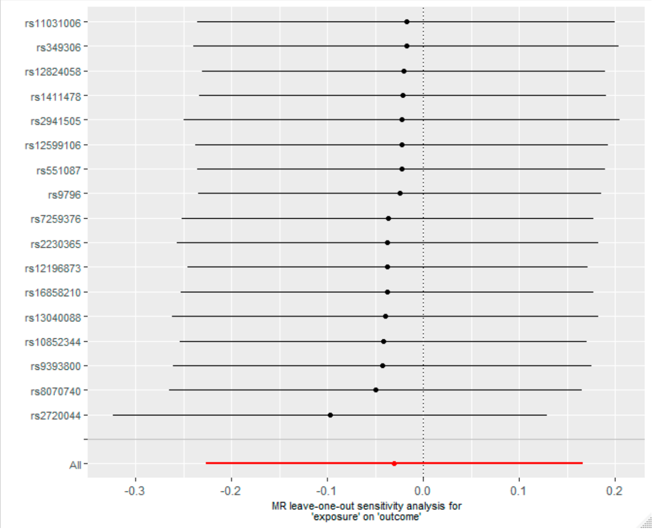

Inverse variance weighted

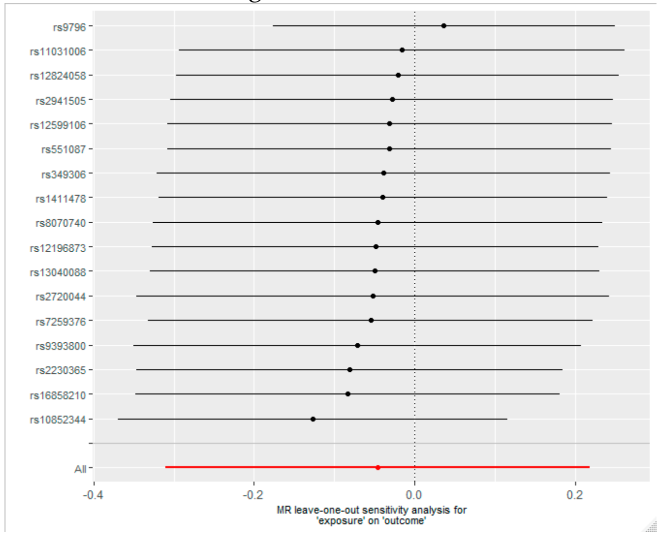

MR-Egger

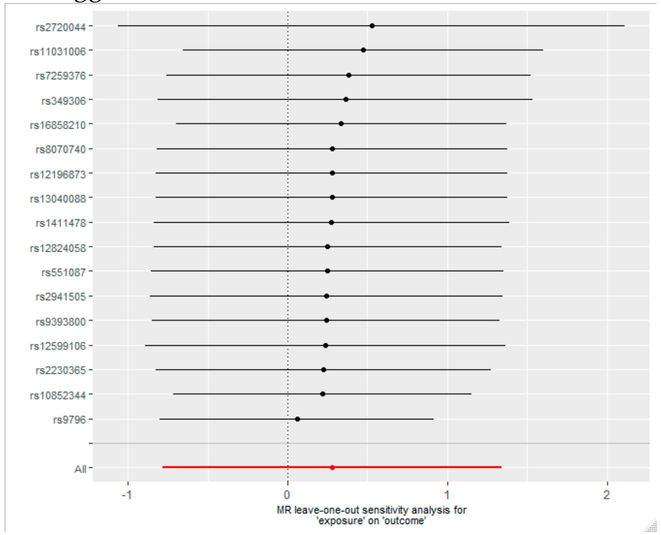

Systolic Blood Pressure 17 NON-DDR genes

Weighted median

Inverse variance weighted

MR-Egger

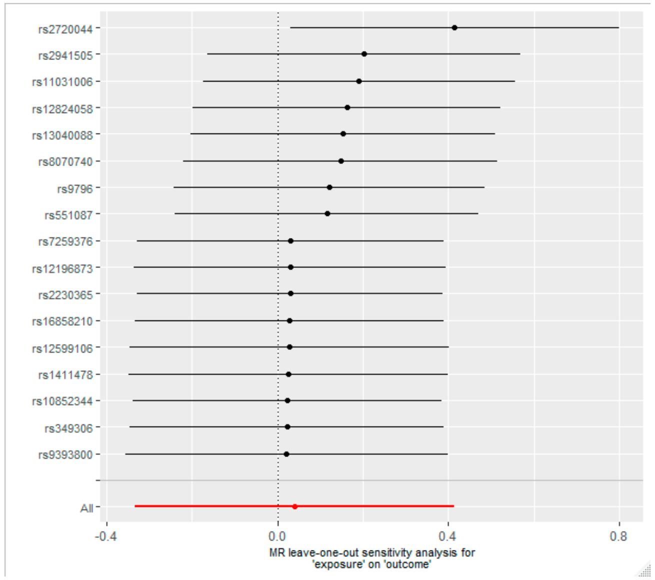

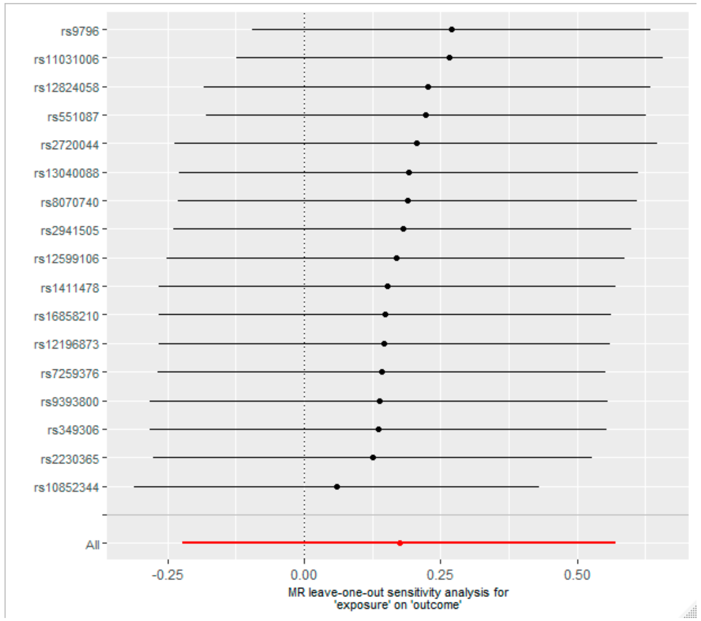

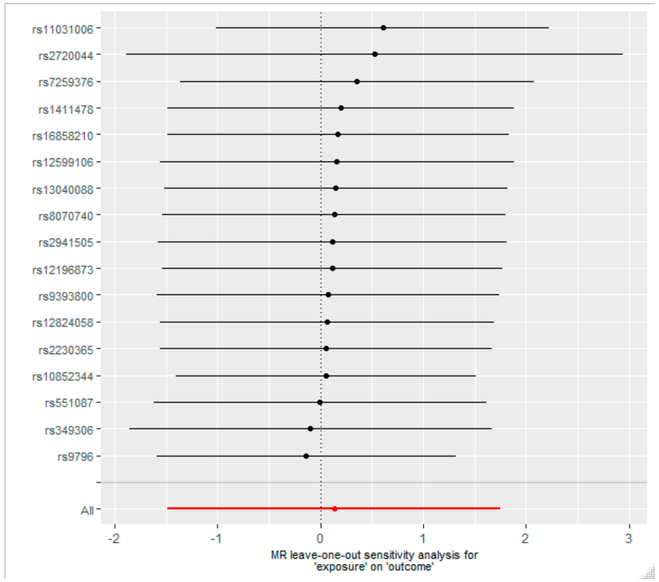

C) Funnel plot SNPs non-related to DDR genes

Diastolic Blood Pressure

Systolic Blood Pressure

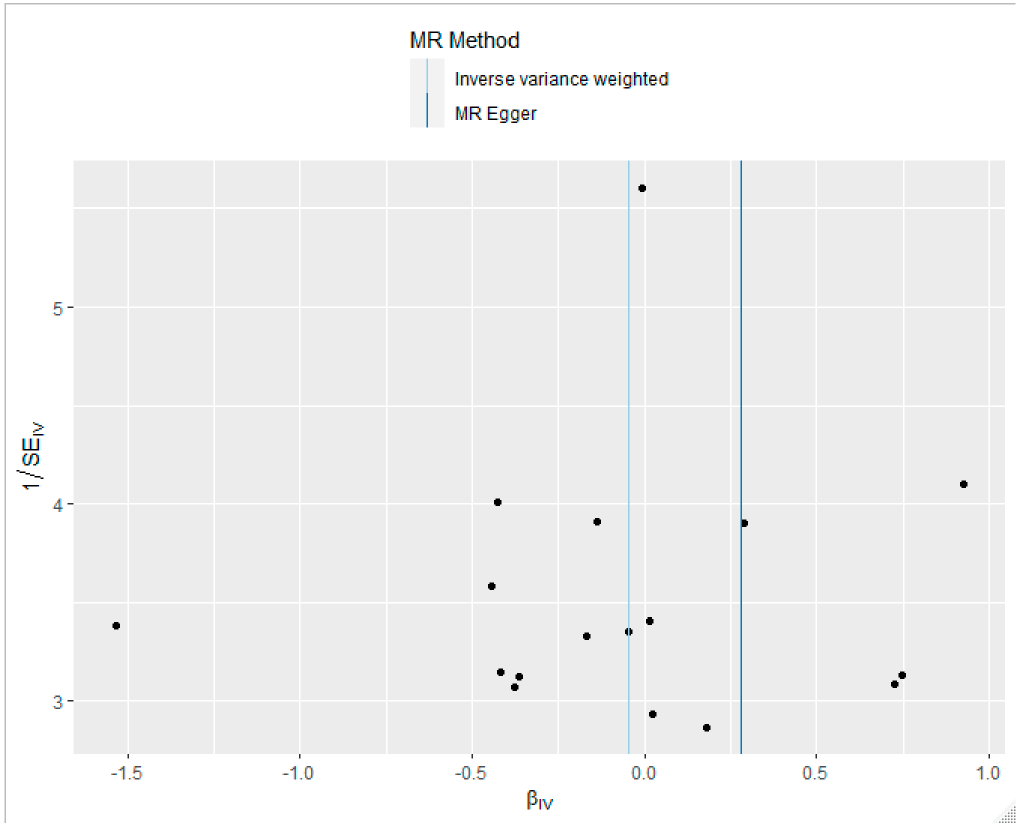
MR Method
Inverse variance weighted
MR Egger
1/SE_IV
5
4
3
-1.5
-1.0
-0.5
0.0
0.5
1.0
β_IV

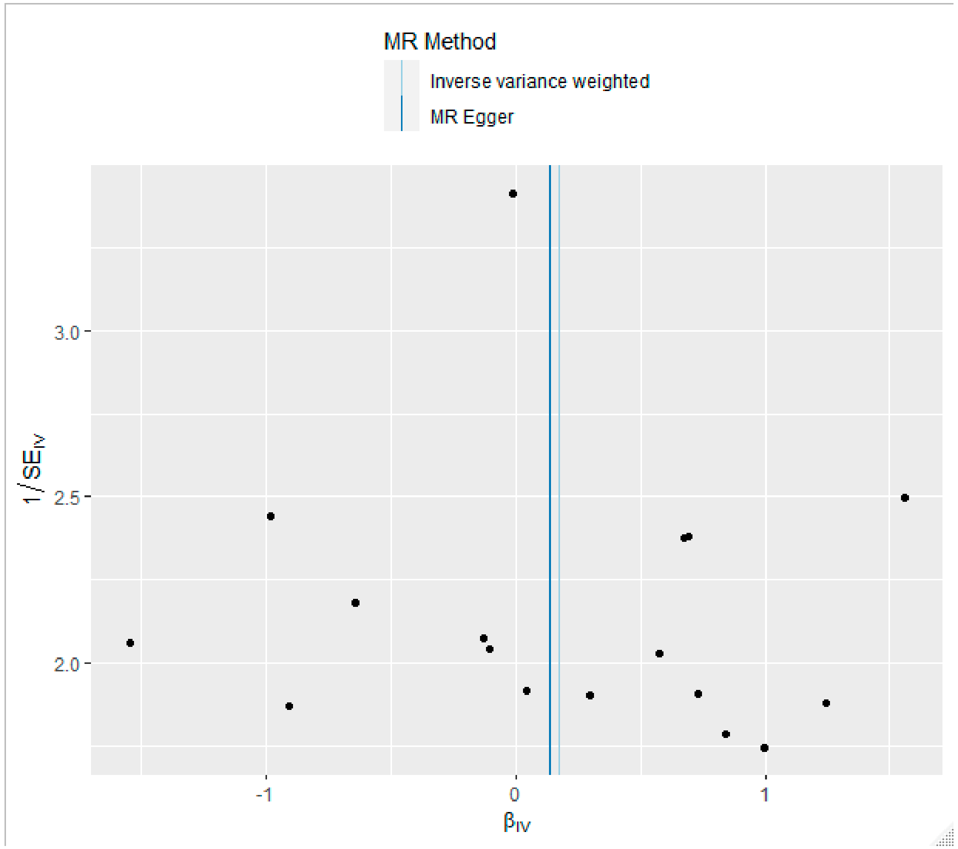
MR Method
Inverse variance weighted
MR Egger
1/SE_IV
3.0
2.5
2.0
-1
0
1
β_IV

References

1. Firmann M, Mayor V, Vidal PM, Bochud M, Pecoud A, Hayoz D, et al. The CoLaus study: a population-based study to investigate the epidemiology and genetic determinants of cardiovascular risk factors and metabolic syndrome. BMC Cardiovasc Disord. 2008;8:6.
2. Ikram MA, Brusselle G, Ghanbari M, Goedegebure A, Ikram MK, Kavousi M, et al. Objectives, design and main findings until 2020 from the Rotterdam Study. European Journal of Epidemiology. 2020:1-35.
3. van Leeuwen R, Ikram MK, Vingerling JR, Witteman JCM, Hofman A, de Jong PTVM. Blood pressure, atherosclerosis, and the incidence of age-related maculopathy: The Rotterdam Study. Invest Ophth Vis Sci. 2003;44(9):3771-7.
4. Delaneau O, Marchini J, Zagury J-F. A linear complexity phasing method for thousands of genomes. Nature methods. 2012;9(2):179-81.
5. Das S, Forer L, Schönherr S, Sidore C, Locke AE, Kwong A, et al. Next-generation genotype imputation service and methods. Nature genetics. 2016;48(10):1284-7.
6. McCarthy S, Das S, Kretzschmar W, Delaneau O, Wood AR, Teumer A, et al. A reference panel of 64,976 haplotypes for genotype imputation. Nature genetics. 2016;48(10):1279-83.
7. Day FR, Ruth KS, Thompson DJ, Lunetta KL, Pervjakova N, Chasman DI, et al. Large-scale genomic analyses link reproductive aging to hypothalamic signaling, breast cancer susceptibility and BRCA1-mediated DNA repair. Nat Genet. 2015;47(11):1294-303.
8. Sedaghat S, Pazoki R, Uitterlinden AG, Hofman A, Stricker BHC, Ikram MA, et al. Association of uric acid genetic risk score with blood pressure: the Rotterdam study. Hypertension. 2014;64(5):1061-6.
9. Kim JH. Multicollinearity and misleading statistical results. Korean journal of anesthesiology. 2019;72(6):558.
10. Davey Smith G, Hemani G. Mendelian randomization: genetic anchors for causal inference in epidemiological studies. Hum Mol Genet. 2014;23(R1):R89-98.
11. Burgess S, Smith GD, Davies NM, Dudbridge F, Gill D, Glymour MM, et al. Guidelines for performing Mendelian randomization investigations. Wellcome Open Research. 2019;4.
